# Mapping the Current and Future Noncommunicable Disease Burden in Kenya by Human Immunodeficiency Virus Status: A Modeling Study

**DOI:** 10.1093/cid/ciz1103

**Published:** 2019-11-17

**Authors:** Mikaela Smit, Pablo N Perez-Guzman, Kennedy K Mutai, Rachel Cassidy, Joseph Kibachio, Nduku Kilonzo, Timothy B Hallett

**Affiliations:** 1 Medical Research Council Centre for Global Infectious Disease Analysis, Department of Infectious Disease Epidemiology, Imperial College London, London, United Kingdom; 2 National AIDS Control Council, Nairobi, Kenya; 3 Department of Global Health and Development, London School of Hygiene and Tropical Medicine, London, United Kingdom; 4 Division of Noncommunicable Diseases, Ministry of Health, Nairobi, Kenya; 5 Faculty of Medicine, University of Geneva, Geneva, Switzerland

**Keywords:** noncommunicable diseases, model, HIV, Kenya, aging

## Abstract

**Background:**

The noncommunicable disease (NCD) burden in Kenya is not well characterized, despite estimates needed to identify future health priorities. We aimed to quantify current and future NCD burden in Kenya by human immunodeficiency virus (HIV) status.

**Methods:**

Original systematic reviews and meta-analyses of prevalence/incidence of cardiovascular disease (CVD), chronic kidney disease, depression, diabetes, high total cholesterol, hypertension, human papillomavirus infection, and related precancerous stages in Kenya were carried out. An individual-based model was developed, simulating births, deaths, HIV disease and treatment, aforementioned NCDs, and cancers. The model was parameterized using systematic reviews and epidemiological national and regional surveillance data. NCD burden was quantified for 2018–2035 by HIV status among adults.

**Results:**

Systematic reviews identified prevalence/incidence data for each NCD except ischemic heart disease. The model estimates that 51% of Kenyan adults currently suffer from ≥1 NCD, with a higher burden in people living with HIV (PLWH) compared to persons not living with HIV (62% vs 51%), driven by their higher age profile and partly by HIV-related risk for NCDs. Hypertension and high total cholesterol are the main NCD drivers (adult prevalence of 20.5% [5.3 million] and 9.0% [2.3 million]), with CVD and cancers the main causes of death. The burden is projected to increase by 2035 (56% in persons not living with HIV; 71% in PLWH), with population growth doubling the number of people needing services (15.4 million to 28.1 million) by 2035.

**Conclusions:**

NCD services will need to be expanded in Kenya. Guidelines in Kenya already support provision of these among both the general and populations living with HIV; however, coverage remains low.

## Teaser

This study collates all noncommunicable disease (NCD) data in Kenya and combines them with a model to provide the first country-level NCD estimated by Human Immunodeficiency Virus status. The results show a large and growing NCD burden, highlighting the need for NCD service expansion.

**(See the Editorial Commentary by Agan and Marconi on pages 1874–6.)**

In Kenya, the National AIDS Control Council and the Division of Noncommunicable Diseases (NCDs) at the Ministry of Health have recently called for a paradigm shift focused on providing health services for NCDs within human immunodeficiency virus (HIV) care platforms, both for people living with HIV (PLWH) and for people not living with HIV [1, 2], as a way of ensuring rapid scale-up of services for NCDs. HIV care platforms provide an opportunistic entry point for NCD services, especially now that HIV care includes NCDs and their complications [3]. They have a strong and large infrastructure within Kenya, mature partnerships, multisectoral networks, and robust and resilient capabilities [3]. Evidence from observational studies [4] and mathematical modeling studies [5–7] in other countries has consistently shown that NCDs are more prevalent among PLWH than persons not living with HIV, a disproportionate burden that is expected to increase in the coming decades.

Currently, the national-level burden of NCDs in Kenya is not well characterized, and there is no information on the prevalence by HIV status. Such estimates and forecasts will be key to inform strategic planning on integration in the country. The aim of this study is to combine original systematic reviews (SRs) and meta-analyses (MAs) of available data on NCDs in Kenya, national and regional surveillance and demographic data, and advanced modeling approaches to provide robust national-level estimates of the burden of NCDs in Kenya, by HIV status, currently and in the coming 2 decades.

## METHODS

### Systematic Reviews and Meta-analyses

Original SRs and MAs were carried out according to Meta-analyses of Observational Studies in Epidemiology (MOOSE) and Preferred Reporting Items for Systematic Reviews and Meta-Analyses (PRISMA) guidelines [8–10], summarizing in-country evidence of overall and age-specific prevalence or incidence of NCDs including cardiovascular disease (CVD; specifically ischemic heart disease [IHD] and stroke), chronic kidney disease (CKD), depression, diabetes, high total cholesterol, hypertension, and human papillomavirus (HPV) infection and related cervical intraepithelial neoplasia (CIN) (Supplementary Data 1). All NCDs were defined using standard Kenyan clinical definitions (Table 1). In brief, Medline and Embase were searched from inception to May 2018 for population-based or primary care–based studies reporting on prevalence or incidence of these NCDs in Kenya. Due to the difficulty of diagnosing cancers at the community level, cancer estimates were obtained from the Cancer Incidence in 5 Continents (version XI, International Agency for Research on Cancer [IARC]) for Kenya [11]. SRs were expanded to Tanzania, where data for Kenya were unavailable, assuming Tanzania to be comparable with regards to demography, burden of disease, and healthcare profile. Two independent reviewers screened the search results in Mendeley, with disagreement resolved by a third reviewer. MAs were carried out on overall and age-specific prevalence/incidence estimates where >1 study was found. As the SR and MA included all available observational studies in the country, the risk of bias was assessed by adhering to specific recommendations from the MOOSE checklist. Specifically, this reporting guideline’s checklist specifies that included studies should have a qualitative assessment of bias in their discussion. Details of this assessment are further discussed in Supplementary Data 1. Age-standardization prevalence (ASP) or age-standardization incidence (ASI) was calculated using standard direct method and World Health Organization standard population [12].

**Table 1.** Clinical Definitions of Noncommunicable Diseases Used for the Systematic Review

NCD	Definition
Cardiovascular disease	Study-ascertained diagnosis (eg, based on medical records or standardized acute diagnostic criteria) of ischemic heart disease or ischemic stroke
Chronic kidney disease	Estimated glomerular filtration rate ≤60 mL/min/1.73 m^2^ body surface without evidence for acute kidney failure
Depression	Study-ascertained diagnosis based on medical records or standardized questionnaire (eg, PHQ-9, CIS-R)
Diabetes, type 2	Fasting plasma glucose ≥7.0 mmol/L (126 mg/dL) or 2-h plasma glucose ≥11.1 mmol/L (200 mg/dL)
High total cholesterol	≥5.19 mmol/L (200 mg/dL)
Hypertension	Either the presence of prehypertension (≥130/80 mm Hg and <140/90 mm Hg) or overt hypertension (≥140/90 mm Hg)
HPV	Study-ascertained diagnosis of HPV infection based on DNA detection methods in cervical swap or biopsy samples
CIN lesions	Study-ascertained diagnosis of CIN 2+ (ie, CIN 2 to CIS) based on expert-assessed cytology and/or biopsy

Source: Kenya Ministry of Public Health and Sanitation.

Abbreviations: CIN, cervical intraepithelial neoplasia; CIS, carcinoma in situ; CIS-R, Clinical Interview Schedule-revised; HPV, human papillomavirus; NCD, noncommunicable diseases; PHQ-9, Patient Health Questionnaire-9.

### Model

**Figure 1.**
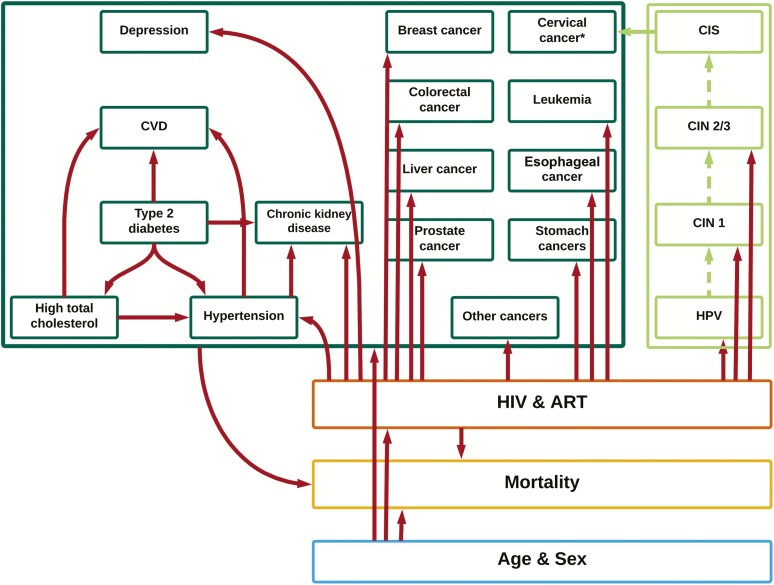
Schematic of the multidisease model for Kenya. The model simulates demography (blue), human immunodeficiency virus (HIV) epidemic (orange), and noncommunicable diseases (green), and accounts for key interactions between demographic and disease-specific factors (red arrows to individual conditions and group of conditions). *Cervical cancer risk is higher in women living with HIV, driven by the increased risk of human papillomavirus infection. Abbreviations: ART, antiretroviral therapy; CIN, cervical intraepithelial neoplasia; CIS, carcinoma in situ; CVD, cardiovascular disease; HIV, human immunodeficiency virus; HPV, human papillomavirus.

An individual-based multidisease model coded in C++ was adapted for Kenya (Figure 1). The model design and mechanism have been described previously [5], with technical details on the adaptation in Supplementary Data 2. The model simulates the whole Kenyan population, tracking an individual’s sex, age, births, deaths, HIV infection, disease progression and treatment, and the development of several NCDs. These include those from the SR and a number of cancers (including breast, cervical, colorectal, leukemia, liver, esophageal, stomach and prostate cancer, and “other” cancers where “other” refers to all cancers except the aforementioned). Cervical cancer is simulated by including a natural history model of HPV infection and progression through precancerous lesions (Figure 1; Supplementary Data 2). NCDs included those estimated to contribute the largest disease burden currently, and in the future, and for which sufficient data were available to make robust predictions, with choice of individual cancer based on those that contribute >50% of all non-AIDS-defining cancer cases in Kenya as per the IARC in 2018 [11].

Model adaptation drew on a number of data sources (Table 2). Parameters for demographic processes (birth and death rates) were from the United Nations Department of Economic and Social Affairs for Kenya [13], and parameters on HIV epidemic (HIV incidence and ART coverage) were taken from the Joint United Nations Programme on HIV/AIDS official estimates for Kenya [14]. These parameter sets changed over calendar time as reported by the data sources, for example, decreasing mortality rate over time. Parameters for HIV disease progression are based on estimates from a modeling study for the sub-Saharan Africa region [15].

**Table 2.** Summary of Model Data Sources

Parameter	Source
Demographic processes	
Age composition in 1950	United Nations World Population Prospect
Age-specific fertility rates from 1950 to 2018 (by 5-y periods)	
Age- and-sex-specific death rates from 1950 to 2018 (by 5-y periods)	
HIV epidemic	
Annual age- and-sex-specific HIV incidence from 1975 to 2016	UNAIDS estimates for Kenya
No. of people starting ART by CD4 count between 2000 and 2016	
HIV disease progression	
CD4 distribution at seroconversion by age and sex	Mangel et al, AIDS (2017) estimates for sub-Saharan Africa
CD4 count progression rate by sex	
Age- and-sex-specific mortality rate by CD4 count (ART-naive)	
Cause-specific mortality	
Risk ratio for HIV, CKD, diabetes, IHD, and stroke; breast, cervical, colorectal, leukemia, liver, esophageal, stomach, and prostate cancers^a^; and other cancers^b^	Global Burden of Disease 2016 estimates for Kenya
Case-fatality of stroke and IHD^a^	
NCD epidemic	
Age-specific NCD incidence of CKD, depression, diabetes, high total cholesterol, hypertension, and stroke; breast, cervical, colorectal, leukemia, liver, esophageal, stomach, and prostate cancers^a^; and other cancers^b^	IARC 2012 cancer estimates for Kenya; systematic review and meta-analysis for NCDs other than cancer; European CVD Statistics 2017 for myocardial infraction: IHD hazard ratio
Relative risk of IHD vs stroke incidence	
Duration of depressive episode^c^	
Probability of cervical disease progression, clearance^d^	
Rate of progression through cervical disease^d^	
Risk ratio of cervical disease progression for people living with HIV	

Abbreviations: ART, antiretroviral therapy; CKD, chronic kidney disease; CVD, cardiovascular disease; HIV, human immunodeficiency virus; IARC, International Agency for Research on Cancer; IHD, ischemic heart disease; NCD, noncommunicable disease; UNAIDS, Joint United Nations Programme on HIV/AIDS.

^a^These parameters were inferred by fitting to the data.

^b^“Other” refers to all cancers except the aforementioned.

^c^Model assumption.

^d^Stage-specific parameters were fitted, where no data were available.

Age-specific incident rates for each NCD and a fixed excess relative risk of death for each cause were inferred by fitting to data on incidence (for stroke and cancers) from the SR and IARC (for cancers), prevalence (for the remaining NCDs) from the SR, and data on deaths by cause from the 2016 Global Burden of Disease (GBD) [16] based on prespecified functional models for all persons. Hypertension and depression were assumed not to be an independent cause of death in the model, while the model assumes an additional instantaneous risk of death for stroke and IHD. Recurrent depression but only first CVD event are modeled. Of note, as no data was found on IHD in Kenya or Tanzania, the model assumed a 4-fold increased incidence compared to stroke based on a large cross-European study of CVD incidence [17].

The model is run forward over time under further assumptions about how risk of NCD acquisition varies according to underlying conditions (Table 3). For example, an older individual or a person with pre-existing hypertension is more likely to develop a CVD compared to a younger individual without hypertension. These associations are based on in-depth literature reviews (Figure 1; Table 3).

**Table 3.** Model Parameters Defining Relative Risk of Developing Individual Noncommunicable Diseases (NCDs) Given Pre-existing NCD or Human Immunodeficiency Virus Infection

Association	Hazard Ratio (95% CI)	Reference: Setting
Non-HIV-related		
Incidence of stroke given pre-existing diabetes vs stroke with no pre-existing diabetes	2.431 (1.483–2.492)	Worm et al [18]: Europe, Argentina, Australia, US
Incidence of stroke given pre-existing hypertension vs stroke with no pre-existing hypertension	1.426 (.498–1.462)	Worm et al [18]: Europe, Argentina, Australia, US, Netherlands
Onset of hypertension given pre-existing diabetes vs hypertension with no pre-existing diabetes	1.440 (1.419–1.464)	Smit et al [19]: Netherlands
CKD given pre-existing diabetes vs CKD with no pre-existing diabetes	1.450 (1.405–2.415)	Mocroft et al [20]: Europe, Argentina, Israel
CKD given pre-existing hypertension vs CKD with no pre-existing diabetes	1.469 (1.426–2.427)	Mocroft et al [20]: Europe, Argentina, Israel
HIV-related		
Hypertension given HIV infection vs hypertension without HIV infection	1.449	Schouten et al [21]: Netherlands
CKD given HIV infection vs CKD without HIV infection	2.04	Schouten et al [21]: Netherlands
Depression given HIV infection vs depression without HIV infection	3.1	Do et al [22]: US
HPV infection given HIV infection and being ART-naive or on ART for <2 y vs HPV infection without HIV infection or with HIV infection and on ART for 2+ y	1.63 (1.26–2.11)	Looker et al [23]: global systematic review and meta-analysis
Clearance of HPV infection given HIV infection and being ART-naive or on ART <2 y vs clearance of HPV infection without HIV or with HIV infection and on ART for 2+ y	0.52 (.62–.84)	Looker et al [23]: global systematic review and meta-analysis
Risk of transitioning from HPV to CIN2/3 with HIV infection and being ART-naive or on ART <2 y vs risk of transitioning from HPV to CIN2/3 without HIV or with HIV infection and on ART for 2+ y	1.32 (1.10–1.58)	Liu et al [24]: global systematic review and meta-analysis
Cancer (type-specific, excluding cervical cancer) given HIV infection vs cancer without HIV infection		Hernández-Ramírez et al [25]: registry- linkage study from US cohorts of PLWH, compared to the general population, from 1996 to 2012
Breast	0.7	
Colorectal	0.6	
Leukemia	1.2	
Liver	3.2	
Esophageal	1.2	
Prostate	0.5	
Stomach	0.7	
Other^a^	1.2	

Abbreviations: ART, antiretroviral therapy; CIN, cervical intraepithelial neoplasia; CKD, chronic kidney disease; HIV, human immunodeficiency virus; HPV, human papillomavirus; PLWH, people living with human immunodeficiency virus; US, United States.

^a^Referring to all non-AIDS-defining cancers other than the aforementioned, and cervical cancer.

The model runs from 1950 to 2035, with the period from 1950 to 2015 used to carry out a number of model checks to ensure the model output is robust (Supplementary Data 2). The model is used to generate NCD estimates from 2018 to 2035 by HIV status. Projections assume medium variance demographic projections, HIV incidence to remain stable at 2017 levels, and that ART coverage increases steadily to reach a level of coverage consistent with 90-90-90 targets. Patterns of underlying risk factors for NCD are assumed to remain unchanged, and treatment for NCDs was not explicitly simulated; instead the model implicitly assumes that current treatment coverage will remain constant over time. Sensitivity analyses were carried out to explore the impact of varying the age-specific incidence of individual NCDs by ±10% (Supplementary Data 2). All results are reported in adult populations (aged ≥18 years) and based on an average of 100 model runs.

## RESULTS

### Systematic Review and Meta-analysis

A summary of the NCD estimates for Kenya collated by the SRs and MA is presented in Table 4. The SRs identified studies for most of the NCDs, except IHD. No Kenyan studies reporting on stroke incidence or age-specific prevalence of CKD were identified, while one was identified for each outcome when the SR was expanded to include Tanzania. The SRs found few studies reporting prevalence or incidence by HIV status, the notable exceptions being HPV and related CIN and CKD.

**Table 4.** Summary of Noncommunicable Disease Prevalence and Incidence Data for Kenya, as Collated Through Systematic Reviews and Meta-analyses

Disease and Age Group, y	Age-specific Prevalence, % (95% CI)	Crude Prevalence, % (95% CI)	Age-standardized Prevalence, % (95% CI)	Country/Source	Study Period	Reference
Chronic kidney disease^a^ 18–29 30–39 40–49 50–59 ≥60	5.9 (.7–11.2) 6.2 (3.5–8.9) 10.4 (6.5–14.3) 12.4 (8.1–16.6) 14.4 (7.6–21.3)	10.1 (6.2–14.0)	9.2 (4.6–13.8)	Kenya and Tanzania, pooled using meta-analysis	2014	[26–28]
Depression (episode in last 12 mo)^a^ 18–29 30–44 45–59 60–69 70–79 ≥80	3.2 (2.5–3.9) 9.0 (7.3–10.7) 4.9 (3.1–6.7) 7.4 (3.3–11.5) 12.8 (5.4–20.2) 4.4 (0–12.8)	8.5 (5.1–11.8)	6.3 (4.3–8.5)	Kenya	2003	[29–36]
Type 2 diabetes^a^ 18–29 30–39 40–49 50–59 ≥60	1.6 (.0–3.3) 2.7 (1.8–3.5) 4.6 (3.0–6.2) 5.8 (4.8–6.8) 7.6 (4.1–11.2)	5.2 (3.0–7.3)	4.0 (2.3–5.7)	Kenya, pooled using meta-analysis	2015	[37–41]
High total cholesterol^a^ 18–29 30–39 40–49 50–59 ≥60	8.5 (2.3–14.7) 11.6 (9.7–13.4) 10.5 (4.0–17.1) 14.6 (8.9–20.4) 18.1 (14.0–22.2)	11.7 (11.3–12.0)	12.1 (7.2–17.0)	Kenya, pooled using meta-analysis	2015	[40, 42, 43]
Hypertension^a^ 18–29 30–39 40–49 50–59 ≥60	13.8 (9.6–18.1) 18.9 (13.9–24.0) 29.6 (23.4–35.9) 42.6 (36.8–48.4) 52.5 (47.6–57.4)	25.6 (21.1–30.1)	28.7 (23.6–33.8)	Kenya, pooled using meta-analysis	2015	[40, 42–65]
Cervical HPV infection in the overall population^a^ 15–24 25–29 30–34 35–39 ≥40	31.9 (20.8–43.0) 32.9 (18.7–47.2) 29.1 (16.8–41.5) 33.8 (14.0–53.5) 28.0 (14.1–41.9)	36.5 (23.7–49.3)	30.1 (16.4–43.8)	Kenya	2001	[66–70]
Cervical HPV infection in the population living with HIV^a^ 15–24 25–29 30–34 35–39 ≥40	69.6 (42.8–96.5) 64.3 (44.5–84.1) 58.2 (28.5–87.8) 60.0 (39.3–80.8) 56.0 (34.8–77.3)	54.7 (38.2–71.3)	60.6 (37.5–83.8)	Kenya	2006	[68, 70–74]
CIN 2/3 lesions in the overall population^a^ 15–24 25–29 30–34 35–39 ≥40	4.0 (.1–7.8) 7.5 (4.1–10.8) 9.2 (5.2–13.3) 10.4 (5.1–15.7) 5.7 (2.6–8.8)	5.7 (3.5–8.0)	6.3 (2.7–9.8)	Kenya	1997	[66–70]
CIN 2/3 lesions in the population living with HIV^a^ 15–24 25–29 30–34 35–39 ≥40	3.3 (.7–5.8) 13.3 (10.0–15.0) 8.4 (6.3–9.4) 8.6 (6.7–9.4) 8.2 (3.7–10.0)	13.4 (7.3–19.5)	7.7 (4.3–9.5)	Kenya	2013	[68, 70–75]
Stroke 18–44 45–54 55–64 65–74 75–84 ≥85	9.3 (4.7–16.6) 91.1 (74.4–109.7) 220.5 (193.8–249.3) 629.1 (584.0–677.5) 1432.6 (1361.7–1506.1) 1933.7 (1850.2–2019.9)	83.9 (67.7–101.9)	114.8 (102.7–129.4)	Tanzania	2003–2006	[76]

Study period year is based on year of the relevant study, where >1 study was combined in a meta-analysis mean calendar year was calculated from included studies.

Abbreviations: CI, confidence interval; CIN, cervical intraepithelial neoplasia; HIV, human immunodeficiency virus; HPV, human papillomavirus.

^a^Collated through systematic review and meta-analysis.

The largest number of studies (n = 22) were found for hypertension, which also had the highest prevalence with the MA calculating a crude prevalence of 25.6% (95% confidence interval [CI], 21.1%–30.1%) and ASP of 28.7% (95% CI, 23.6%–33.8%). High total cholesterol, CKD, and depression were also prevalent, with a crude prevalence of 11.7% (95% CI, 11.3%–12.0%), 10.1% (95% CI, 6.2%–14.0%), and 9.0% (95% CI, 8.1%–9.9%), respectively; and ASP of 12.1% (95% CI, 7.2%–17.0%), 9.2% (95% CI, 4.6%–13.8%), and 6.4% (95% CI, 4.3%–8.5%), respectively.

### Current Demographic and Epidemiological Estimates

The model estimates that out of 49.6 million Kenyans, 1.6 million are PLWH. Mean age in the general population is estimated to be 22 years old, compared to 33 years old among PLWH.

According to the model, an estimated 51% of Kenyan adults currently suffer from ≥1 NCDs (ie, hypertension, high total cholesterol, diabetes, depression, CVD, CKD, and/or cancer). The burden is estimated to be higher among adult PLWH compared to adults not living with HIV (62% in PLWH vs 51% in HIV negative). This is mainly driven by the older age of PLWH and to a lesser extent by HIV-related risk for NCDs (ASP of diabetes is 1.0% in PLWH and 1.1% in adults not living with HIV, for which HIV is not assumed to be a risk factor vs ASP of hypertension is 34.6% in PLWH and 28.0% in adults not living with HIV, for which HIV is assumed to be a risk factor).

**Figure 2.**
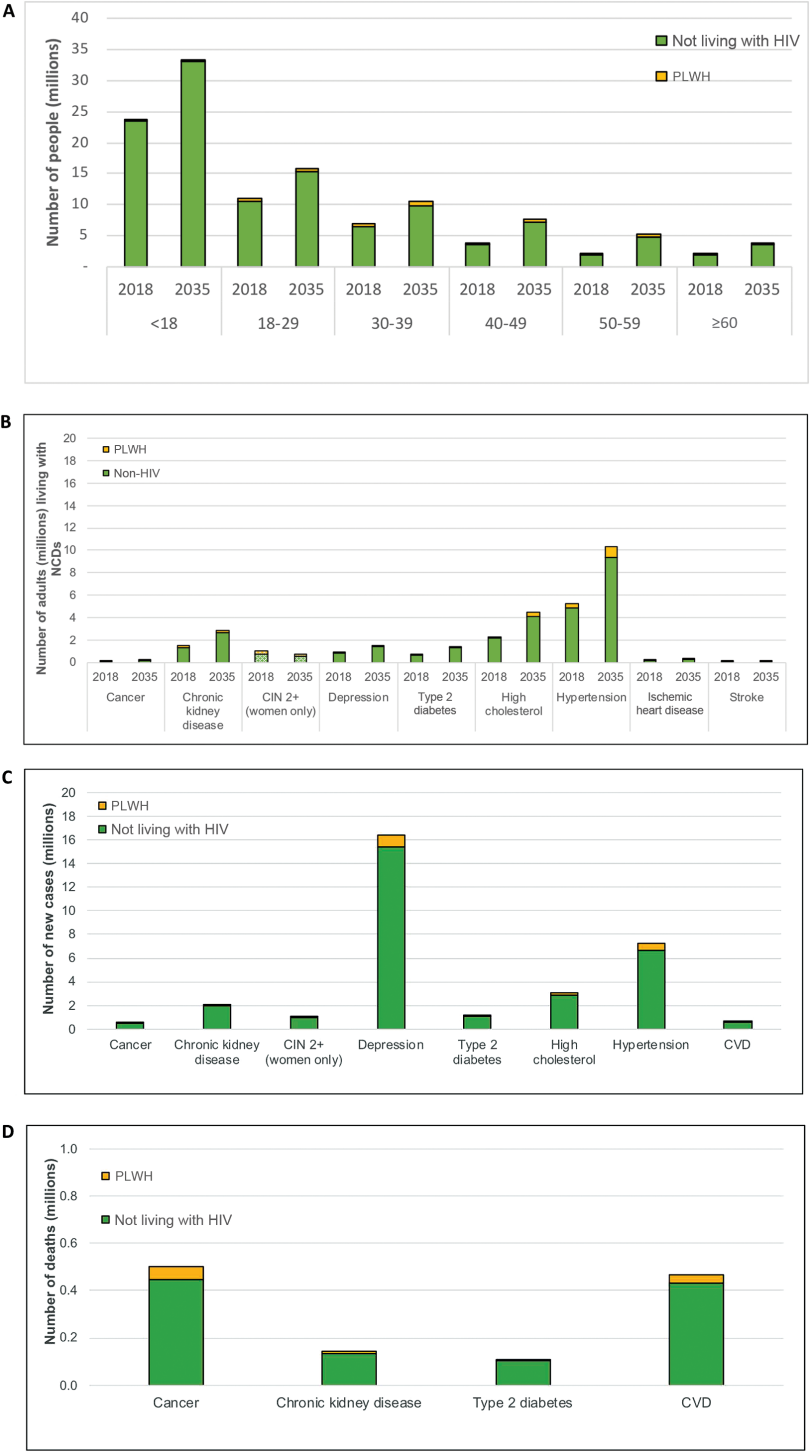
Estimates and projections of noncommunicable disease (NCD) burden by human immunodeficiency virus (HIV) status in Kenya. *A*, Number of people in 2018 and 2035 by age group and HIV status. *B*, Numbers living with NCDs in 2018 and 2035. *C*, Number of new cases between 2018 and 2035. *D*, Number of people dying of an NCD between 2018 and 2035. Depression is defined as having depressive episode in the past 12 months. Cardiovascular disease includes ischemic heart disease and ischemic stroke. Estimates of precancerous lesions of the cervix (cervical intraepithelial neoplasia 2+) are limited to women aged 15 years and older. Abbreviations: CVD, cardiovascular disease; CIN, cervical intraepithelial neoplasia; HIV, human immunodeficiency virus; NCD, noncommunicable disease; PLWH, people living with human immunodeficiency virus.

Hypertension and high total cholesterol are the main cause of NCD burden, with an adult prevalence of 20.5% (5.3 million people) and 9.0% (2.3 million people), respectively. Other CVD-related NCDs are also estimated to be high (CKD, 5.6% [1.4 million people] and diabetes, 2.7% [0.7 million]). CVD and cancers are the main causes of death in Kenya in 2018 (Figure 2D).

### Future Demographic and Epidemiological Estimates

The model estimates that the total population of Kenya will increase from 49.6 million in 2018 to 76.7 in 2035. Assuming HIV incidence rates remain unchanged and ART coverage increases steadily to reach the 90-90-90 goals by 2020, the population of PLWH will increase from 1.6 to 2.7 million people. Mean age is predicted to increase from 22 in 2018 to 24 in the general population and from 33 to 40 among PLWH between 2018 and 2035.

The NCD burden is forecasted to increase in the coming decades but remain consistently higher among PLWH; by 2035, 56% of Kenyan adults not living with HIV and 71% of adult PLWH will suffer from ≥1 NCD (Figure 2B). The model predicts that the NCD burden would remain substantial, even if pooled estimates overestimated individual NCD burden (10% reduction in age-specific NCD incidence would translate to 55% of HIV-negative and 69% of PLWH with ≥1 NCD in 2035). While the demographic shift will result in an increased NCD burden over time (irrespective of HIV status), population growth will result in a sharp increase in the absolute number of adults needing services for NCD. The prevalence of hypertension, for example, is expected to rise from 20% to 24% between 2018 and 2035, assuming current coverage of prevention and treatment services remains unchanged. However, the number of adults with hypertension is expected to increase from 5.3 million to 10.3 million, with 7.2 million new cases in this time period. As a result, the number of adult PLWH and people not living with HIV needing services for NCD will increase from 1.1 million and 14.3 in 2018 to 2.2 million and 25.9 million by 2035, respectively (Figure 2A).

The number of adults living with high total cholesterol, diabetes, and CKD are also expected to increase in this time period (Figure 2B), with 3.0 million, 1.1 million, and 2.1 million new cases, respectively, predicted between 2018 and 2035. As a result of these increases in CVD-related NCDs, CVD incidence is predicted to increase between 2018 and 2035. The model predicts that 32 000 adult PLWH and 444 100 of adults not living with HIV will experience a first CVD event in this period (Figure 2C), with a higher ASI among PLWH (822.7 per 100 000 in 2020–2025 vs 814.9 per 100 000 in 2030–2035) than adults not living with HIV (754.2 per 100 000 in 2020–2025 vs 759.7 per 100 000 in 2030–2035).

Depression is also an important contributor to NCD burden, with 16.4 million new cases of depression expected between 2018 and 2035 (Figure 2C). The prevalence of depression in 2018 is expected to remain stable at 3.4%, with a higher burden in adult PLWH compared to adults not living with HIV (3.9% vs 3.3%). This is mainly due to the increased risk for depression among PLWH.

With regards to cancers, the model estimates that crude incidence of cancers as a whole in the total population will increase steadily from 309.76 per 100 000 in PLWH and 47.71 per 1000 000 in adults not living with HIV in 2018 to 343.23 per 100 000 and 55.42 per 100 000 in 2035, respectively. The model estimates that a cumulative 0.55 million adults in Kenya will be diagnosed with any cancer between 2018 and 2035. Of those, 20.0% will be caused by cervical cancer, 18.0% by breast cancer, 11.6% by prostate cancer, 10.2% by esophageal cancer, 7.7% by colorectal cancer, 4.7% by stomach cancer, 3.7% by leukemia, 2.6% by liver cancer, and 21.2% by other cancers. Supplementary Tables 2.4–2.7 provide more detailed estimates of NCDs by HIV status and age from 2020 to 2035.

## DISCUSSION

Assuming current coverage of preventive and curative NCD service, the burden of NCDs in Kenya is set to increase in the coming 20 years, particularly in PLWH. The increase in NCD burden will be driven by population growth, and among PLWH largely by the rapidly aging population and, to a lesser extent, the cumulative exposure to HIV and ART. While NCD prevalence is predicted remain relatively stable, demographic growth will result in twice the number of adults needing services for NCDs in Kenya by 2035.

Although clear guidelines are already in place [1, 77], service coverage remains low. The high and accelerating burden of NCDs will put further pressure on the health system planning with failure to act undermining both public health and ART programs in the country. Time-updated estimates and projections such as those generated here, will be important in supporting these efforts. Routine collection of in-country NCD program data will be vital to support these efforts.

Policy makers in Kenya are calling for the integration of services for NCD into HIV platforms as a way to facilitate rapid scale-up of NCD services. HIV care has evolved into a chronic care model and has been successfully integrated with other services (eg, services for nutrition, tuberculosis, and maternal health) and could be leveraged to providing services for NCDs. However, questions remain on how to best operationalize integration and how these platforms, set up to deal with a low-prevalence disease such as HIV, could handle more prevalent conditions such as hypertension without decreasing the quality of care. For integration to work, it will need to be underpinned by context-specific evidence. Research needs to explore the aforementioned questions and explore operationalization of integration, identify cost-effective delivery models, and identify effective prevention campaigns. Spare capacity in HIV facilities, particularly given the move to differentiated care of stable, virologically suppressed PLWH, needs to be monitored [78] and pilot studies are needed, demonstrating whether integration of NCD services into HIV platforms is cost-saving.

Our findings among PLWH are similar to those reported in other settings. Modeling studies from Botswana, Italy, the Netherlands, the United States, and Zimbabwe all forecast a rapid aging of PLWH paralleled by a growing burden of NCDs [5, 7, 19, 79]. In Italy and the United States, an estimated 89% of PLWH will suffer from ≥1 NCD in 2035, compared to 59% in Zimbabwe and 62% in Kenya in 2035 [5, 7]. Despite a large overlap in the NCDs included in each of these study (hypertension, diabetes, CVD, CKD, cancers), they each include a different number and type of NCDs, making it hard to make direct comparisons.

This is the first study to combine all country-specific data on NCDs in a low- to middle-income HIV-endemic setting into a modeling framework and provide detailed country-level NCD estimates by HIV status, currently and in the coming decades. Similar studies have been limited by the lack of available robust in-country estimated of NCDs. By using an individual-based modeling approach, this study is able to account for key risk factors for NCDs, including age, pre-existing conditions, and infection with HIV.

A limitation arises from the NCD data availability in Kenya. Although the SR identified a wealth of data, the model still relies on data from neighboring Tanzania, and data from high-income setting. For example, there is a lack of data on the prevalence of risk factors for NCDs and prevalence of NCDs by HIV status in Kenya, with the model relying on data from high-income settings. Consequently, we were not able to check the model output on NCD burden by HIV status to Kenyan data, with the exception of prevalence of HPV and CIN2/3, which were compared to pooled estimates collated by the SR (Supplementary Data 2). The contribution of HIV to NCD development is a topic of ongoing research and this study incorporated data consistent with our best understanding of this field. More research is needed to understand if the contribution of HIV infection to the development of NCDs is consistent across settings. As new data become available, the model results can be updated.

In addition, the model also has to make simplified assumptions around survival rates. The model fits to GBD estimates, in the absence of better cause-specific mortality estimates from Kenya. The model also does not account for contribution of risk factors such as smoking, alcohol, or diet to the development of NCDs. Several studies have shown that PLWH may be at an increased risk of smoking and drinking alcohol [4], and it is widely agreed that these factors increase the risk for a number of NCDs. However, there is a lack of consensus on the relative contribution of lifestyle factors to NCD risk and how this may differ by HIV status, and consensus on how these may change in the coming 20 years in Kenya. As a result, while the model accounts for overall lifestyle risk for NCDs, it assumes that the effect of lifestyle factors is uniform across the population and constant over time. If lifestyle factors such as smoking and alcohol are restricted to a small proportion of the population in Kenya, the model results may be overestimating the number of people suffering from 1 or more NCDs.

Similarly, the model does not account for potential changes in healthcare access (other than 90-90-90 scale-up) or structural changes that may impact both NCD and HIV burden (eg, national prevention campaigns) and how these may reduce NCD burden. The data on NCD burden in Kenya, obtained through the SR, only provides data on diagnosed cases of NCDs. While the review focused on population-based studies, this and the fact that the model does not simulate all NCDs will result in the model underestimating the true burden of NCDs in Kenya. Finally, the model does not simulate communicable diseases and how these could impact the risk of NCDs (eg, infection with hepatitis and liver cancer).

In conclusion, Kenya is set to face growing NCD healthcare needs in the country. A rapidly growing population and continued HIV epidemic is expected to result in a growing NCD burden in the coming 2 decades, with the number of people needing services estimated to almost double. While guidelines in Kenya already support provision of NCD services, coverage remains low. As policy aims to use integration of NCD services in HIV platforms in Kenya to increase coverage of NCD services, more research will be needed to guide optimal approaches and planning.

## Supplementary Data

Supplementary materials are available at *Clinical Infectious Diseases* online. Consisting of data provided by the authors to benefit the reader, the posted materials are not copyedited and are the sole responsibility of the authors, so questions or comments should be addressed to the corresponding author.

ciz1103_suppl_Supplement_1

ciz1103_suppl_Supplement_2

## Notes

***Author contributions.*** M. S. formulated the research question, constructed the model, designed the model adaptation, interpreted the results, and wrote the first draft of the manuscript. P. P.-G. led the systematic review and meta-analysis, carried out the data analysis and model parameterization, developed the human papillomavirus natural history compartmental model, and ran the multimorbidity model results. R. C. assisted in the data analysis and model adaptation. K. K. M., J. K., and N. K. assisted on all aspects of the Kenyan adaptation including the scoping of data from Kenya and interpretation of the results within the Kenyan context. T. B. H. contributed to the formulation of the research question and the interpretation of the data. T. B. H. and M. S. secured funding for this study. All authors contributed to the re-drafting of the manuscript and in the process of approving the final draft.

***Acknowledgments.*** The authors thank the funders for supporting this work as well as all national stakeholders who have supported the work by giving guidance on scope, data, and interpretation. We acknowledge joint center funding from the UK Medical Research Council and Department for International Development.

***Disclaimer.*** The sponsor of the study had no role in study design, data collection, data analysis, data interpretation, or writing of the report. The corresponding author had full access to all the data in the study and had final responsibility for the decision to submit for publication.

***Financial support.*** This work was supported by the Civilian Research and Development Foundation Global (prime partner is US President’s Emergency Plan for AIDS Relief) (grant number OISE-9531011 to M. S., T. B. H., P. P.-G., K. K. M., and N. K.); the National Institutes of Health (NIH) (grant number 1R21AG053093-01 to M. S., T. B. H., P. P.-G., and R. C.); and the Medical Research Council (grant number MR/R015600/1 to M. S., T. B. H., P. P.-G., and R. C.).

***Potential conflicts of interest.*** M. S. reports personal fees from Gilead Sciences and Maple Consulting, outside the submitted work. T. H. reports grant support from Wellcome, NIH, Medical Research Council, the Global Challenges Research Fund, and the Bill & Melinda Gates Foundation (BMGF), and consulting fees from the Global Fund, BMGF, World Health Organization, and Pharos Health Advisors, outside the submitted work. All other authors report no potential conflicts. All authors have submitted the ICMJE Form for Disclosure of Potential Conflicts of Interest. Conflicts that the editors consider relevant to the content of the manuscript have been disclosed.
