# Supplementary material for: Mapping the Current and Future Noncommunicable Disease Burden in Kenya by Human Immunodeficiency Virus Status: A Modeling Study
Source: Clin Infect Dis. 2019 Nov 17;71(8):1864–73. doi: 10.1093/cid/ciz1103 (PMC8240998; doi:10.1093/cid/ciz1103)
Supplement: ciz1103_suppl_Supplement_1 [file ciz1103_suppl_supplement_1.docx]

**Supplement 1 – Systematic reviews and meta-analyses**

**A. Search Strategy and selection criteria**

Systematic reviews were carried out to collate available evidence on the overall and/or age-specific prevalence or incidence of cardiovascular disease (CVD; includes ischemic heart disease (IHD) and stroke), chronic kidney disease (CKD), depression, type 2 diabetes, high total cholesterol, hypertension, cervical human papillomavirus infection (HPV) and cervical intraepithelial neoplasia grade 2 and 3 (CIN 2/3). Standard Meta-analysis of Observational Studies in Epidemiology (MOOSE) and Preferred Reporting Items for Systematic reviews and meta-analysis (PRISMA) methodology for systematic reviews were followed.^1–3^ Two reviewers (PP – MD and MSc and CR - MSc) performed the searches, screened articles and extracted data into Microsoft Excel.

Full details of the selection criteria are presented in Table S1.1. Medline, and Embase were searched from inception to 30^th^ May 2018 to identify population-based or primary care-based epidemiological studies reporting either prevalence or incidence of individual NCDs amongst adults in Kenya. Searches were not restricted by language, or quality of study. Studies focusing on paediatric populations, pregnant women or studies using definitions for NCDs other than the standard Kenyan clinical definitions^4^ (Table S1.2) were excluded. Where partial or no evidence for a specific disease was available for Kenya, the systematic review was repeated for Tanzania, assuming prevalence and incidence data from this neighbouring country to be a good proxy of the true burden in Kenya. The country has a comparable demography, burden of disease and healthcare profile. Reference lists of included full-text articles were screened to identify additional studies and grey literature was searched for official reports from the Kenya National Bureau of Statistics, Ministry of Health, Ministry of Medical Services, National AIDS and STIs Control Programme, and National AIDS Control Council.^5–8^ Search terms used for Medline and Embase and description of the screening and selection process for individual NCDs of interest are presented in Figure S1.1 to Figure S1.7.

**Table S1.1. Inclusion and exclusion criteria for the systematic review.**

| **Inclusion criteria** | **Exclusion criteria** |
| --- | --- |
| - Adult population: ≥ 18 years for all NCDs, except HPV and CIN 2/3 (≥15 years) - Prevalence and/or incidence reported for one or more of the NCDs of interest - Type of study: cross-sectional, case-control, cohort, randomised controlled trial and systematic review and meta-analysis - Sample: population-based or primary healthcare setting based (i.e. general practice, antenatal care facility, chronic HIV care facility) - Location: Kenya; if none available, in decreasing order, Tanzania and then Europe - Language: all - Time: inception to May 30, 2018 - Other: reporting on methods for ascertaining biological measurements and diagnostic criteria. | - Paediatric population - Pregnant women only - Prevalence and/or incidence not reported - Secondary and/or congenital aetiologies of NCDs - Unreported diagnostic criteria or methods for ascertaining biological measurements - Type of study: expert reviews or policy reports - Sample: specialised setting based (i.e. hospital or other non-ambulatory setting or specialised clinic) sampling from populations with pre-existing diseases other than HIV |

**Table S1.2. Clinical definitions for NCDs.**

Source: Ministry of Public Health and Sanitation.^4^

| **NCD** | **Definition** |
| --- | --- |
| Cardiovascular disease | study-ascertained diagnosis (e.g. based on medical records or standardised acute diagnostic criteria) of ischaemic heart disease or ischaemic stroke; |
| Chronic Kidney Disease | an estimated glomerular filtration rate ≤60ml/min/1.73m2 body surface without evidence for acute kidney failure; |
| Depression | study-ascertained diagnosis based on medical records or standardised questionnaire (e.g. PHQ-9, CIS-R); |
| Diabetes, type 2 | fasting plasma glucose ≥7.0mmol/l (126mg/dl) or 2–h plasma glucose ≥11.1mmol/l (200mg/dl); |
| High total cholesterol | ≥5.19mmol/l (200mg/dl); |
| Hypertension | either the presence of pre-hypertension, at ≥130/80 and <140/90, or overt hypertension, at ≥140/90; |
| HPV | Study-ascertained diagnosis of HPV infection based on DNA detection methods in cervical swap or biopsy samples |
| CIN lesions | Study- ascertained diagnosis of CIN 2+ (i.e. CIN 2 to CIS) based on expert-assessed cytology and/or biopsy |

**B. Data extraction and data pooling**

Crude, age-specific and/or age-standardized prevalence and/ or incidence estimates were derived from the studies identified in the systematic review into Microsoft Excel. Risk of bias was assessed as per MOOSE and PRISMA, and data was only pooled where appropriate. Where studies did not report these, they were calculated based data reported by the studies. The following formulae were used:

$${Prevalence}_{a}= \frac{C_{a}}{N_{a}} x100$$

$ASP= \sum_{a=1}^{n} ({Prevalence}_{a}x P_{a}$)

${Incidence}_{a}= \frac{{NC}_{a}}{{RN}_{a}} x100$,000

$ASI= \sum_{a=1}^{n} ({Incidence}_{a}x P_{a}$)

where *Prevalence* stands for the percentage prevalence, *a* stands for individual age groups, *C* stands for the number of existing cases during a given time period, *N* stands for the total population in the same time period, ASP stands for age-standardized prevalence, *n* stands for the number of age groups, *P_a_* stands for the standard population fraction in age group *a* based on World Health Organisation standard population,^9^ *NC* stands for number of new cases in a given time period, *RN* stands for at risk population in a given time period, *ASI*  stands for age-standardized incidence.

Where the systematic review only found one study for the NCD of interest, the single estimate was used to establish occurrence of disease in Kenya. Where more than one study reported on prevalence of a selected NCD a meta-analysis was carried out in Microsoft Excel to pool the results, including crude and age-specific estimates of the NCD. If the systematic review found several publications based on the same study population and reported on the same NCD, only the most recent or comprehensive study was included. Random effects models were used to pool, as per meta-analysis guidance on choice of model for pooling, which recommend random effects where studies do not sample from the same population.^10^ To pool age-specific estimates and where studies reported estimates by different age groups (e.g. 20-30 in study one and 20 to 35 in study two), uniform risk within age groups was assumed. To pool results, standard error (SE) and 95% confidence intervals (95% CI) were calculated (where not reported in the study) to perform pooling. As multiple studies were only found for prevalence data (not incidence) this was only done for prevalence estimates. The following formulae were used:

$$SE= \frac{{Prevalence}_{a}}{\sqrt{({Prevalence}_{a}* N_{a})}}$$

$$95\% CI= {Prevalence}_{a} \pm1.96({Prevalence}_{a}*SE)$$

**C. Screening and selection process**

Search terms used for Medline and Embase and description of the screening and selection process for individual NCDs of interest are presented in Figure S1.1 to Figure S1.7.

| **Figure S1.1 Systematic review of prevalence of chronic kidney disease in Kenya and Tanzania. A.** Flow diagram of systematic review selection process and **B.** Search terms used.  *131 one studies were identified in the systematic review for Kenya and 68 in the one for Tanzania.  **One study enrolled from a hospital; one reported on knowledge and attitudes towards chronic kidney disease, not prevalence; one was a narrative review of literature from Sub-Saharan Africa; one reported on urinary abnormalities amongst HIV-positive people and did not follow diagnosis guidelines; one was a diagnostic study comparing equations for glomerular filtration rate; one sampled a biased population of HIV-positive people not-on-ART; one sampled a biased population of sero-discordant couples; one was a comparison of different equations for calculating the eGFR; and one sampled from a population with pre-existing diabetes. |
| --- |
| 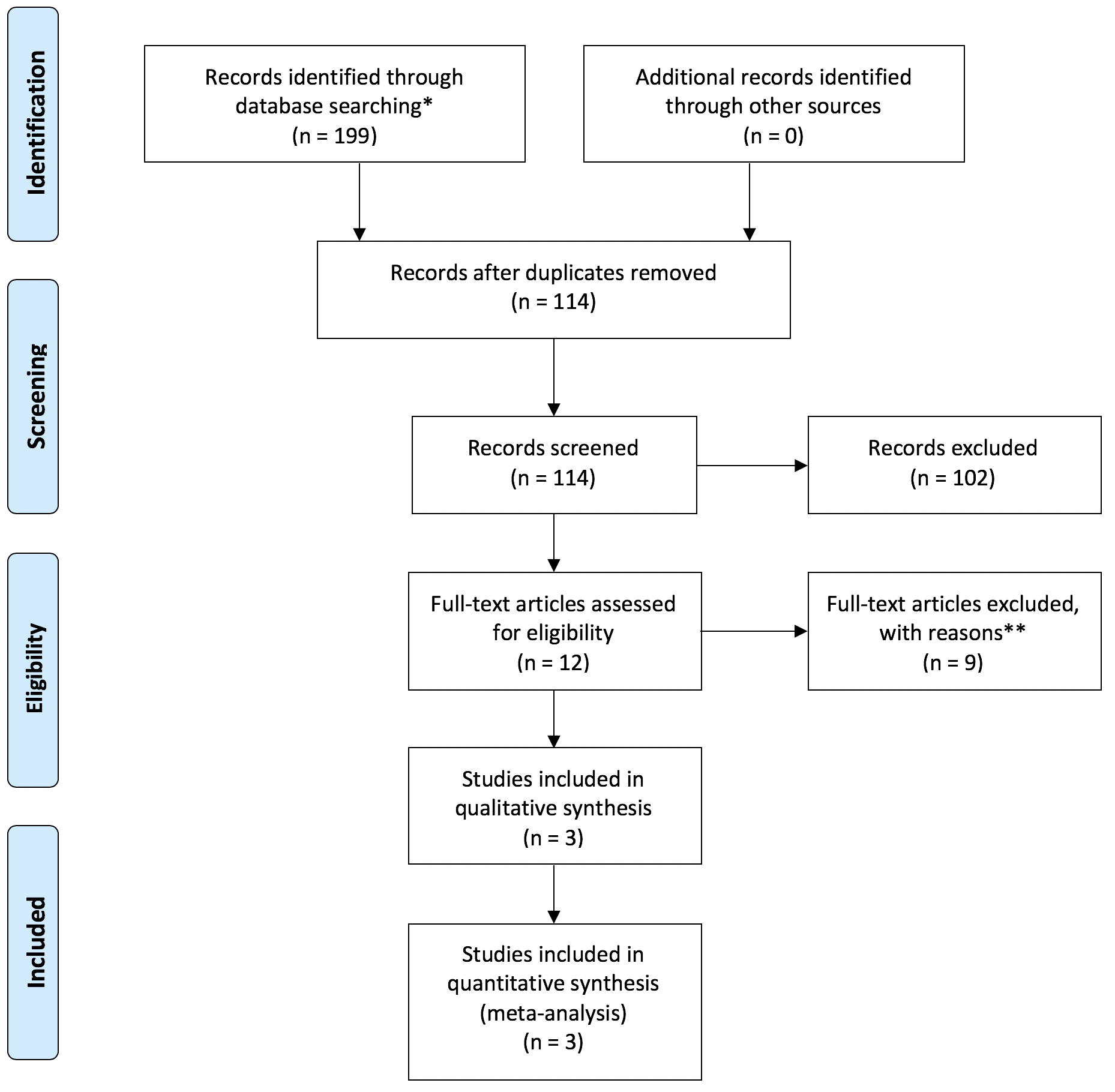 |
| **Medline filter:**  [chronic kidney disease.mp or Renal Insufficiency, Chronic/ or kidney failure.mp or Renal Insufficiency/] AND [Kenya.mp or KENYA/ or Tanzania.mp or Tanzania/] |
| **Embase filter:**  [chronic kidney disease.mp or chronic kidney failure/ or kidney failure.mp or kidney failure/ or renal insufficiency.mp] AND [Kenya.mp or KENYA/ or Tanzania.mp or Tanzania/] |

| **Figure S1.2. Systematic review of prevalence of depression in Kenya. A.** Flow diagram of systematic review selection process and **B.** Search terms used.  *Four additional articles were identified through cross-checking articles’ reference lists.  **Three studies screened for symptoms of depression or ‘sadness’, without corroborating diagnosis against standardised criteria; three utilised depression screening tools, without corroborating diagnosis against standardised criteria; three could not be found; two were hospital-based; two recruited a (biased) consecutive sample of PLHIV attending a clinic; one recruited a biased population of food-industry workers undergoing screening for infectious diseases; one was a review of the literature; one reported on a sub-sample of patients from another (included) study; and one reported association between depression and ART initiation, rather than prevalence, among a biased population of serodiscordant couples. | |
| --- | --- |
|  | |
| **Medline filter:**  [depression.mp or depression/ or depressive disorder.mp or depressive disorder/ or major depression.mp or depressive disorder, major/ or dysthymia.mp or dysthymic disorder] AND [Kenya.mp or KENYA/] | |
| **Embase filter:**  [geriatric depression scale/ or minor depression/ or depression/ or major depression/ or depression.mp. or Depression Anxiety Stress Scale/ or adolescent depression/ or long term depression/ or depression assessment/ or depressive disoder.mp or major depression.mp or dysthymia.mp or dysthymia/] AND [Kenya.mp or KENYA/] | |
| **Figure S1.3 Systematic review of prevalence of type 2 diabetes in Kenya. A.** Flow diagram of systematic review selection process and **B.** Search terms used.  *An additional study was found from the systematic search for chronic kidney disease [1] and another from the Kenya Ministry of Health [2].  **Four studies were not primary-healthcare based; four were reviews of literature from Sub-Saharan Africa; two had a study designs other than observational; five reported on subsequent analyses of previous studies; one reported on prevalence of gestational diabetes; one was amongst hypertensive patients; two had no information on sampling methods; three used diagnostic criteria other than standard guidelines; and one study could not be retrieved for full-text assessment. |  |
| 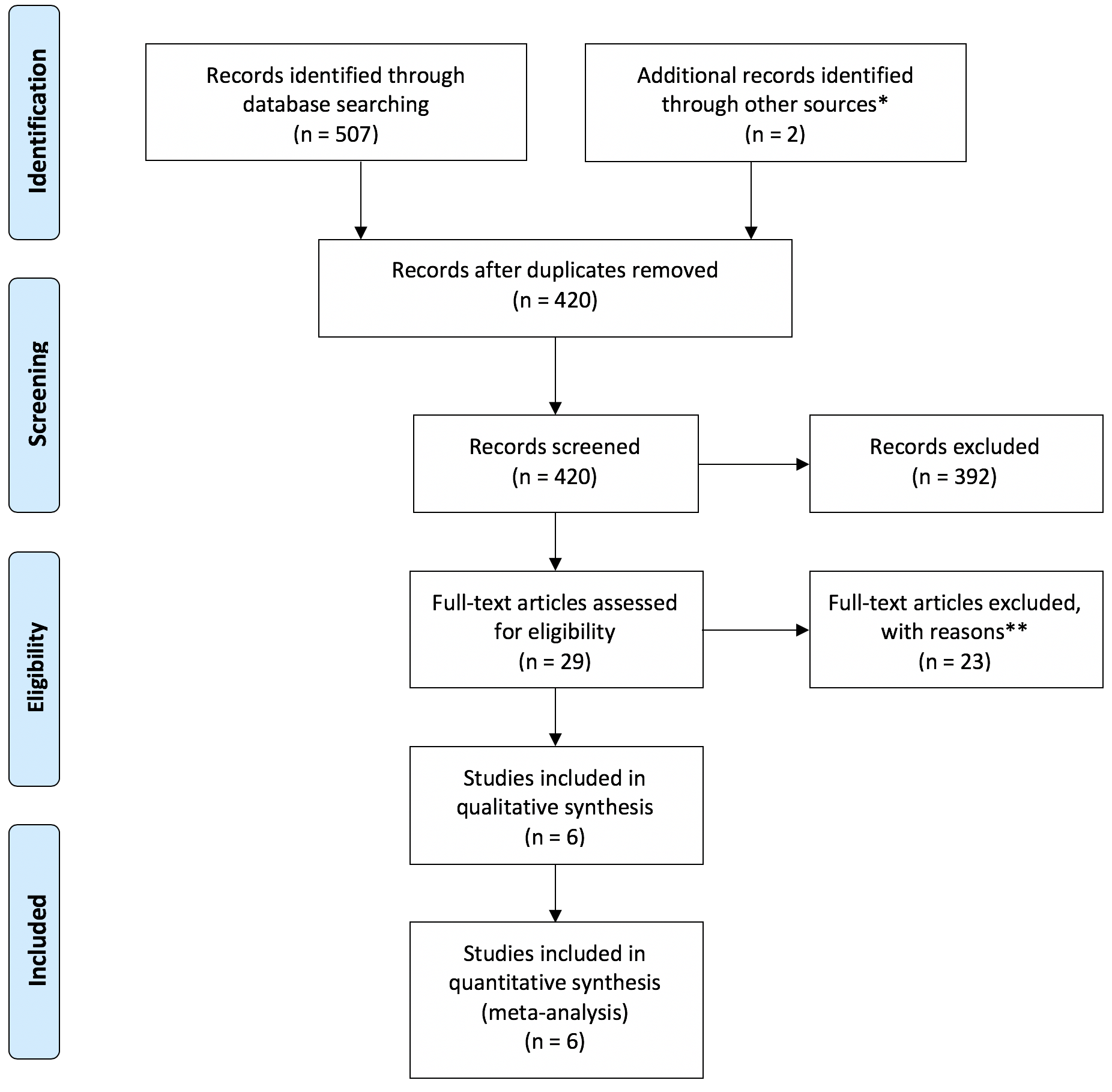 |  |
| **Medline filter:**  [Blood glucose/ or Diabetes Mellitus, Type 2/ or DIABETES MELLITUS/ or Diabetes.mp or type 2 diabetes.mp] AND [Kenya.mp or KENYA/] |  |
| **Embase filter:**  [diabetes.mp or diabetes mellitus/ or type 2 diabetes.mp or non insulin dependent diabetes mellitus/ or blood glucose.mp or glucose blood level/] AND [Kenya.mp or KENYA/] |  |

| **Figure S1.4 Systematic review of the prevalence of high total cholesterol in Kenya. A.** Flow diagram of systematic review selection process and **B.** Search terms used.  *An additional study was found from the Kenya Ministry of Health [2].  **Three studies provided estimates for cholesterol measures other than total serum cholesterol; one study reported on therapeutic interventions; one study reported overall prevalence in people over 50 years old only; and one full-text study could not be retrieved. |
| --- |
| 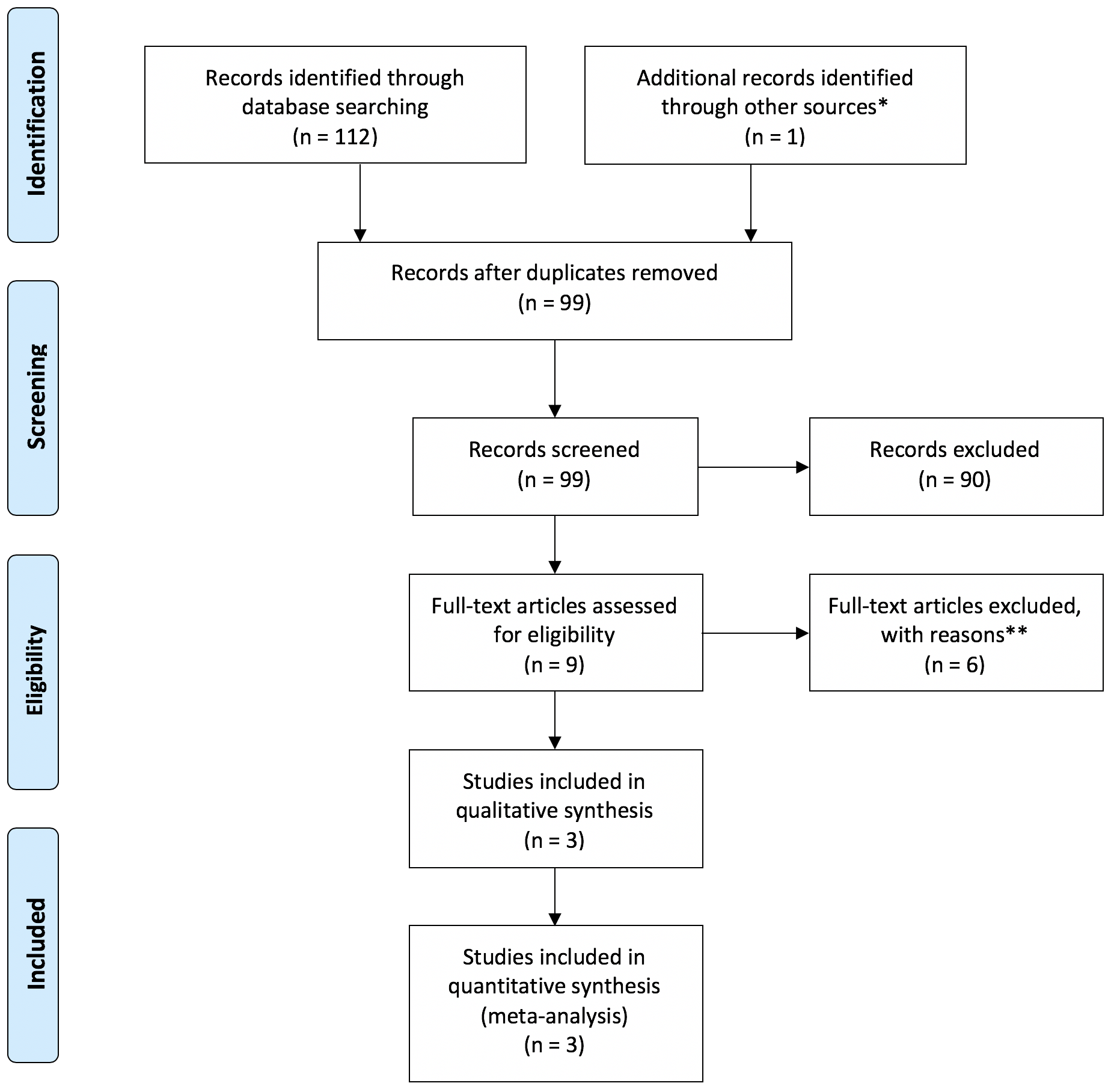 |
| **Medline filter:**  [dyslipidemia.mp or dyslipidemia/ or high cholesterol.mp or high cholesterol/ or high blood cholesterol.mp or high blood cholesterol/ or lipid disorder.mp or lipid disorder/ or hyperlipidemia.mp or hyperlipidemia/ or hypercholesterolemia.mp or hypercholesterolemia/] AND [Kenya.mp or KENYA/] |
| **Embase filter:**  [dyslipidemia.mp or dyslipidemia/ or high cholesterol.mp or high cholesterol/ or high blood cholesterol.mp or high blood cholesterol/ or lipid disorder.mp or lipid disorder/ or hyperlipidemia.mp or hyperlipidemia/ or hypercholesterolemia.mp or hypercholesterolemia/] AND [Kenya.mp or KENYA/] |

| **Figure S1.5 Systematic review of prevalence of hypertension in Kenya. A.** Flow diagram of systematic review selection process and **B.** Search terms used.  *An additional study was found from the Kenya Ministry of Health [2].  **Eleven studies were intervention studies; nine could not be retrieved for full-text; six reported the wrong outcome measure; four were in populations who were already hypertensive; three were qualitative studies; three were follow-up publications from previous studies; two studies reported on hypertension awareness and risk factors; one reported prevalence for the African region not for Kenya individually; one samples post-renal transplant patients; one was a health economic analysis; one ascertained prevalence solely based on self-reported previous diagnosis. |
| --- |
| 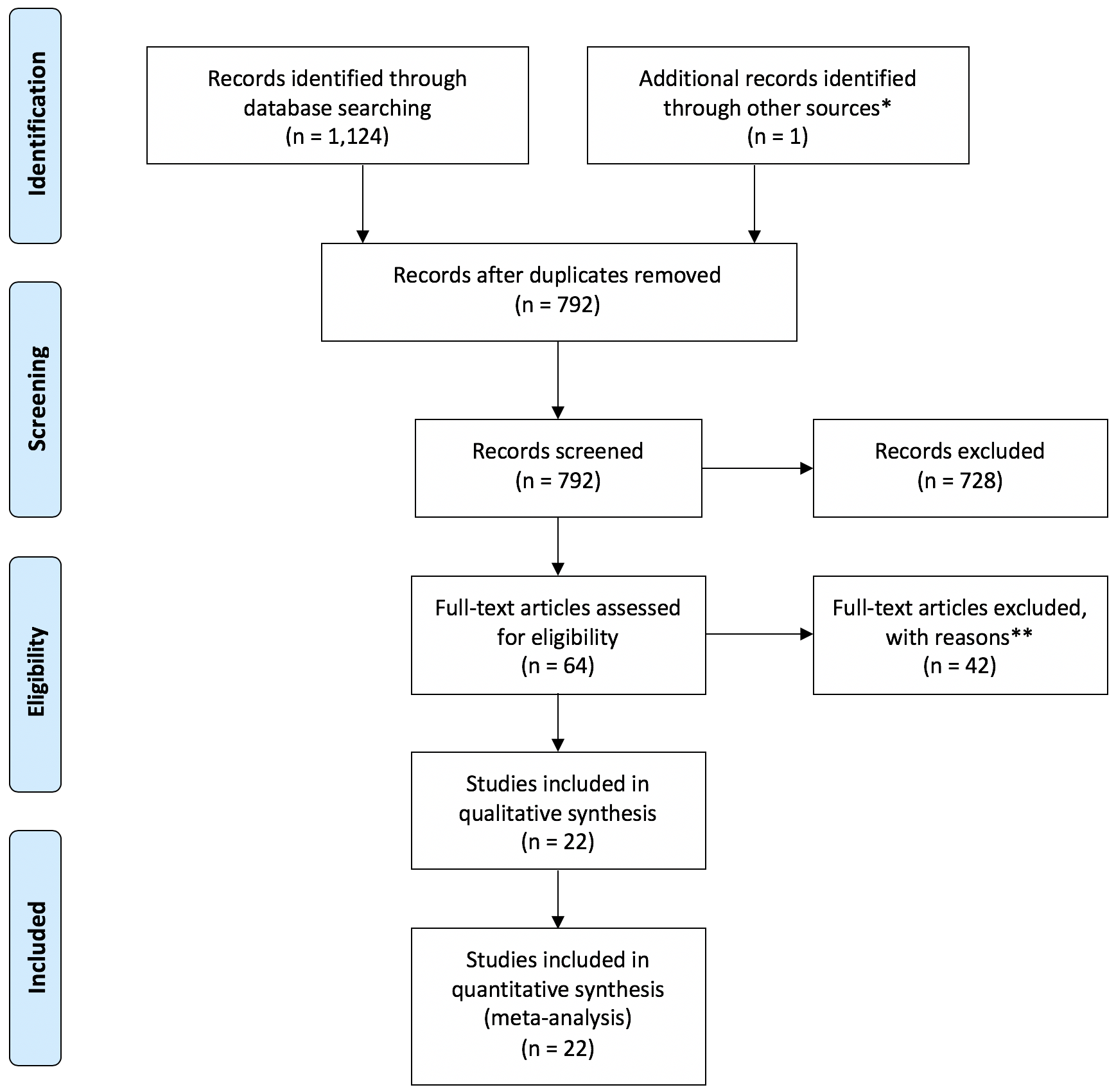 |
| **Medline filter:**  [hypertension.mp or hypertension/ or high blood pressure.mp] AND [Kenya.mp or KENYA/] |
| **Embase filter:**  [hypertension.mp or hypertension/ or high blood pressure.mp] AND [Kenya.mp or Kenya/] |

| **Figure S1.6 Systematic review of incidence of ischaemic heart disease in Kenya and Tanzania. A.** Flow diagram of systematic review selection process and **B.** Search terms used.  *150 studies were identified in the systematic review for Kenya and 109 in the one for Tanzania.  **Six studies could not be found for full text screening; two were verbal autopsy studies; one reported on history of stroke, hypertensive heart disease and peripheral artery disease among know hypertensives and diabetics; one paper reported the number of myocardial infarction cases in a hospital setting; one reported fatal cases of myocardial infarction; and one study reported on the prevalence of electrocardiographic abnormalities consistent with previous myocardial infarction among people with previous stroke and matching controls. |
| --- |
| 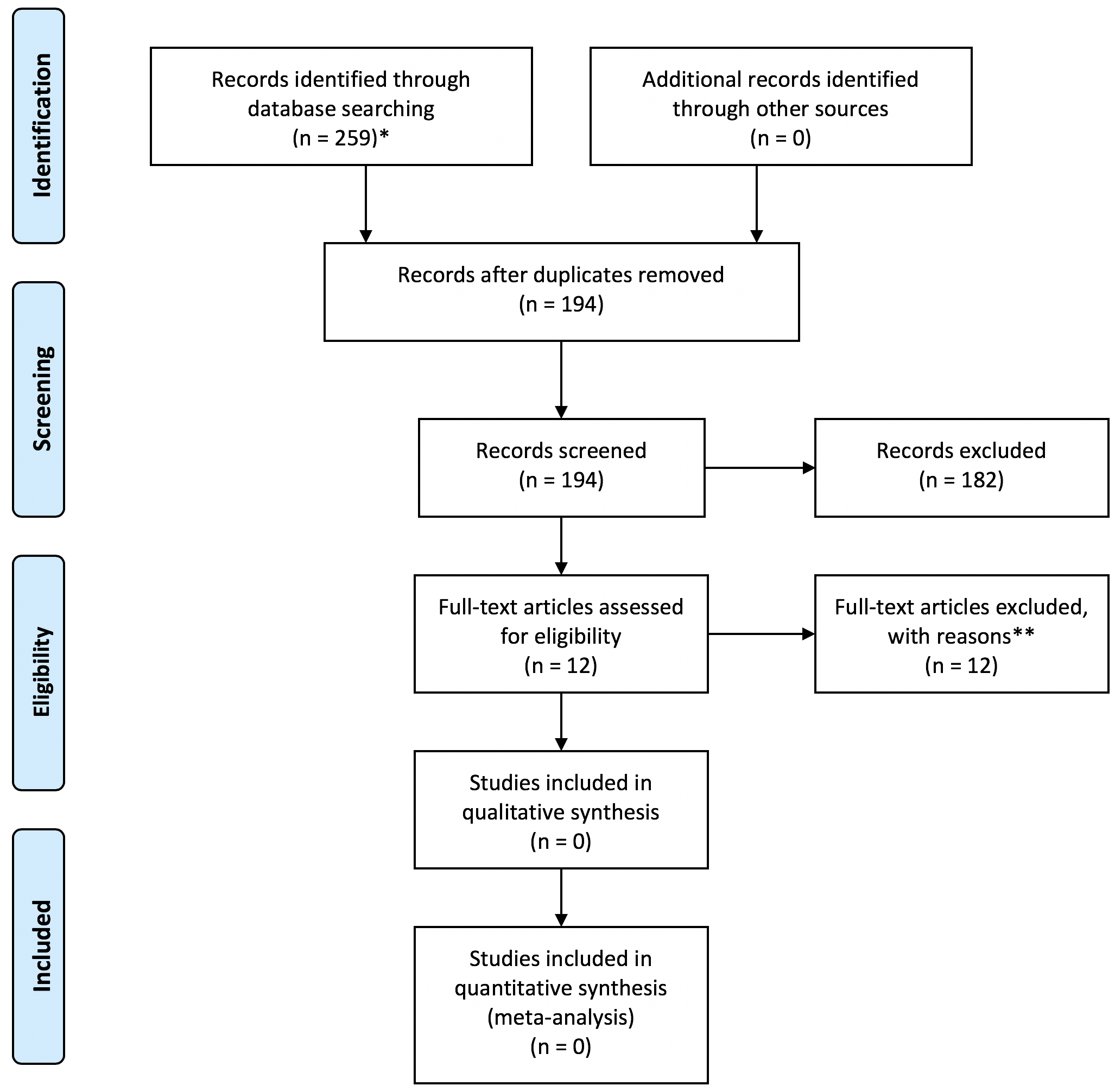 |
| **Medline filter:**  [myocardial infraction.mp or myocardial infraction/ or cardiovascular diseases/ or myocardial ischemia/ or heart infarction.mp or coronary disease/ or MI.mp or myocardial ischemia/ or coronary artery disease/ or acute heart infarction.mp or heart attack.mp or angina.mp or angina pectoris, variant/ or angina pectoris/ or angina, unstable/ or angina, stable/ or angina pectoris.mp or stable angina.mp or unstable angina.mp or ST elevation myocardial infarction/ or ST elevation.mp or non st elevation.mp or acute coronary syndrome.mp or coronary thrombosis/ or ischemic heart disease.mp or ischaemic heart disease.mp] AND [Kenya.mp or KENYA/ or Tanzania.mp or TANZANIA/] |
| **Embase filter:**  [myocardial infraction.mp or heart infraction/ or heart attack.mp or acute heart infarction/ or MI.mp or acute heart infarction.mp or angina.mp or angina pectoris/ or angina pectoris.mp or unstable angina.mp or unstable angina pectoris/ or stable angina.mp or stable angina pectoris/ or ST elevation.mp or ST segment elevation/ or acute coronary syndrome/ or non ST segment elevation myocardial infarction/ or heart muscle ischemia/ or non st elevation.mp or coronary artery disease/ or ischemic heart disease/ or coronary artery thrombosis/ pr ischemic heart disease.mp or ischaemic heart disease.mp] AND [Kenya.mp or KENYA/ or Tanzania.mp or TANZANIA/] |

| **Figure S1.7 Systematic review of incidence of ischemic stroke in Kenya and Tanzania. A.** Flow diagram of systematic review selection process and **B.** Search terms used.  *119 studies were identified in the systematic review for Kenya and 144 in the one for Tanzania  **No study from Kenya (n=2) assessed in full text, met inclusion criteria as they were both hospital-based retrospective studies. From Tanzania, four were expert review articles; three studies reported mortality rates; two full-text articles could not be retrieved; two reported on other outcomes of interest not related to incidence or prevalence; one reported prevalence of several neurological disorders based solely on a questionnaire; one was a previous systematic review and meta-analysis of the prevalence and incidence of stroke pooling all available studies from Sub-Saharan Africa. |
| --- |
| 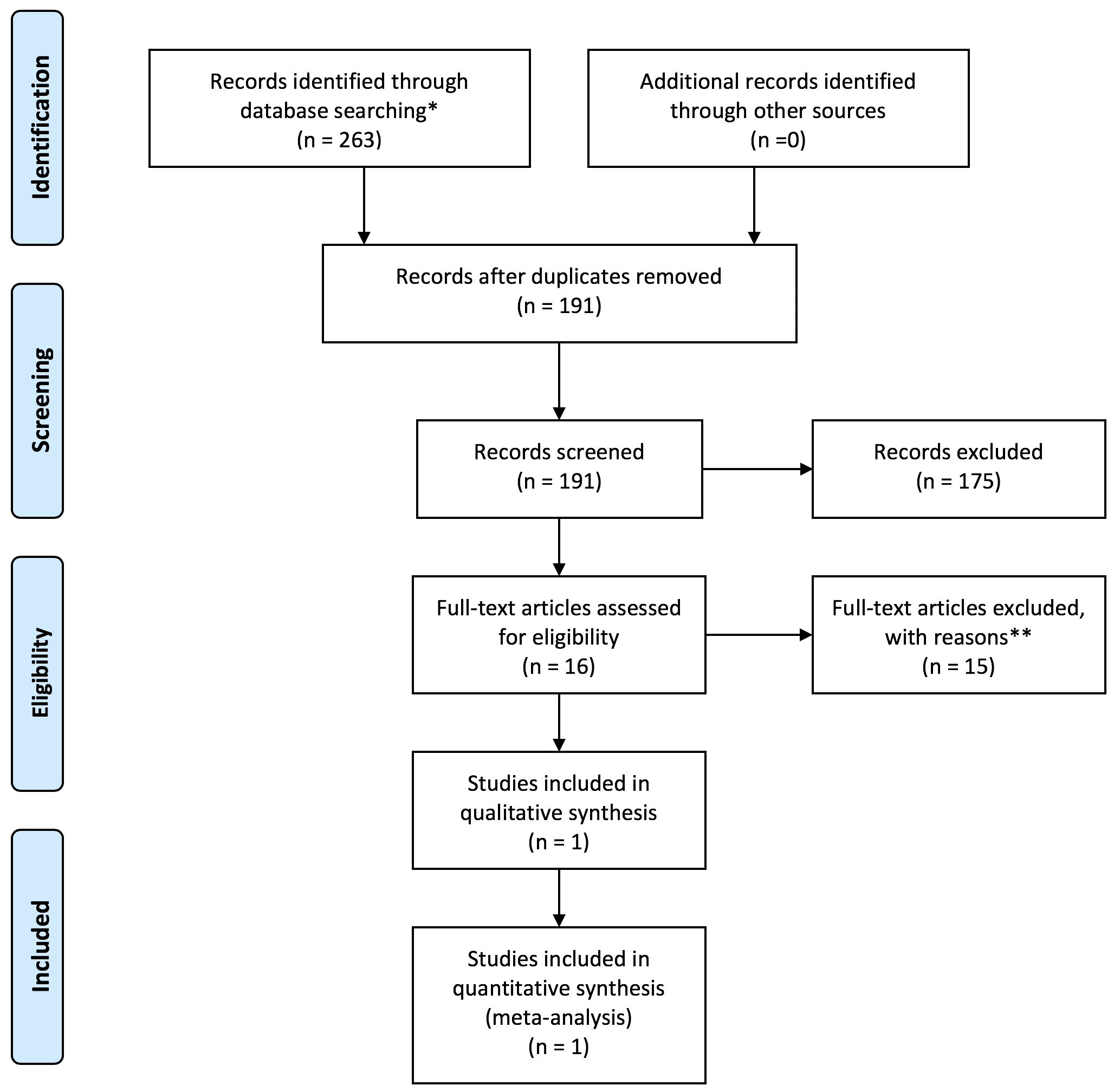 |
| **Medline filter:**  [stroke.mp or stroke/ or stroke, lacunar/ or “National Institute of Neurological Disorders and Stroke (U.S.)”/ or cerebrovascular accident.mp or cerebrovascular disorders/ or brain ischemia/ or brain ischemia.mp or ischaemic stroke.mp or ischemic attack, transient/ or ischemic stroke.mp or infarction, middle cerebral artery/] AND [Kenya.mp or KENYA/ or Tanzania.mp or Tanzania/] |
| **Embase filter:**  [stroke.mp or cerebrovascular accident/ or cerebrovascular accident.mp or ischemic stroke.mp or brain ischemia/ or ischaemic stroke.mp or brains ischemia.mp] AND [Kenya.mp or KENYA/ Tanzania.mp or Tanzania/] |

| **Figure S1.8 Systematic review of prevalence of HPV infection of the cervix and CIN lesions in Kenya. A.** Flow diagram of systematic review selection process and **B.** Search terms used.  *An additional study was found from the Catalan Information Centre on HPV and cancer and another from personal communication with Chung et al. at the Coptic Hope Centre – University of Washington.  **Six were diagnostic tests studies comparing different cervical cancer screening methods; six were previous systematic reviews and meta-analyses, with different outcome measures to those of interest, or expert reviews; six reported on HPV prevalence in sub-samples of the population which were not representative of the general population (i.e. four recruited female sex-workers and two women with cervical cancer); one reported clustering patterns of HPV genotypes among HIV-positive women; one reported prevalence of residual HPV infection after cryotherapy; and one reported on genotypes frequency among HIV-positive women with bacterial vaginosis. |
| --- |
|  |
| **Medline filter:**  [tumor virus infections/ or cervical intraepithelial neoplasia/ or uterine cervical neoplasms/ or carcinoma, squamous cell/ or papillomaviridae/ or papillomavirus infections/ or human papillomavirus.mp or papillomavirus vaccines/ or hpv.mp or DNA probes, HPV/ or alphapapillomavirus/] AND [Kenya.mp or KENYA/] |
| **Embase filter:**  [wart virus/ or hpv.mp or human papillomavirus.mp] AND [Kenya.mp or KENYA/] |

**D. Results of the systematic review and meta-analysis**

**Cardiovascular disease**

For CVD, the focus was on studies reporting on either ischaemic heart disease (IHD) or stroke incidence in Kenya. The systematic review for ischaemic stroke yielded 119 studies from Kenya, none of which met inclusion criteria. The systematic review was repeated for Tanzania, where 144 studies were identified, one met inclusion criteria (Figure S1.7 and Table S1.3).^11^

The study reported stroke incidence in urban and rural Tanzania between 2003 and 2006. In order to get an overall prevalence, we pooled these estimates, weighting for relative population sizes. The resulting crude incidence was 83.9 per 1000,000 person-years (95% CI 67.7 to 101.9 per 100,000) and ASI was 114.8 per 100,000 (95% CI 102.7 to 129.4 per 100,000). The age-specific incidence is shown in Figure S1.8.

The systematic review for IHD identified 150 studies for Kenya, none of which met the inclusion criteria (Figure S1.6). The search was repeated for Tanzania, where 109 studies were identified (Figure S1.6), with no studies meeting the inclusion criteria.

**Table S1.3. Study details and outcomes available for cardiovascular disease.**

*Abbreviations: Disag; dissagragated; LB, lower bound of 95% confidence interval; UB, upper bound of 95% confidence interval.*

| Study | Study period | Sample size | Country | Study Setting | Study design | Disag. by HIV | Disag. by age | Definition | Crude Incidence (LB-UB) |
| --- | --- | --- | --- | --- | --- | --- | --- | --- | --- |
| Walker et al. 2010 | June 2003 to June 206 | 216,331 | Tanzania | Community and health facility | Survey and hospital data analysis | No | Yes | Clinic reports and/ or autopsy | 83.9 per 100,000 (67.7 to 101.9 per 100,000) |

**Figure S1.8.** **Age-specific stroke incidence per 1000,000 between 2003 to 2006.**^11^ On far right the crude (blue) and age-standardized (orange) prevalence (ASP) using standard direct method and WHO standard population.

**Chronic kidney disease**

The systematic review on CKD yielded 131 studies from Kenya. After removing duplicates, 72 abstracts and nine full-text articles were assessed for eligibility. One study reported overall prevalence of CKD, although it did not report prevalence disaggregated by age.^12^ Therefore, the search was expanded to Tanzania, where a further 68 studies were identified, of which two met inclusion criteria (Figure S1.1 and Table S1.4).^13,14^

Crude prevalence from all three (i.e. the one Kenyan and the two Tanzanian) studies was estimated to be 10.1% (95% CI 6.2 to 14.0) (Figure S1.9A). Prevalence for those aged 18-29, 30-39, 40-49, and 50-59 was 5.9% (95% CI 0.7-11.2), 6.2% (95% CI 3.5-8.9), 10.4% (95% CI 6.5-14.3), 12.4% (95% CI 8.1-16.6), respectively (Figure S1.9B***–*** *blue solid line*). Only one study reported estimated amongst people aged ≥60, at 14.4% (95% CI 7.6-21.3) (Figure S1.9B ***–*** *purple dotted line*).^13^ The resulting ASP was 9.2% (95% CI 4.6 to 13.8) (Figure S1.9B).

**Table S1.4. Study details and outcomes available for CKD.**

**eGFR was calculated by a number of equations across studies. When an individual study quantified multiple equations, priority was given (in decreasing order) to CKD-EPI, MDRD and CG.*

*Abbreviations: CG, Cockcroft-Gault; CKD-EPI, chronic kidney disease epidemiology collaboration; Disag., disaggregated; eGFR, estimated glomerular filtration rate; LB, lower bound of 95% confidence interval; UB, upper bound of 95% confidence interval; MDRD, modification of diet in renal disease study.*

| Study | Study period | Sample size | Country | Study Setting | Study design | Disag. by HIV | Disag. by age | Definition* | Crude prevalence (LB-UB) |
| --- | --- | --- | --- | --- | --- | --- | --- | --- | --- |
| Edwards et al. 2015 | January to June 2013 | 1,800 | Kenya | Primary clinic-based | Retrospective analysis of clinical records | Yes | No | eGFR <60mL/min | 11.6%  (10.0-13.1) |
| Peck et al. 2016 | May to April 2013 | 1,043 | Tanzania | Population-based | Cross-sectional | Yes | Yes | eGFR <60mL/min | 6.6%  (5.1-8.2) |
| Stanifer et al. 2015 | January to June 2014 | 481 | Tanzania | Population-based | Cross-sectional | Yes | Yes | eGFR <60mL/min | 11.9%  (8.8-14.9) |

1. **
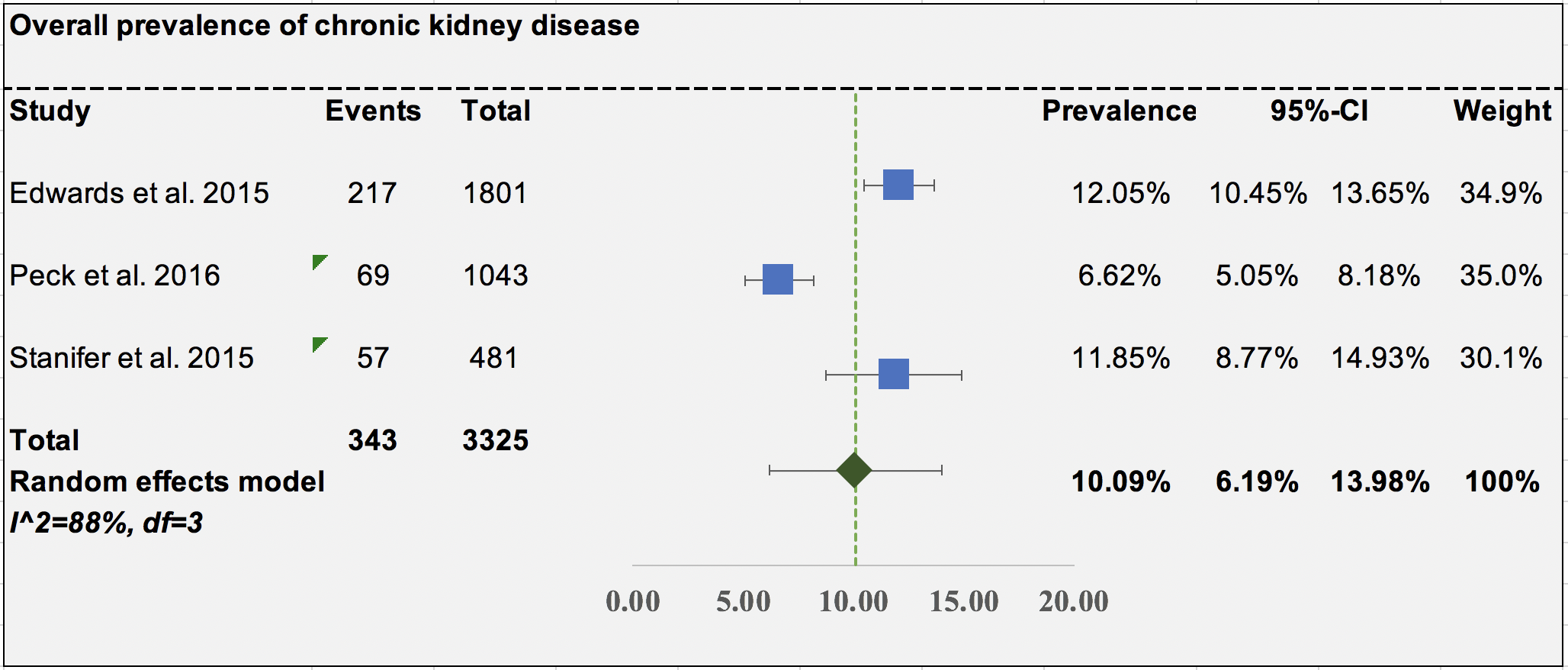
**
2.

**Figure S1.9. Prevalence of chronic kidney disease in Kenya.** A) Meta-analysis of the crude prevalence from three studies; B) Age-specific prevalence from two studies (dotted lines) and from meta-analysis using random effects model (solid blue line with 95% CIs), and on far right the calculated crude (blue) and age-standardized (orange) prevalence (ASP) from the meta-analysis. ASP used standard direct method and WHO standard population.

**Depression**

The systematic review yielded a total of 402 individual studies from indexed databases and four from grey literature. After removing duplicates, 264 abstracts and 26 full-text articles were assessed for eligibility (Figure S1.2). Nine studies reported specifically on depression (Figure S1.2 and Table S1.5),^15–24^ with the rest reporting on other mental health conditions (e.g. anxiety, manic disorders, and schizophrenia) or using diagnostic tools others than those listed in Table S1.2. Eight studies reported on recent/current depression (hereon forth referred to as ‘depression’), one on lifetime depression and one on both depression and lifetime depression (Table S1.5).

The crude prevalence of active or recent episodes of depression across nine studies was calculated as 8.5% (95% CI 5.1 to 11.8%) and of lifetime prevalence across two studies as 12.2% (95% CI 0.9% to 24.5%) among Kenyan adults (Figure S1.10A and B). The resulting ASP was 6.3% (95% CI 4.3 to 8.5). Only one study reported prevalence of depression by age, as shown in Figure S1.10C, using the World Mental Health Survey version of the Composite International Diagnostic Interview (WHM-CIDI) to ascertain episodes of major depression in the 12 months previous to the application of the interview.^16^

**Table S1.5. Study details and outcomes available for depression.**

**Only those studies that used interview instruments adhering to standardised diagnostic criteria from the Diagnostic and Statistical Manual of Mental Disorders, whichever version was current at the time of the survey (i.e. III to V) where included in the synthesis.*

*Abbreviations: BDI, Beck Depression Inventory; CIDI, Composite International Diagnostic Interview; CIS-R, Clinical Interview Schedule-Revised; Disag., disaggregated; LB, lower bound of 95% confidence interval; UB, upper bound of 95% confidence interval; MDI, Major Depression Inventory; MINI, Mini-plus International Neuropsychiatric Interview; PHQ-9, 9-item Patient Health Questionnaire; WHM-CIDI, World Mental Health Survey version of the Composite International Diagnostic Interview.*

| Study | Time horizon | Study period | Sample size | Country | Study Setting | Study design | Disag. by HIV | Disag. by age | Diagnostic criteria* | Crude prevalence (LB-UB) |
| --- | --- | --- | --- | --- | --- | --- | --- | --- | --- | --- |
| Aillon et al. 2014 | Last two weeks | 2010 | 300 | Kenya | Primary clinic-based | Cross-sectional | No | No | MINI Plus | 26.3%  (20.5-32.1) |
| Ambugo 2014 | Last 12 months | 2002 to 2004 | 4,331 | Kenya, Norway, UK and Ghana | Community-based | Cross-sectional | No | Yes | WMH-CIDI | 9.0%  (8.1-9.9) |
| Jenkins et al. 2012 | Last week | 2004 | 876 | Kenya | Community-based | Cross-sectional | No | No | CIS-R | 1.0%  (0.4-1.7) |
| Jenkins et al. 2015 | Last week | 2013 | 1,157 | Kenya | Community-based | Cross-sectional | No | No | CIS-R | 0.9%  (0.3-1.4) |
| Kwobah et al. 2017 | Last week | January 2014 to November 2015 | 420 | Kenya | Community-based | Cross-sectional | No | No | MINI Plus | 12.6%  (9.2-16.0) |
| Maj et. al. 1994 | Last month | October 1990 to August 1991 | 207 | Kenya, Germany, Brazil, Zaire and Thailand | Primary clinic-based | Cross-sectional | Yes | No | CIDI | 2.9%  (0.6-5.2) |
|  | Lifetime |  |  |  |  |  |  |  |  | 6.8%  (3.2-10.3) |
| Ndetei et al. 2009 | Last week | November 2005 to | 66 | Kenya | Primary to tertiary clinic-based | Cross-sectional | No | No | BDI | 22.7%  (11.2-34.2) |
| Nyongesa et al. 2018 | Last two weeks | November 2016 to March 2017 | 167 | Kenya | Primary clinic-based (HIV+) and community-based (HIV-negative) | Cross-sectional | Yes | No | MDI | 6.6%  (2.7-10.5) |
| Ongeri et al. 2018 | Lifetime | April to September 2015 | 394 | Kenya | Primary clinic-based | Cross-sectional | No | No | MINI Plus | 18.8%  (14.5-23.1) |

1. **
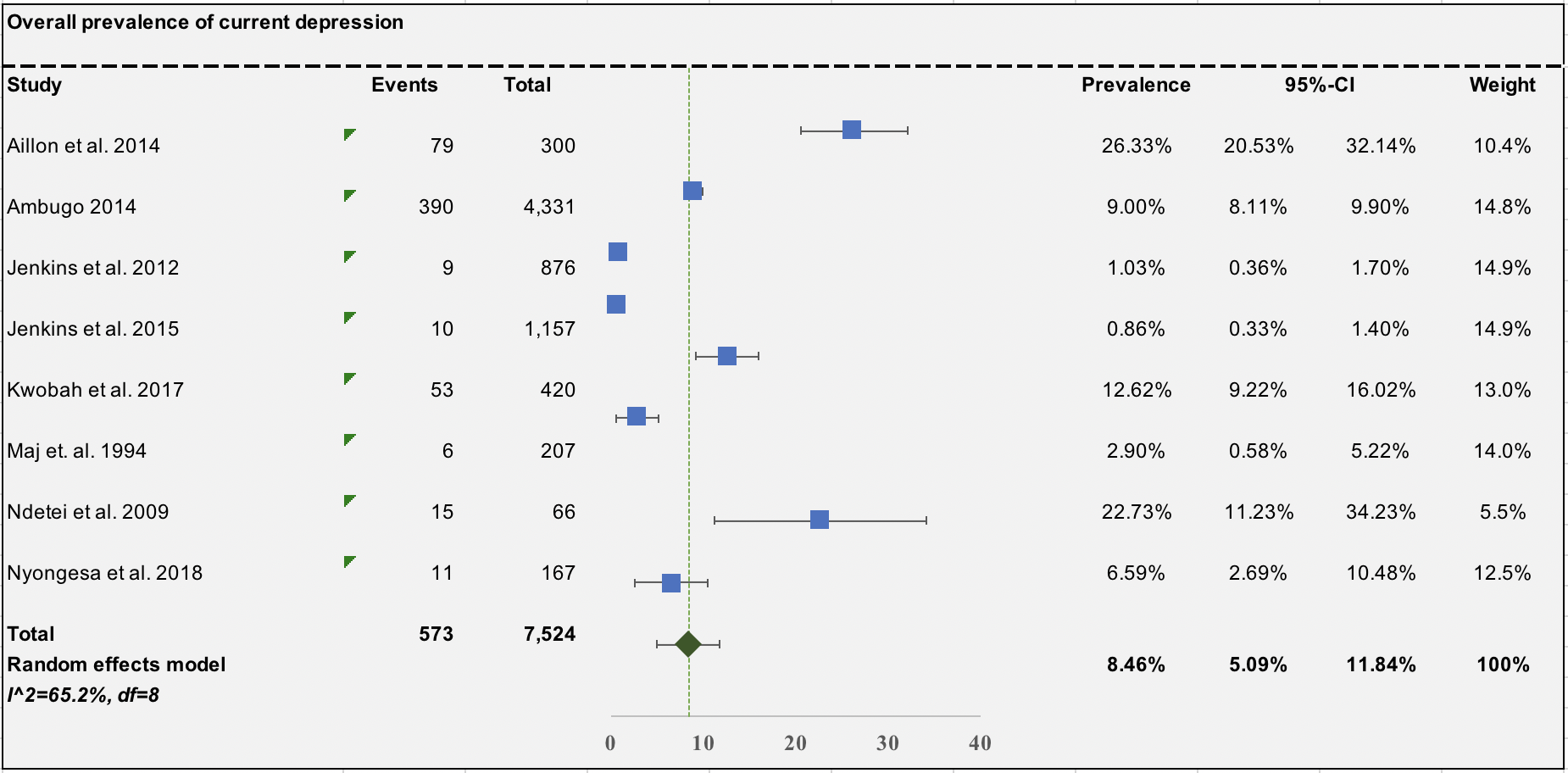
**
2. **
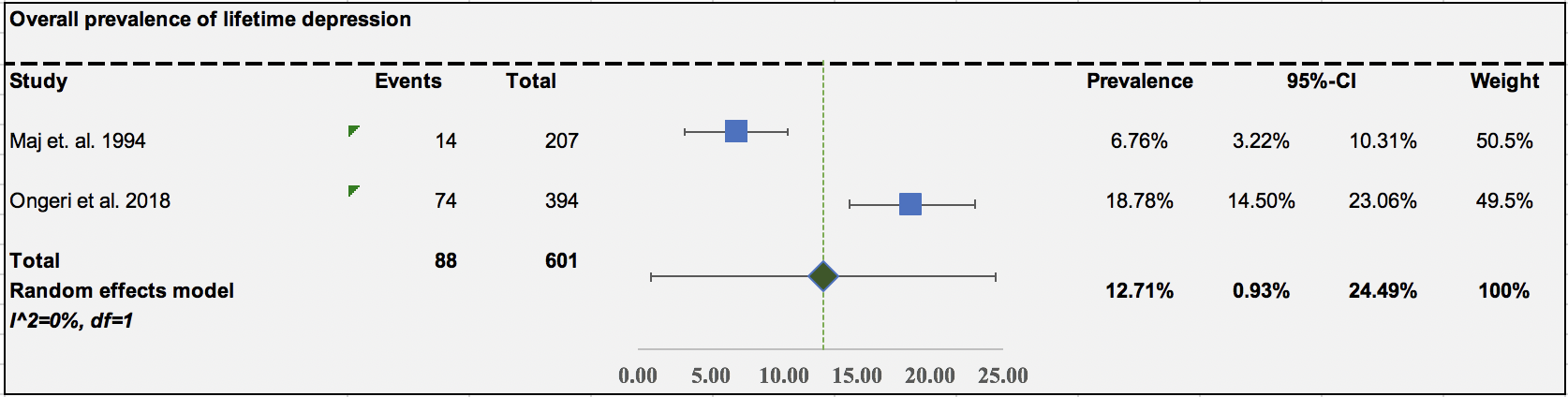
**
3.

**Figure S1.10.**  **Prevalence of depression in Kenya.** A) Current episode of depression and B) Lifetime depression and C) age-specific estimates as reported by Ambugo et al 2014^16^ and on far right the calculated crude (blue) and age-standardized (orange) prevalence (ASP), using standard direct method and WHO standard population.

**Type 2 diabetes**

The systematic review yielded a total of 509 studies, two of which from the grey literature. After removing duplicates, 420 abstracts and 29 full-text studies were assessed for eligibility. Six studies provided data on the overall prevalence of type 2 diabetes^12,25–29^ (Figure S1.3 and Table S1.6). Of note, one of these studies only reported prevalence amongst adults aged 50 and above.^27^ Therefore, their data was not used in the meta-analysis of overall prevalence but only for the corresponding age-specific pooled rates. Five reporting data disaggregated by age (Table S1.6), although one did not provide population numbers by age groups so was excluded from the age-specific meta-analysis.

A crude prevalence of 5.2% (95% CI 3.0 to 7.3) was calculated (Figure S1.11A). For those aged 18-29, 30-39, 40-49, 50-59 and ≥60 prevalence was 1.6% (95% CI 0.0 to 3.3), 2.7% (95% CI 1.8 to 3.5), 4.6% (95% CI 3.0 to 6.2), 5.8% (95% CI 4.8 to 6.8) and 7.6% (95% CI 4.1 to 11.2), respectively (Figure S1.11B ***–*** *blue solid line*). The ASP was calculated as 4.0% (95% CI 2.3 to 5.7).

**Table S1.6. Study details and outcomes available for type 2 diabetes.**

**DM definition in the studies was compared to Kenyan guidelines’ criteria of FPG*≥*7mmol/l, 2h-PG*≥*11.1mmol/l and RPG*≥*11.1mmol/l.*^30^

***No data on the demographic composition of the study population was available to calculate the age-groups.*

*Abbreviations: 2h-PG, 2-hour plasma glucose; Disag., diaggregated; DM, type 2 diabetes mellitus; FPG, fasting plasma glucose; LB, lower bound of 95% confidence interval; UB, upper bound of 95% confidence interval; RCBG, random capillary blood glucose; RPG, random plasma glucose; Rx, on treatment for diabetes.*

| Study | Study period | Sample size | Country | Study Setting | Study design | Disag. by HIV | Disag. by age | Definition* | Crude prevalence (LB-UB) |
| --- | --- | --- | --- | --- | --- | --- | --- | --- | --- |
| Ayah et al. 2013 | June to August 2010 | 2,061 | Kenya | Population-based | Cross-sectional | No | Yes | RCBG + 2h-PG or previous Rx | 3.2%  (2.4-4.0) |
| Christensen et al. 2009 | Not specified | 1,459 | Kenya | Population based | Cross-sectional | No | Yes** | FPG and 2h-PG | 4.0%  (3.0-5.1) |
| Edwards et al. 2015 | January to June 2013 | 2,206 | Kenya | Primary clinic-based | Retrospective analysis of clinical records | Yes | No | FPG | 12.2%  (10.8-13.7) |
| Mathenge et al. 2010 | January 2007 to Dec 2008 | 4,396 | Kenya | Population-based | Cross-sectional | No | Yes | RPG | 6.5%  (5.8-7.3) |
| Oti et al. 2013 | May 2008 to April 2009 | 5,190 | Kenya | Population-based | Cross-sectional | No | Yes | RCBG + 2h-PG or previous Rx | 4.4%  (3.8-4.9) |
| STEPS survey 2015 | 2015 | 4,087 | Kenya | Population-based | Cross-sectional | No | Yes | FPG or previous Rx | 2.4%  (1.9-2.9) |

**A.
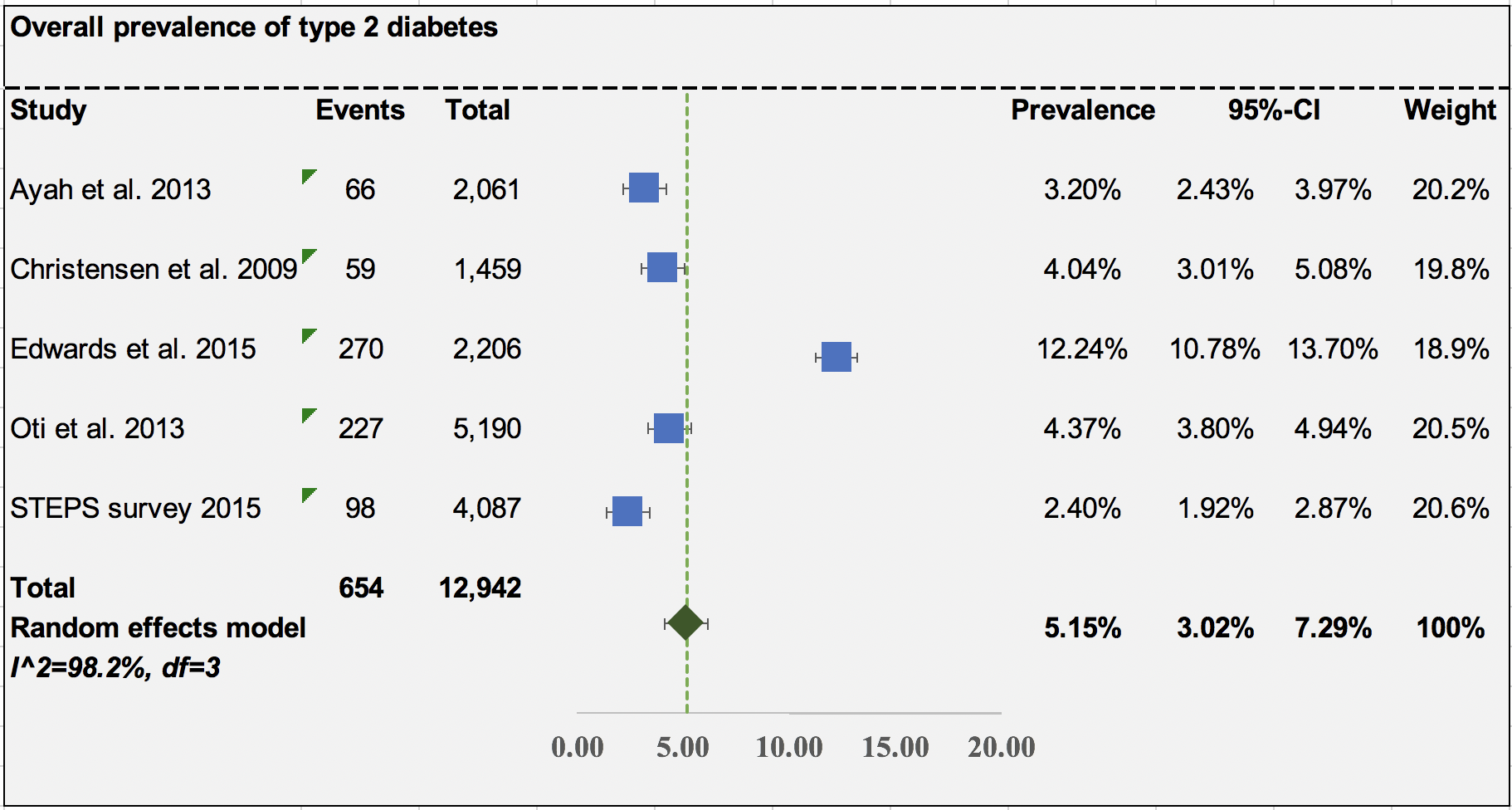
**

**B.**

**Figure S1.11. Prevalence of type 2 diabetes in Kenya.** A) Meta-analysis of the crude prevalence from five studies B) Age-specific prevalence from four studies (dotted lines) and meta-analysis (solid blue line with 95% CIs), and on far right the calculated crude (blue) and age-standardized (orange solid line) prevalence (ASP) from the meta-analysis. ASP used standard direct method and WHO standard population.

**High total cholesterol**

The systematic review yielded a total of 113 studies, of which one was from the grey literature. After removing duplicates, 99 abstracts and nine full-text articles were assessed for eligibility. Three studies provided crude high total cholesterol prevalence estimate (Figure S1.4 and Table S1.7) with two reporting data disaggregated by age.^29,31,32^

Crude prevalence from the three studies was 11.7% (95% CI 11.3 to 12.0) (Figure S1.12A). Prevalence for those aged 18-29, 30-39, 40-49, 50-59 and ≥60 was 8.5% (95% CI 2.3 to 14.7), 11.6% (95% CI 9.7 to 13.4), 10.5% (95% CI 4.0 to 17.1), 14.6% (95% CI 8.9 to 20.4) and 18.1% (95% CI 14.0 to 22.2), respectively. The ASP was calculated as 12.1% (95% CI 7.2 to 17.0), (Figure S1.12B).

**Table S1.7. Study details and outcomes available for high total cholesterol.**

*Abbreviations: Disag., diaggregated; LB, lower bound of 95% confidence interval; UB, upper bound of 95% confidence interval; TC, total cholesterol.*

| Study | Study period | Sample size | Country | Study Setting | Study design | Disag by HIV | Disag by age | Definition | Crude prevalence (LB-UB) |
| --- | --- | --- | --- | --- | --- | --- | --- | --- | --- |
| Chege 2016 | 2006 to 2008 | 1,173 | Kenya | Population-based | Cross-sectional | No | Yes | TC ≥5.2mmol/L | 12.4% (10.3-14.4) |
| Haregu et al. 2016 | May 2008 to April 2009 | 5,190 | Kenya | Population-based | Cross-sectional | No | No | TC ≥5.2mmol/L | 11.8% (10.9-12.8) |
| STEPS survey 2015 | 2015 | 4,169 | Kenya | Population-based | Cross-sectional | No | Yes | TC ≥5.0mmol/L or previously on Rx | 11.3% (10.3-12.3) |

1. **
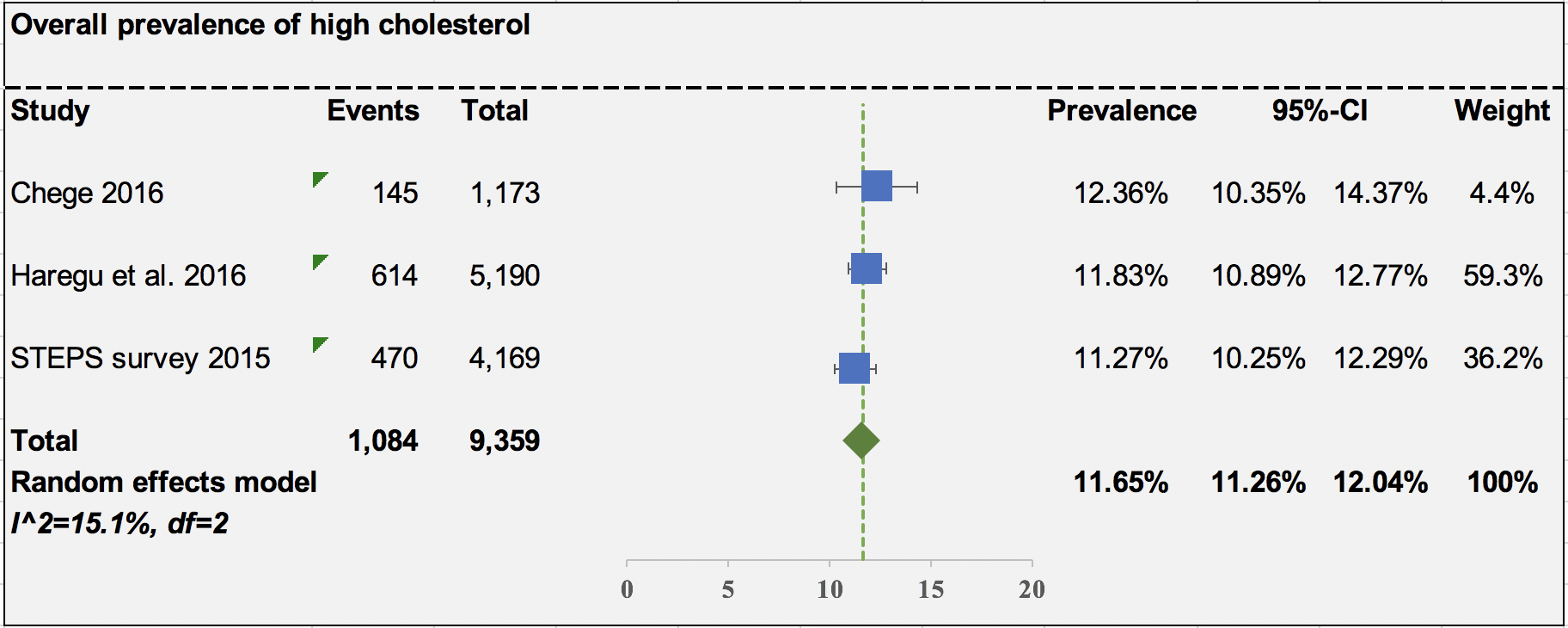
**
2.

**Figure S1.12. Prevalence of high total cholesterol in Kenya.** A) Meta-analysis of the crude prevalence from three studies. B) Age-specific prevalence from two studies (dotted lines) and meta-analysis (solid blue line with 95% CIs) and on far right the calculated crude (blue) and age-standardized (orange) prevalence (ASP) from meta-analysis. ASP used standard direct method and WHO standard population.

**Hypertension**

The search strategy yielded a total of 1,125 individual studies, one of which was from the grey literature. After removing duplicates, 792 abstracts and 64 full-text articles were assessed for eligibility. Twenty-two studies met inclusion criteria and provided point estimates of hypertension prevalence (Figure S1.5 and Table S1.8).^12,27,29,33–51^ Thirteen reported estimates disaggregated by age groups, although two studies did not provide population numbers for age groups and so were excluded from the age-specific meta-analysis (Table S1.8).

Crude prevalence of hypertension from the 22 studies was calculated as 25.6% (95% CI 21.1 to 30.1) (Figure S1.13A). Prevalence for those aged 18-29, 30-39, 40-49, 50-59 and ≥60, was 13.8% (95% CI 9.6 to 18.1), 18.9% (95% CI 13.9 to 24.0), 29.6% (95% CI 23.4 to 35.9), 42.6% (95% CI 36.8 to 48.4) and 52.5% (95% CI 47.6 to 57.4), respectively. The ASP was calculated as 28.7% (23.6 to 33.8) (Figure S1.13B).

**Table S1.8. Study details and outcomes available for hypertension.**

**where measurements are presented as systolic over diastolic blood pressure in mm Hg.*

***researchers reported a risk ratio of the prevalence of hypertension between PLHIV and HIV-negatives, which was found to be non-statistically-significant.*

*Abbreviations: Disag., disaggregated; LB, lower bound of 95% confidence interval; UB, upper bound of 95% confidence interval; TC, total cholesterol; SBP, systolic blood pressure; Rx, on treatment for hypertension*

| Study | Study period | Sample size | Country | Study Setting | Study design | Disag. by HIV | Disag. by age | Definition* | Crude prevalence (LB-UB) |
| --- | --- | --- | --- | --- | --- | --- | --- | --- | --- |
| Carvalho et al. 1989 | Not specified | 176 | Kenya, Brazil and Papua New Guinea | Population-based | Cross-sectional | No | No | Manual ≥140/90 | 5.1% (1.8-8.5%) |
| Chege 2016 | 2006 to 2008 | 1,200 | Kenya | Population-based | Cross-sectional | No | Yes | Manual ≥140/90 | 20.0% (17.5-22.5%) |
| Christensen et al. 2016 | August to November 2005 | 1,139 | Kenya | Population-based | Cross-sectional | No | No | Automated ≥140/90 and/or currently on Rx | 8.5% (6.8-10.2%) |
| Edwards et al. 2015 | January to June 2013 | 1,800 | Kenya | Primary clinic-based | Retrospective analysis of clinical records | Yes | No | Manual ≥140/90 and/or currently on Rx | 11.6% (10.0-13.1%) |
| Etyang et al. 2016 | April 2013 to May 2014 | 986 | Kenya | Population-based | Nested case-control diagnostic accuracy study | No | Yes No data of age-specific N | Manual ≥140/90 | 36.4% (32.6-40.2%) |
| Gomez-Olive et al. 2017 | August 2013 to August 2016 | 2,003 | Kenya, Burkina Faso, Ghana and South Africa | Population-based | Cross-sectional | No | Yes No data on age-specific cases | Manual ≥140/90 and/or currently on Rx | 26.0% (23.8-28.2%) |
| Hendriks et al. 2012 | 2011 (month unspecified) | 2,111 | Kenya, Nigeria, Tanzania and Namibia | Population-based | Cross-sectional | No** | Yes | Automated ≥140/90 and/or currently on Rx | 20.2% (18.3-22.1%) |
| Irazola et al. 2016 | 2012 to 2013 | 239 | Kenya, South Africa, India, Pakistan, Peru, Argentina, Chile and Uruguay | Population-based | Cross-sectional | No | Yes | Automated ≥140/90 and/or currently on Rx | 53.6% (44.3-62.8%) |
| Jenson et al. 2011 | Summer of 2008 | 469 | Kenya | Population-based | Cross-sectional | No | Yes | Manual ≥140/90 | 43.1% (37.1-49.0%) |
| Joshi et al. 2014 | June to August 2010 | 2,045 | Kenya | Population-based | Cross-sectional | No | Yes | Manual ≥140/90 and/or currently on Rx | 12.6% (11.1-14.2%) |
| Kaduka et al. 2012 | August to October 2008 | 539 | Kenya | Population-based | Cross-sectional | No | Yes | Automated ≥130/85 and/or currently on Rx | 64.7% (58.0-71.5%) |
| Mathenge et al. 2010 | January 2007 to December 2008 | 4,396 | Kenya | Population-based | Cross-sectional | No | Yes | Manual ≥140/90 and/or currently on Rx | 50.1% (48.0-52.2%) |
| Olack et al. 2015 | June to August 2013 | 1,528 | Kenya | Population-based | Cross-sectional | No | Yes | Automated ≥140/90 and/or currently on Rx | 27.4% (24.7-30.0%) |
| Onyango et al. 2017 | August to December 2016 | 370 | Kenya | Population-based (within a company in Nairobi) | Cross-sectional | No | Yes | Automated ≥140/90 and/or currently on Rx | 30.0% (24.4-35.6%) |
| Oti et al. 2016 | August 2012 to February 2014 | 4,049 | Kenya | Population-based | Pilot study from a previous model | No | No | Automated ≥140/90 and/or currently on Rx | 24.1% (22.6-25.6%) |
| Pastakia et al. 2013 | March 2010 to November 2011 | 582 | Kenya | Population-based | Cross-sectional | No | No | Screening automated and SBP≥160, followed by confirmation with manual device at a threshold of ≥140/90 | 8.2% (5.9-10.6%) |
| Pastakia et al. 2017 | November 2012 to April 2013 | 879 | Kenya | Population-based | Cross-sectional | No | No | Automated ≥140/90 and/or currently on Rx | 14.2% (11.7-16.7%) |
| Rasmussen et al. 2016 | August to November 2005 | 1,167 | Kenya | Population-based | Cross-sectional | No | No | Automated ≥140/90 | 11.7% (9.8-13.7%) |
| Smith et al. 2017 | 2013 (month unspecified) | 160 | Kenya, India and El Salvador | Primary care clinic-based | Cross-sectional | No | No | Automated ≥140/90 and/or currently on Rx | 36.9% (27.5-46.3%) |
| STEPS survey 2015 | 2015 | 4,493 | Kenya | Population-based | Cross-sectional | No | Yes | Automated ≥140/90 and/or currently on Rx | 26.6% (25.1-28.2%) |
| Van de Vijver et al. 2013 | May 2008 to April 2009 | 5,190 | Kenya | Population-based | Cross-sectional | No | Yes | Automated ≥140/90 and/or currently on Rx | 20.2% (19.0-21.4%) |
| Van de Vivjer et al. 2016 | August 2012 to February 2014 | 2,764 | Kenya | Primary care clinic-based | Intervention study | No | No | Automated ≥140/90 and/or currently on Rx | 23.6% (21.8-25.4%) |

**A.** **
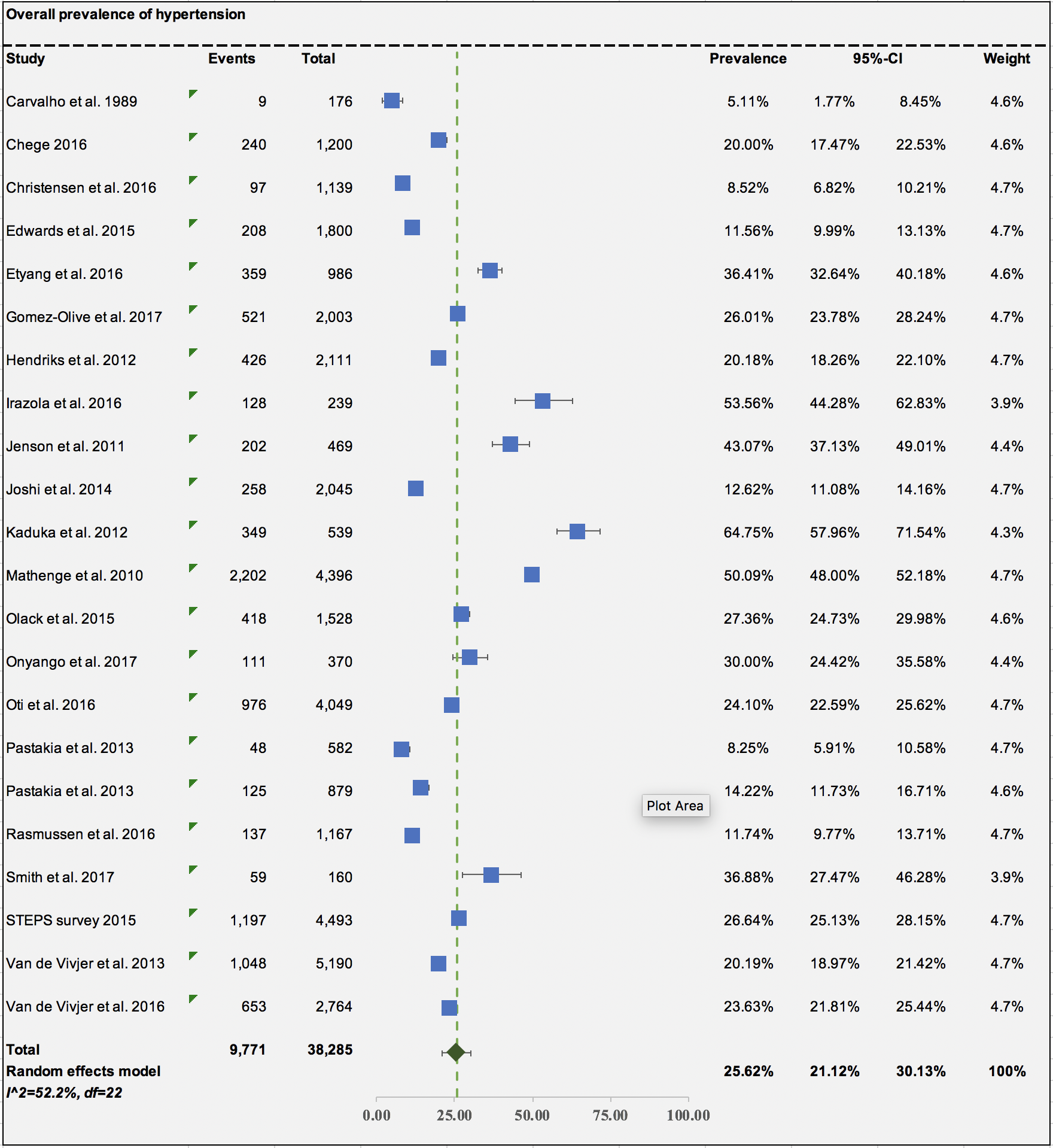
**

**B.**

**Figure S1.13. Prevalence of hypertension in Kenya.** A) Meta-analysis of crude prevalence from 22 studies (N = 53,463). B) Age-specific prevalence from eleven studies (dotted lines) and meta-analysis (solid blue line with 95% CIs) and on far right the calculated crude (blue) and age-standardized (orange solid line) prevalence (ASP) from the meta-analysis. ASP used standard direct method and WHO standard population.

**Human Papillomavirus and related natural history stages**

The systematic review yielded a total of 362 studies from online databases and two from grey literature. After removing duplicates, 286 abstracts and 29 full-text studies were assessed for eligibility, of which eight fulfilled inclusion criteria (Table S1.9).^52–59^ Five studies provided data on the prevalence of HPV infection for the overall population (Figure S1.14A) and six for the population of HIV-positive women (Figure S1.14B). Five studies provided data on the prevalence of CIN 2/3 lesions among the overall population (Figure S1.14C) and seven for the population HIV-positive women (Figure S1.14). Data disaggregated by age in the overall population was available from four of the studies for the case of HPV infection and from two for CIN 2/ lesions (Figure S1.14E and G). On the other hand, further disaggregation of data by age among HIV-positive women was obtained from two studies for HPV infection, but only one for CIN 2/3 lesions (Figure S1.14F and G).

A crude prevalence of HPV infection of 36.5% (95% CI 23.7 to 49.3) was calculated for the overall population (Figure S1.14A) and of 54.7% (95% CI 38.2 to 71.3) for HIV-positive women (Figure S1.14B). For those aged 15-24, 25-29, 30-34, 35-39 and ≥40 prevalence was 31.9% (95% CI 20.8 to 43.0), 32.9% (95% CI 18.7 to 47.2), 29.1% (95% CI 16.8 to 41.5), 33.8% (95% CI 14.0 to 53.5) an1 28.0% (95% CI 14.1 to 41.9) amongst the overall population and of 69.7% (95% CI 42.8 to 96.5), 64.3% (95% CI 44.5 to 84.1), 58.2% (95% CI 28.5 to 87.8), 60.0% (95% CI 39.3 to 80.8) and 56.0% (95% CI 34.8 to 77.3) amongst HIV-positives (Figure S1.14E and F). The ASP was calculated as 30.1% (95% CI 16.4 to 43.8) for the overall population and of 60.6% (95% CI 37.5 to 83.8) for HIV-positives.

For the case of CIN 2/3 lesions, a crude prevalence of 5.7% (95% CI 3.5 to 8.0) was calculated for the overall population (Figure S1.14C) and 13.4% (95% CI 7.3 to 19.5) for HIV-positive women (Figure S1.14D). For those aged 15-24, 25-29, 30-34, 35-39 and ≥40 prevalence was 4.0% (95% CI 0.1 to 7.8), 7.5% (95% CI 4.1 to 10.9), 9.2% (95% CI 5.2 to 13.3), 10.4% (95% CI 5.1 to 15.7) and 5.7% (95% CI 2.6 to 8.8) for the overall population, and 3.3% (95% CI 0.7 to 5.8), 13.3% (95% CI 10.0 to 15.0), 8.4% (95% CI 6.3 to 9.4), 8.6% (95% CI 6.7 to 9.4) and 8.2% (95% CI 3.7 to 10.0) amongst HIV-positives (Figure S1.14G). All included studies ascertained diagnosis of CIN 2/3 lesions through collection of cytological specimens and Papanicolau staining, which in low- and middle-income countries has been shown to have a sensitivity of 0.794 in the overall population^60^ and of 0.718 among HIV-positives.^61^ In order to get the population burden, the number of cases used to calculate crude and age-specific values presented here were therefore inflated by a factor of 1.206 in the overall population and by 1.282 in HIV-positives. The ASP was calculated at 6.3% (95% CI 2.7 to 9.9) for the overall population and at 7.7% (95% CI 4.3 to 9.5) for HIV-positives.

**Table S1.9 Study details and outcomes available for HPV and CIN.**

**where HPV infection was documented by DNA testing and CIN diagnosis by either cytology or histology.*

***prevalence presented refers to HPV for all studies, except for Chung et al. 2019, for which prevalence of CIN 2/3 is presented, given the aim of that particular study*

*Abbreviations: Disag., disaggregated; LB, lower bound of 95% confidence interval; UB, upper bound of 95% confidence interval; Cyt, cytology.*

| Study | Study period | Sample size | Country | Study Setting | Study design | Disag. by HIV | Disag. by age | Definition* | Crude prevalence (LB-UB)** |
| --- | --- | --- | --- | --- | --- | --- | --- | --- | --- |
| Temmerman et al. 1999 | 1994 | 513 | Kenya | Primary clinic-based | Cross-sectional | Yes | Yes | DNA/Cyt | 17.0% (13.4-20.5%) |
| de Vuyst et al. 2003 | 1998 to 2000 | 429 | Kenya | Primary clinic-based | Cross-sectional | Yes | Yes | DNA/Cyt | 44.3% (38.0-50.6%) |
| Yamada et al. 2008 | 2004 to 2005 | 488 | Kenya | Primary clinic-based | Cross-sectional | Yes | Yes | DNA/Cyt | 27.0% (22.4-31.7%) |
| de Vuyst et al. 2010 | 2002 to 2004 | 496 | Kenya | Primary clinic-based | Cross-sectional | No | Yes | DNA/Cyt | 41.9% (36.2-47.6%) |
| Luque et al. 2010 | Not specified | 49 | Kenya, USA | Primary clinic-based | Cross-sectional | No HIV+ only | No | DNA | 22.4% (9.2-35.7%) |
| de Vuyst et al. 2012 | 2009 | 498 | Kenya | Primary clinic-based | Cross-sectional | No HIV+ only | Yes | DNA/Cyt-Hist | 68.7% (61.4-76.0%) |
| Maranga et al. 2013 | 2008 to 2009 | 223 | Kenya | Primary clinic-based | Cross-sectional | Yes | No | DNA/Cyt | 54.3% (44.6-63.9%) |
| Chung et al. 2019 [under review] | 2013 | 6,403 | Kenya | Primary clinic-based | Cross-sectional (baseline data from a randomised controlled trial) | No HIV+ only | Yes | DNA/Cyt | 8.7% (8.0-9.4%) |

**A.**

**B.**

**C.**

**D.**

**E.**

**F.**

**G.**

**Figure S1.14. Prevalence of HPV and CIN 2/3 in Kenya.** A) Meta-analysis of crude prevalence of HPV infection in the overall population from five studies (N = 2,149). B) Meta-analysis of crude prevalence of HPV infection in the HIV-positives population from six studies (N = 913). C) Meta-analysis of crude prevalence of CIN 2/3 lesions in the overall population from five studies (N = 2,150). D) Meta-analysis of crude prevalence of CIN 2/3 lesions in the HIV-positives population from seven studies (N = 797). E) Age-specific prevalence of HPV infection in the overall population from four studies (dotted lines) and meta-analysis (solid blue line with 95% CIs) and on far right the calculated crude (blue) and age-standardized (orange) prevalence (ASP) from the meta-analysis. F) Age-specific prevalence of HPV infection in the HIV-positives population from two studies (dotted lines) and meta-analysis (solid blue line with 95% CIs) and on far right the calculated crude (blue) and ASP (orange) from the meta-analysis. G) Age-specific prevalence of CIN 2/3 lesions in the overall population from two studies (dotted lines), HIV-positives population (solid green line) and meta-analysis (solid blue line with 95% CIs) and on far right the calculated crude (blue) and ASP (orange) from the meta-analysis among the overall population. ASP used standard direct method and WHO standard population.

**References**

1. Stroup, D. F. Meta-analysis of Observational Studies in EpidemiologyA Proposal for Reporting. *JAMA* **283**, 2008 (2000).

2. Moher, D., Liberati, A., Tetzlaff, J. & Altman, D. G. Preferred reporting items for systematic reviews and meta-analyses: the PRISMA statement. *BMJ* **339**, b2535 (2009).

3. Stroup, D. F. *et al.* Meta-analysis of observational studies in epidemiology: a proposal for reporting. Meta-analysis Of Observational Studies in Epidemiology (MOOSE) group. *JAMA* **283**, 2008–2012 (2000).

4. Ministry of Public Health and Sanitation. *Kenya National Strategy for the Prevention and Control of Non-Communicable Diseases 2015-2020*. (2015).

5. Ministry of Health (Kenya). Available at: http://www.health.go.ke/.

6. Kenya National Bureau of Statistics. (2018). Available at: https://www.knbs.or.ke/.

7. National AIDS Control Council (Kenya). Available at: https://nacc.or.ke/.

8. National AIDS & STI Control Programme (Kenya). Available at: https://www.nascop.or.ke/.

9. World Health Organization. World Standard Population. (2001). Available at: http://apps.who.int/healthinfo/statistics/mortality/whodpms/definitions/pop.htm. (Accessed: 1st August 2016)

10. Meta-Analysis Workshops. Common mistakes in Meta-Analysis and How to Avoid Them Fixed-effect vs. Random-effects. (2018). Available at: https://www.meta-analysis-workshops.com/download/common-mistakes2.pdf.

11. Walker, R. *et al.* Stroke incidence in rural and urban Tanzania: A prospective, community-based study. *Lancet Neurol.* **9**, 786–792 (2010).

12. Edwards, J. K. *et al.* HIV with non-communicable diseases in primary care in Kibera, Nairobi, Kenya: characteristics and outcomes 2010-2013. *Trans. R. Soc. Trop. Med. Hyg.* **109**, 440–446 (2015).

13. Stanifer, J. W. *et al.* The epidemiology of chronic kidney disease in Northern Tanzania: a population-based survey. *PloS One* **10**, e0124506 (2015).

14. Peck, R. *et al.* Decreased renal function and associated factors in cities, towns and rural areas of Tanzania: a community-based population survey. *Trop. Med. Int. Health TM IH* **21**, 393–404 (2016).

15. Aillon, J.-L. *et al.* Prevalence, types and comorbidity of mental disorders in a Kenyan primary health centre. *Soc. Psychiatry Psychiatr. Epidemiol.* **49**, 1257–1268 (2014).

16. Ambugo, E. A. Cross-country variation in the sociodemographic factors associated with major depressive episode in Norway, the United Kingdom, Ghana, and Kenya. *Soc. Sci. Med. 1982* **113**, 154–160 (2014).

17. Jenkins, R. *et al.* Prevalence of common mental disorders in a rural district of Kenya, and socio-demographic risk factors. *Int. J. Environ. Res. Public. Health* **9**, 1810–1819 (2012).

18. Jenkins, R. *et al.* Common mental disorder in Nyanza province, Kenya in 2013 and its associated risk factors -an assessment of change since 2004, using a repeat household survey in a demographic surveillance site. *BMC Psychiatry* **15**, 309 (2015).

19. Kwobah, E., Epstein, S., Mwangi, A., Litzelman, D. & Atwoli, L. PREVALENCE of psychiatric morbidity in a community sample in Western Kenya. *BMC Psychiatry* **17**, 30 (2017).

20. Maj, M. *et al.* WHO Neuropsychiatric AIDS study, cross-sectional phase I. Study design and psychiatric findings. *Arch. Gen. Psychiatry* **51**, 39–49 (1994).

21. Monahan, P. O. *et al.* Validity/reliability of PHQ-9 and PHQ-2 depression scales among adults living with HIV/AIDS in western Kenya. *J. Gen. Intern. Med.* **24**, 189–197 (2009).

22. Ndetei, D. M. *et al.* The prevalence of mental disorders in adults in different level general medical facilities in Kenya: a cross-sectional study. *Ann. Gen. Psychiatry* **8**, 1 (2009).

23. Nyongesa, M. K. *et al.* Neurocognitive and mental health outcomes and association with quality of life among adults living with HIV: a cross-sectional focus on a low-literacy population from coastal Kenya. *BMJ Open* **8**, e023914 (2018).

24. Ongeri, L. *et al.* Suicidality and associated risk factors in outpatients attending a general medical facility in rural Kenya. *J. Affect. Disord.* **225**, 413–421 (2018).

25. Ayah, R. *et al.* A population-based survey of prevalence of diabetes and correlates in an urban slum community in Nairobi, Kenya. *BMC Public Health* **13**, 371 (2013).

26. Christensen, D. L. *et al.* Prevalence of glucose intolerance and associated risk factors in rural and urban populations of different ethnic groups in Kenya. *Diabetes Res. Clin. Pract.* **84**, 303–310 (2009).

27. Mathenge, W., Foster, A. & Kuper, H. Urbanization, ethnicity and cardiovascular risk in a population in transition in Nakuru, Kenya: a population-based survey. *BMC Public Health* **10**, 569 (2010).

28. Oti, S. O., van de Vijver, S. J. M., Agyemang, C. & Kyobutungi, C. The magnitude of diabetes and its association with obesity in the slums of Nairobi, Kenya: results from a cross-sectional survey. *Trop. Med. Int. Health TM IH* **18**, 1520–1530 (2013).

29. Ministry of Health. *Kenya STEPwise Survey for Non-Communicable Diseases and Risk Factors 2015 Report*. (2015).

30. Ministry of Public Health and Sanitation. *National Clinical Guidelines for Management of Diabetes Mellitus*. (2010).

31. Chege, P. Multiple cardiovascular disease risk factors in rural Kenya: Evidence from a health and demographic surveillance system using the WHO STEP-wise approach to chronic disease risk factor surveillance. *South Afr. Fam. Pract.* **58**, 54–61 (2016).

32. Haregu, T. N. *et al.* Interlinkage among cardio-metabolic disease markers in an urban poor setting in Nairobi, Kenya. *Glob. Health Action* **9**, 30626 (2016).

33. Chege, P. Multiple cardiovascular disease risk factors in rural Kenya: Evidence from a health and demographic surveillance system using the WHO STEP-wise approach to chronic disease risk factor surveillance. *South Afr. Fam. Pract.* **58**, 54–61 (2016).

34. Carvalho, J. J. M. *et al.* Blood pressure in four remote populations in the INTERSALT Study. *Hypertension* **14**, 238–246 (1989).

35. Christensen, D. L. *et al.* Cardiovascular risk factors in rural Kenyans are associated with differential age gradients, but not modified by sex or ethnicity. *Ann. Hum. Biol.* **43**, 42–49 (2016).

36. Etyang, A. O. *et al.* Clinical and epidemiological implications of 24-hour ambulatory blood pressure monitoring for the diagnosis of hypertension in kenyan adults: A population-based study. *J. Am. Heart Assoc.* **5**, (2016).

37. Gómez-Olivé, F. X. *et al.* Regional and Sex Differences in the Prevalence and Awareness of Hypertension: An H3Africa AWI-Gen Study Across 6 Sites in Sub-Saharan Africa. *Glob. Heart* **12**, 81–90 (2017).

38. Hendriks, M. E. *et al.* Hypertension in sub-Saharan Africa: cross-sectional surveys in four rural and urban communities. *PloS One* **7**, e32638 (2012).

39. Irazola, V. E. *et al.* Hypertension Prevalence, Awareness, Treatment, and Control in Selected LMIC Communities. *Glob. Heart* **11**, 47–59 (2016).

40. Jenson, A., Omar, A. L., Omar, M. A., Rishad, A. S. & Khoshnood, K. Assessment of hypertension control in a district of Mombasa, Kenya. *Glob. Public Health* **6**, 293–306 (2011).

41. Joshi, M. D. *et al.* Prevalence of hypertension and associated cardiovascular risk factors in an urban slum in Nairobi, Kenya: A population-based survey. *BMC Public Health* **14**, 1177 (2014).

42. Kaduka, L. U. *et al.* Prevalence of Metabolic Syndrome among an Urban Population in Kenya. *Diabetes Care* **35**, 887–893 (2012).

43. Olack, B. *et al.* Risk factors of hypertension among adults aged 35–64 years living in an urban slum Nairobi, Kenya. *BMC Public Health* **15**, 1251 (2015).

44. Onyango, M. J., Kombe, I., Nyamongo, D. S. & Mwangi, M. A study to determine the prevalence and factors associated with hypertension among employees working at a call centre Nairobi Kenya. *Pan Afr. Med. J.* **27**, (2017).

45. Oti, S. O. *et al.* Outcomes and costs of implementing a community-based intervention for hypertension in an urban slum in Kenya. *Bull. World Health Organ.* **94**, 501–509 (2016).

46. Pastakia, S. D. *et al.* Screening for diabetes and hypertension in a rural low income setting in western Kenya utilizing home-based and community-based strategies. *Glob. Health* **9**, 21 (2013).

47. Pastakia, S. D. *et al.* Impact of Bridging Income Generation with Group Integrated Care (BIGPIC) on Hypertension and Diabetes in Rural Western Kenya. *J. Gen. Intern. Med.* **32**, 540–548 (2017).

48. Rasmussen, J. B. *et al.* Hemoglobin levels and blood pressure are associated in rural black africans. *Am. J. Hum. Biol. Off. J. Hum. Biol. Counc.* **28**, 145–148 (2016).

49. Smith, M. T. *et al.* Elevated blood pressure in the developing world: a role for clinical pharmacists. *Int. J. Pharm. Pract.* **26**, 334–340 (2018).

50. van de Vijver, S. J. M., Oti, S. O., Agyemang, C., Gomez, G. B. & Kyobutungi, C. Prevalence, awareness, treatment and control of hypertension among slum dwellers in Nairobi, Kenya. *J. Hypertens.* **31**, 1018–1024 (2013).

51. van de Vijver, S. *et al.* Impact evaluation of a community-based intervention for prevention of cardiovascular diseases in the slums of Nairobi: the SCALE-UP study. *Glob. Health Action* **9**, 30922 (2016).

52. Temmerman, M. *et al.* Risk factors for human papillomavirus and cervical precancerous lesions, and the role of concurrent HIV-1 infection. *Int. J. Gynaecol. Obstet. Off. Organ Int. Fed. Gynaecol. Obstet.* **65**, 171–181 (1999).

53. de Vuyst, H. *et al.* Distribution of human papillomavirus in a family planning population in Nairobi , Kenya. *Sex. Transm. Dis.* **30**, 137–142 (2003).

54. Yamada, R. *et al.* Human papillomavirus infection and cervical abnormalities in Nairobi, Kenya, an area with a high prevalence of human immunodeficiency virus infection. *J. Med. Virol.* **80**, 847–855 (2008).

55. De Vuyst, H. *et al.* The prevalence of human papillomavirus infection in Mombasa, Kenya. *Cancer Causes Control CCC* **21**, 2309–2313 (2010).

56. Luque, A. E. *et al.* Prevalence of human papillomavirus genotypes in HIV-1-infected women in Seattle, USA and Nairobi, Kenya: Results from the Women’s HIV Interdisciplinary Network (WHIN). *Int. J. Infect. Dis.* **14**, e810–e814 (2010).

57. De Vuyst, H. *et al.* Prevalence and determinants of human papillomavirus infection and cervical lesions in HIV-positive women in Kenya. *Br. J. Cancer* **107**, 1624–1630 (2012).

58. Maranga, I. O. HIV Infection Alters the Spectrum of HPV Subtypes Found in Cervical Smears and Carcinomas from Kenyan Women. *Open Virol. J.* **7**, 19–27 (2013).

59. Greene, S. A. *et al.* Effect of cryotherapy vs loop electrosurgical excision procedure (LEEP) on cervical disease recurrence among women with HIV and high-grade cervical lesions in Kenya: a randomized clinical trial. *JAMA* (2019).

60. Castanon, A. *et al.* Systematic Review and Meta-Analysis of Individual Patient Data to Assess the Sensitivity of Cervical Cytology for Diagnosis of Cervical Cancer in Low- and Middle-Income Countries. *J. Glob. Oncol.* **3**, JGO.2016.008011 (2017).

61. Chung, M. H. *et al.* Comparing Papanicolau smear, visual inspection with acetic acid and human papillomavirus cervical cancer screening methods among HIV-positive women by immune status and antiretroviral therapy. *Aids* **27**, 2909–2919 (2013).
