# Supplementary material for: Mapping the Current and Future Noncommunicable Disease Burden in Kenya by Human Immunodeficiency Virus Status: A Modeling Study
Source: Clin Infect Dis. 2019 Nov 17;71(8):1864–73. doi: 10.1093/cid/ciz1103 (PMC8240998; doi:10.1093/cid/ciz1103)
Supplement: ciz1103_suppl_Supplement_2 [file ciz1103_suppl_supplement_2.docx]

**Supplement 2. Model Technical details, model checks and additional results**

**A. Model Overview**

This supplementary material provides technical details of the model design, parameter values, model assumptions, and results of model checks. The model of non-communicable diseases (NCDs) in Kenya is an adaptation of a previous model,^1^ with this supplement outlining the key modifications to the model structure. Additional details on the basic model structure can be found in the supplement by Smit and colleagues.^1^ Figure S2.1 shows the basic model design.

**Figure S2.1. Schematic of the multi-disease model for Kenya.** The model simulates demography (blue), the HIV epidemic (orange), and non-communicable diseases (green), and accounts for key interactions between demographic and disease-specific factors (red arrows to individual conditions and group of conditions).

**Cervical cancer is higher in HIV-positive women, driven by the higher risk for HPV infection in PLHIV.*

*Abbreviations: Human Immunodeficiency Virus (HIV); antiretroviral therapy (ART); cardiovascular disease (CVD); human papillomavirus (HPV); cervical intraepithelial neoplasia (CIN); carcinoma in situ (CIS).*

The model is an individual-based model of the entire Kenyan population, simulating births, death, HIV infection, disease progression and treatment and development of NCDs. These include cardiovascular disease (CVD – encompassing ischemic heart disease (IHD) and stroke), chronic kidney disease (CKD), depression, type II diabetes, high cholesterol, hypertension several key cancers (including breast, cervical, colorectal, leukaemia, liver, oesophageal, prostate, stomach and ‘other’ cancer, where ‘other’ refers to all cancers except the aforementioned). Cervical cancer is simulated by including a natural history model of human papillomavirus (HPV) infection and progression through pre-cancerous lesions (Figure S2.1 – light green). The model uses available data for the period 1950 to 2015 to inform trends, fits to NCD data collated by original systematic reviews and meta-analyses, with specific fitting procedures described below. The model is used to quantify current burdens of NCD by HIV status in 2018 at the national level and used to forecast future trends in the period 2018 and 2035.

**B. Technical details**

The mechanisms of the model reply on ‘scheduling’ events probabilistically at the start of a person’s life (e.g. their death) so that trends across the whole population recreate observed demographic, epidemiological and clinical trends (e.g. age- and-sex-specific mortality by calendar time). Events are determined probabilistically while accounting for a person’s characteristics (age, sex, pre-existing condition) and calendar time. For example, an older individual or a person with pre-existing hypertension is more likely to have a CVD event compared to a younger individual without hypertension. As the model runs forward in time and new events occur, these can influence future events or states. For example, a patient starting antiretroviral therapy (ART) would have a reduced risk of a premature death, thus, their date of death may be re- scheduling for later in life or a patient who just developed hypertension may develop CKD later in life.

*Demographic Factors*

Demographic factors, specifically age composition at the start of the model, and age-specific fertility rates, and age-and-sex-specific mortality rates between 1950 and 2035 were assigned probabilistically based on data from the United Nations World Population Prospect (UNWPP), accounting for changes over time,^2^ and projections beyond 2018 assuming a medium variance in fertility and mortality rates. Rates are presented by 5-year time periods, with the model assuming that rates within these periods are constant. Cause-specific mortality was parameterised by simultaneously fitting to overall mortality estimates provided by UNWPP between 1950 and 2018 and to cause-specific estimates from the Institute for Health Metrics and Evaluation’s 2016 Global Burden of Disease estimates for Kenya.^3^

*HIV epidemic*

Age-and-sex-specific HIV incidence rates (including paediatric infection) and ART initiation rates by CD4 count were taken from the official UNAIDS (The Joint United Nations Programme on HIV and AIDS) estimates for Kenya, accounting for historic changes in ART eligibility criteria.^4^ UNAIDS estimates are derived through fitting a model to available data, largely prevalence estimates in Demographic and Health Surveys from Kenya. Parameters for CD4 count at seroconversion, CD4 count progression rates and mortality by CD4 count have been described previously^1^ and are based on estimates for Sub-Saharan Africa as produced by Mangal et al.^5^ Projections of the number of new HIV-infections and people starting ART assume that HIV incidence to remains stable at 2017 levels and that ART coverage increases steadily to reach a level of coverage consistent with 90:90:90 targets by 2020.

*NCDs*

The model simulates a number of NCDs, including cardiovascular disease (CVD), chronic kidney disease (CKD), depression, diabetes, high cholesterol, hypertension several key cancers (including breast, cervical, colorectal, leukaemia, liver, oesophageal, prostate, stomach and ‘other’ cancer, where ‘other’ refers to all cancers except the aforementioned). The risk of developing these is assigned probabilistically while accounting for varying NCD risk by age, for the propensity for a pre-existing condition to be associated with an increased risk to develop another (e.g. for hypertension to increase the risk of having a stroke and HIV to increase the risk of developing hypertension and CKD). These links are illustrated by the red arrow in Figure S2.1, with parameters defining these described in Table S2.1.

To parameterise NCDs for Kenya, original systematic reviews and meta-analyses were carried out, summarising in-country evidence of prevalence or incidence of all NCDs except cancers. For cancers, in-country estimates were obtained from Cancer Incidence in 5 Continents (version XI - IARC) for Kenya were collated.^6^ Full details of the systematic reviews and meta-analyses are available in Supplement 1.

Briefly, Medline and Embase were searched from inception to May 2018 for population-based or primary care-based studies reporting on either prevalence or incidence of the NCDs of interest. Where evidence was not available for Kenya, the systematic review was extended to Tanzania, assuming this neighbouring country to have comparable demography, burden of disease and healthcare profile. Were more than one study was found to report estimates of NCDs, meta-analysis was used to pool the results. A summary of the NCD data collated in this way is presented in Table S2.2.

Parameters defining NCDs in the model were obtained by fitting to age-specific NCD prevalence or incidence (as appropriate) estimates collated by the systematic review and meta-analysis or IARC data in the case of cancer. This was done by varying age-specific NCD incidence parameters, cause-specific mortality risk and CVD case-fatality risk, while keeping parameters for NCD risk by pre-existing condition (Table S2.1. and Figure S2.1 – red arrows) fixed. Of note, and as mentioned in the main manuscript and Supplement 1, no data was found on IHD in Kenya. Instead, the model assumed a factor four increased incidence, compared to stroke data from Tanzania^7^, based on a large European study of CVD incidence and the relative risk of stroke versus IHD reported there.^8^

**Table S2.1. Model parameters defining relative risk of developing individual NCDs given pre-existing NCD or HIV infection.**

*Abbreviations: human immunodeficiency virus (HIV); human papillomavirus (HPV); antiretroviral therapy (ART); cervical intraepithelial neoplasia (CIN); carcinoma in situ (CIS).*

**referring to all non-AIDS defining cancers other than the aforementioned and cervical cancer.*

| Association | Hazard ration (95% CI) | Reference: setting |
| --- | --- | --- |
| Non-HIV-related | | |
| Incidence of stroke given pre-existing diabetes vs stroke with no pre-existing diabetes | 2.31 (1·83–2·92) | Worm et al.: Europe, Argentina, Australia, USA.^9^ |
| Incidence of stroke given pre-existing hypertension vs stroke with no pre-existing hypertension | 1·26 (0·88–1·62) | Worm et al.: Europe, Argentina, Australia, USA.^9^ |
| Onset of hypertension given pre-existing diabetes vs hypertension with no pre-existing diabetes | 1·40 (1·19–1·64) | Smit et al: The Netherlands.^10^ |
| Chronic kidney disease given pre-existing diabetes vs chronic kidney disease with no pre-existing diabetes | 1·50 (1·05–2·15) | Mocroft et al: Europe, Argentina and Israel. ^11^ |
| Chronic kidney disease given pre-existing hypertension vs chronic kidney disease with no pre-existing hypertension | 1·69 (1·26–2·27) | Mocroft et al: Europe, Argentina and Israel.^11^ |
| HIV-related | | |
| Hypertension given HIV infection vs hypertension without HIV infection | 1·49 | Schouten et al.: The Netherlands.^12^ |
| Chronic kidney disease given HIV infection vs chronic kidney disease without HIV infection | 2·04 | Schouten et al.: The Netherlands.^12^ |
| Depression given HIV infection vs depression without HIV infection | 3·1 | Do et al. : USA.^13^ |
| HPV infection given HIV infection and being ART-naïve or on ART for <2 years vs HPV infection without HIV infection or with HIV infection and on ART for ≥2 years | 1·63 (1·26-2·11) | Looker et al. ^14^: global systematic review and meta-analysis |
| Clearance of HPV infection given HIV infection and being ART-naïve or on ART <2 years vs clearance of HPV infection without HIV or with HIV infection and on ART for ≥2 years | 0·52 (0·62-0·84) | Looker et al. ^14^: global systematic review and meta-analysis |
| Risk of transitioning from HPV to CIN2/3 with HIV infection and being ART-naïve or on ART for <2 years vs transition from HPV to CIN2/3 without HIV or with HIV infection and on ART for ≥2 years | 1·32 (1·10-1·58) | Liu et al. ^15^: global systematic review and meta-analysis |
| Cancer (type-specific) given HIV infection vs. cancer without HIV infection:   - Breast - Cervical - Colorectal - Leukaemia - Liver - Oesophageal - Prostate - Stomach - Other cancers* | 0·7  3·2  0·6  1·2  3·2  1·2  0·5  0·7  1·2 | Hernández-Ramírez et al.: registry-linkage study from USA cohorts of PLHIV, compared to the general population, from 1996 to 2012.^16^ |

**Table S2.2. Summary of NCD prevalence and incidence data for Kenya from in-depth literature review.** Study period year is based on year of the relevant study, where more than one study was combined in a meta-analysis mean calendar year was calculated from included studies.

*Abbreviations: human immunodeficiency virus (HIV); human papillomavirus (HPV); cervical intraepithelial neoplasia (CIN); carcinoma in situ (CIS).*

*Collated through an original systematic review and meta-analysis (were more than on study found)

**Obtained from IARC^6^

† defined as having had an episode in the past 12months.

‡ sum of incidence of all non-AIDS defining cancers minus incidence of aforementioned cancers.

| Disease | Age-specific prevalence (95% CI) | Crude prevalence (95% CI) | Age-standardised prevalence (95% CI) | Country/Source | Study Period | Reference |
| --- | --- | --- | --- | --- | --- | --- |
| Chronic kidney disease*   - 18 to 29 - 30 to 39 - 40 to 49 - 50 to 59   ≥60 | 5·9% (0·7 to 11·2)  6·2% (3·5 to 8·8)  10·4% (6·5 to 14·3)  12·4% (8·1 to 16·6)  14·4% (7·6 to 21·3) | 10·1% (6·2 to 14·0) | 9·2% (4·6 to 13·8) | Kenya and Tanzania, pooled using meta-analysis | 2014 | ^17–19^ |
| Depression*†   - 18 to 29 - 30 to 44 - 45 to 59 - 60 to 69   70-79  ≥80 | 3·2% (2·5 to 3·9)  9·0% (7·3 to 10·7)  4·9% (3·1 to 6·7)  7·4% (3·3 to 11·5)  12·8% (5·4 to 20·2)  4·4 (0 to 12·8) | 8·5% (5·1 to 11·8) | 6·3% (4·3 to 8·5) | Kenya | 2003 | ^20–28^ |
| Type 2 diabetes*   - 18 to 29 - 30 to 39 - 40 to 49 - 50 to 59 - ≥60 | 1·6% (0·0 to 3·3)  2·7% (1·8 to 3·5)  4·6% (3·0 to 6·2)  5·8% (4·8 to 6·8)  7·6% (4·1 to 11·2) | 5·2% (3·0 to 7·3) | 4·0% (2·3 to 5·7) | Kenya, pooled using meta-analysis | 2015 | ^17,29–33^ |
| High total cholesterol*   - 18 to 29 - 30 to 39 - 40 to 49 - 50 to 59   ≥60 | 8·5% (2·3 to 14·7)  11·6% (9·7 to 13·4)  10·5% (4·0 to 17·1)  14·6% (8·9 to 20·4)  18·1% (14·0 to 22·2) | 11·7% (11·3 to 12·0) | 12·1% (7·2 to 17·0) | Kenya, pooled using meta-analysis | 2015 | ^33–35^ |
| Hypertension*   - 18 to 29 - 30 to 39 - 40 to 49 - 50 to 59 - ≥60 | 13·8% (9·6 to 18·1)  18·9% (13·9 to 24·0)  29·6% (23·4 to 35·9)  42·6% (36·8 to 48·4)  52·5% (47·6 to 57·4) | 25·6% (21·1 to 30·1) | 28·7% (23·6 to 33·8) | Kenya, pooled using meta-analysis | 2015 | ^17,31,33,34,36–53^ |
| Cervical HPV infection in the overall population*  15-24  25-29  30-34  35-39  ≥40 | 31·9% (20·8 to 43·0)  32·9% (18·7 to 47·2)  29·1% (16·8 to 41·5)  33·8% (14·0 to 53·5)  28·0% (14·1 to 41·9) | 36·5% (23·7 to 49·3) | 30·1% (16·4 to 43·8) | Kenya | 2001 | ^54–58^ |
| Cervical HPV infection in the HIV+ population*  15-24  25-29  30-34  35-39  ≥40 | 69·6% (42·8 to 96·5)  64·3% (44·5 to 84·1)  58·2% (28·5 to 87·8)  60·0% (39·3 to 80·8)  56·0% (34·8 to 77·3) | 54·7% (38·2 to 71·3) | 60·6% (37·5 to 83·8) | Kenya | 2006 | ^56,58–62^ |
| CIN 2/3 lesions in the overall population*  15-24  25-29  30-34  35-39  ≥40 | 4·0% (0·1 to 7·8)  7·5% (4·1 to 10·8)  9·2% (5·2 to 13·3)  10·4% (5·1 to 15·7)  5·7% (2·6 to 8·8) | 5·7% (3·5 to 8·0) | 6·3% (2·7 to 9·8) | Kenya | 1997 | ^54–58^ |
| CIN 2/3 lesions in the HIV+ population*  15-24  25-29  30-34  35-39  ≥40 | 3·3% (0·7 to 5·8)  13·3% (10·0 to 15·0)  8·4% (6·3 to 9·4)  8·6% (6·7 to 9·4)  8·2% (3·7 to 10·0) | 13·4% (7·3 to 19·5) | 7·7% (4·3 to 9·5) | Kenya | 2013 | ^56,58–63^ |
|  | ***Age-specific incidence (95% CI)*** | ***Crude incidence (95% CI)*** | ***Age-standardised incidence (95% CI)*** |  |  |  |
| Stroke  18 to 44  45 to 54  55 to 64  65 to 74  75 to 84  ≥85 | 9·3 (4·7 to 16·6)  91·1 (74·4 to 109·7)  220·5 (193·8 to 249·3)  629·1 (584·0 to 677·5)  1,432·6 (1,361·7 to 1,506·1)  1,933·7 (1,850·2 to 2,019·9) | 83·9 (67·7 to 101·9) | 114·8 (102·7 to 129·4) | Tanzania | 2003-2006 | ^7^ |
| Breast cancer**  0 to 4  5 to 9  10 to 14  15 to 19  20 to 24  25 to 29  30 to 34  35 to 39  40 to 44  45 to 49  50 to 54  55 to 59  60 to 64  65 to 69  70+ | 0·0  0·0  0·0  0·5  0·1  5·7  16·6  33·7  55·7  83·2  111·3  139·8  161·6  163·5  145·8 | 23·4 | 40·3 | Kenya | 2018 | ^64^ |
| Cervical cancer**  0 to 4  5 to 9  10 to 14  15 to 19  20 to 24  25 to 29  30 to 34  35 to 39  40 to 44  45 to 49  50 to 54  55 to 59  60 to 64  65 to 69  70+ | 0·0  0·0  0·0  0·1  0·6  2·8  11·4  25·0  62·6  77·4  124·5  148·2  150·4  190·7  150·8 | 20·5 | 33·8 | Kenya | 2018 | ^64^ |
| Colorectal cancer**  0 to 4  5 to 9  10 to 14  15 to 19  20 to 24  25 to 29  30 to 34  35 to 39  40 to 44  45 to 49  50 to 54  55 to 59  60 to 64  65 to 69  70+ | 0·0  0·0  0·1  0·5  0·5  0·8  1·8  2·2  6·3  11·5  20·3  30·5  44·6  52·2  55·3 | 4·5 | 9·3 | Kenya | 2018 | ^64^ |
| Leukaemia**  0 to 4  5 to 9  10 to 14  15 to 19  20 to 24  25 to 29  30 to 34  35 to 39  40 to 44  45 to 49  50 to 54  55 to 59  60 to 64  65 to 69  70+ | 1·8  2·3  2·1  2·0  2·1  2·2  2·4  3·1  4·2  5·3  7·0  9·6  23·0  15·6  22·8 | 3·3 | 4·7 | Kenya | 2018 | ^64^ |
| Liver cancer**  0 to 4  5 to 9  10 to 14  15 to 19  20 to 24  25 to 29  30 to 34  35 to 39  40 to 44  45 to 49  50 to 54  55 to 59  60 to 64  65 to 69  70+ | 0·0  0·0  0·1  0·3  0·6  0·7  0·8  2·2  3·8  6·5  10·1  15·8  12·3  30·8  34·3 | 2·6 | 5·3 | Kenya | 2018 | ^64^ |
| Oesophageal cancer**  0 to 4  5 to 9  10 to 14  15 to 19  20 to 24  25 to 29  30 to 34  35 to 39  40 to 44  45 to 49  50 to 54  55 to 59  60 to 64  65 to 69  70+ | 0·0  0·0  0·0  0·5  0·3  0·8  0·4  4·0  10·1  19·8  34·0  60·6  88·8  108·0  133·4 | 8·6 | 18·4 | Kenya | 2018 | ^64^ |
| Prostate cancer**  0 to 4  5 to 9  10 to 14  15 to 19  20 to 24  25 to 29  30 to 34  35 to 39  40 to 44  45 to 49  50 to 54  55 to 59  60 to 64  65 to 69  70+ | 0·0  0·0  0·0  0·3  0·3  0·0  0·6  0·1  0·2  2·4  8·0  38·0  98·2  242·7  440·6 | 11·3 | 30·8 | Kenya | 2018 | ^64^ |
| Stomach cancer**  0 to 4  5 to 9  10 to 14  15 to 19  20 to 24  25 to 29  30 to 34  35 to 39  40 to 44  45 to 49  50 to 54  55 to 59  60 to 64  65 to 69  70+ | 0·1  0·0  0·1  0·3  0·5  0·2  0·5  0·7  2·7  7·0  13·1  25·0  45·8  63·8  79·7 | 4·2 | 9·3 | Kenya | 2018 | ^64^ |
| Other cancers**‡  0 to 4  5 to 9  10 to 14  15 to 19  20 to 24  25 to 29  30 to 34  35 to 39  40 to 44  45 to 49  50 to 54  55 to 59  60 to 64  65 to 69  70+ | 9·8  9·3  9·1  11·1  13.0  14·2  12·5  8·9  7·9  12·3  35  66·4  102  108·8  81·8 | 15·6 | 23·5 | Kenya | 2018 | ^64^ |

*Natural history model of HPV to cervical cancer*

To simulate the incidence of invasive cervical cancer (ICC), a compartmental model of HPV infection and progression through mutually exclusive stages of cervical disease namely, HPV infection, cervical intraepithelial neoplasia (CIN) grade 1, CIN grade 2/3, carcinoma in situ (CIS) and invasive cervical cancer (ICC) was developed and incorporated into the main model (Figure S2.2). The model simulates new events of HPV infection amongst women 15 to 65 years old and transition to cervical disease stages probabilistically, while explicitly accounting for differences by HIV status and age.


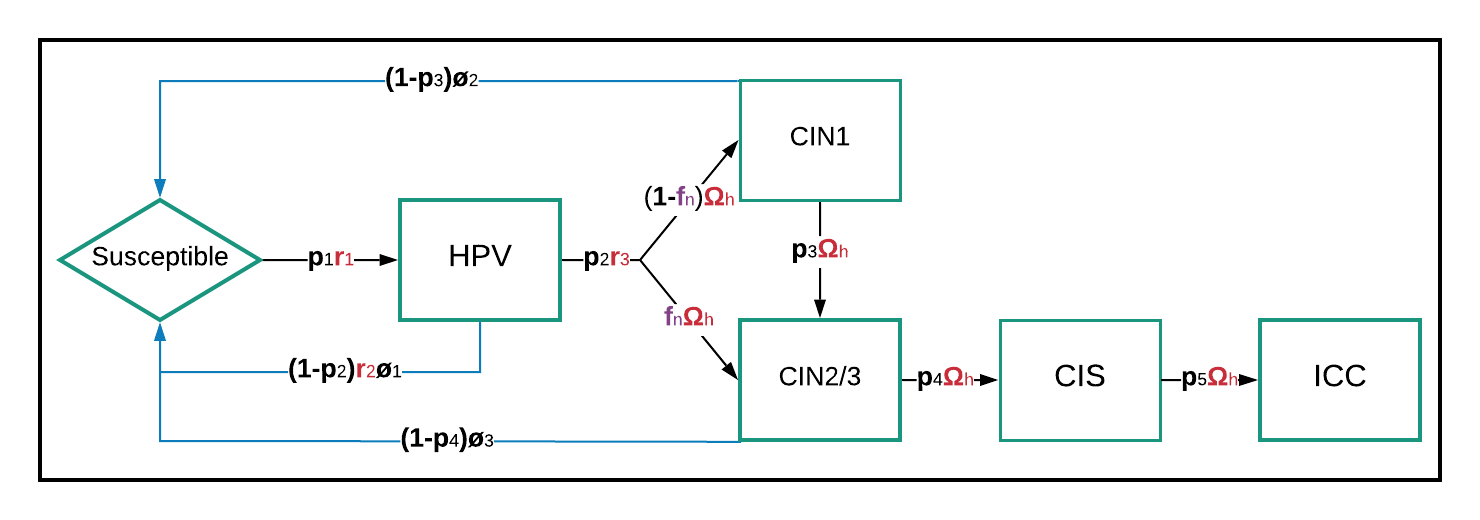


**Figure S2.2. Natural history model of HPV disease and progression to cervical cancer.** Transition between the mutually exclusive stages of related cervical disease are shown with the black arrows and clearance with the blue arrows, with key differences by HIV status shown in red and by age shown in purple.

*Abbreviations: Human Immunodeficiency Virus (HIV); antiretroviral therapy (ART); human papillomavirus (HPV); cervical intraepithelial neoplasia (CIN); carcinoma in situ (CIS); invasive cervical cancer (ICC).*

In the model, individuals can recover from either HPV infection, CIN 1 or CIN 2/3 (Figure S2.2, blue arrows). Upon recovery, individuals are reassessed for further reinfections probabilistically, accounting for risk by age and HIV status. The model assumes no-gained immunity amongst recovered individuals. That is, the model does not account for distinctions of infection by different HPV genotypes and no coverage of previous vaccination against HPV was assumed. Finally, the model assumes that women before the age of 26 had a risk of ‘instantaneous’ transition from HPV infection to CIN 2/3, based on evidence from cohort data.^65^

Four evidence-based assumptions of the difference in the natural history of HPV between HIV-positive and HIV-negative women were made, based on data from several studies ^14,15,66^ (Table S2.3);

- HIV-positive women had a higher risk of HPV infection,
- HIV-positive women had a lower probability of HPV infection recovery (clearance),
- HIV-positive women had a higher risk of progression from CIN 1 to CIN 2/3 lesions
- Women on ART for 2 years or more were assumed to have the same natural history than HIV-negative women.

Model parameters including transition probabilities and rates of transition were established by fitting

simultaneously to available age- and HIV-specific prevalence of data of HPV (any genotype), related cervical disease (Table S2.1), mortality as per GBD estimates, and the standardized incidence rate amongst HIV-positive women compared to HIV-negative women from two American studies and one South African study.^16,67,68^ Parameter values are listed in Table S2.3.

**Table S2.3.**  **Model parameters of the natural history of HPV-related cervical disease by HIV status in Kenya.**

*† Where intervals of values is specified, parameters were drawn uniformly.*

**Cumulative (lifetime) probability of HPV infection among HIV-negative women*

***HIV-related risk is compared in HIV-positive women who are ART-naïve or on ART for <2 years versus HIV-negative women or HIV-positive women who are on ART for* ≥*2 years*

| Parameter | Description | Parameter values^†^ | Setting [reference] |
| --- | --- | --- | --- |
| p | Overall probability *p* of progressing between two stages *j* of the natural history of disease:   1. Susceptible to infected* 2. HPV to CIN (any grade) 3. CIN 1 to CIN 2/3 4. CIN 2/3 to CIS 5. CIS to ICC | 0·66  0·3  0·5  0·75  1·0 | [^69^]  Fitted  [^70^]  [^70^]  Assumed |
| r | Risk ratio *r* of transitioning between two stages given higher HIV-related risk**:   1. Susceptible to HPV 2. HPV to susceptible 3. HPV to CIN (any grade) | 1·47  0·5  1·3 | [^14^]  [^14^]  [^15^] |
| f_n_ | Probability *f* of transition straight from HPV to CIN 2/3. This varies by HIV status and age, *n*:   1. Among HIV-negatives <26 years old 2. Among HIV-positives <26 years old 3. Amongst women ≥26 years old | 0·076  0·15  0 | [^65^]  [^65^]  [^65^] |
| Ø | Annual rate of clearance Ø, uniformly drawn from parameter range, for specific stages:   1. HPV to susceptible 2. CIN 1 to susceptible 3. CIN 2/3 to susceptible | 0·2 to 2  0·83 to 1·5  0·67 to 2·11 | Fitted  [^71^]  [^71^] |
| Ω_h_ | Annual rate of progression Ω, uniformly drawn from parameter range, for HIV status *h.*  Regardless of HIV status:   1. HPV infection to CIN (any grade, any HIV status)   Amongst HIV-negative or HIV-positive on ART for ≥2 years:   1. CIN1 to CIN 2/3 2. CIN 2/3 to CIS 3. CIS to ICC   Amongst HIV-positive ART-naive or on ART for <2 years:   1. CIN 1 to CIN 2/3 2. CIN 2/3 to CIS 3. CIS to ICC | 1 to 5  0·1 to 0·33  0·0416 to 0·33  0·0125 to 0·33  0·2 to 0·5  0·1 to 365  0·02 to 365 | [^71,72^]  [^65,71,72^]  Fitted  Fitted  [^65,71,72^]  Fitted  Fitted |

**C. Model checks**

A number of model checks were carried out to ensure that the model was able to reconstruct demographic and epidemiological trends robustly. This was done by comparing model output to available data. The results are presented in Figure S2.3 to Figure S2.8. They show that the model performed robustly when comparing model output against historic data and short-term, and long-term projections by the UNWPP.

**A. B.**

**C. D.**

**Figure S2.3. Comparison of demographic outputs from the model to United Nations World Prospect (UNWPP) data for Kenya and to data from the Kenya National Bureau of Statistics (KNBS).** **A.** Total annual population from 1950 to 2015 compared to UNWPP. **B.** Population numbers in 2009 compared to KNBS. **C.** Population by sex and age groups in 2015 compared to UNWPP, with the extreme of horizontal bars illustrating differences between model projections and the data. **D.** Population by age and Sex in 2035 compared to UNWPP.

Source: UNWPP^2^ and KNBS.^73^

**A. B.**

**C.**

**Figure S2.4. Comparison of HIV related outcomes as generated by the model to UNAIDS data for Kenya. A.** Annual number of new HIV infections. **B.** Annual number of total HIV-positive people. **C.** Number starting ART by CD4 count per year.

Source: UNAIDS.^4^

**Figure S2.5. Comparison of the proportion of total death by cause as generated by the model compared to Institute for Health Metrics and Evaluation’s 2016 Global Burden of Disease estimates for Kenya. ‘**Other causes’ refers to any cause other than those explicitly mentioned in the figure and include infectious disease and injury related mortality. Source: IHME.^3^

**A. B.**

**C. D.**

**E. F.**

**G.**

**Figure S2.6. Comparison of age-specific prevalence or incidence of NCDs as generated by the model compared to data collated by the systematic review and meta-analysis. A.** Chronic kidney disease in 2014; **B.** Depression (defined as having depression in the past 12 months) in 2003; **C.** Type II Diabetes in 2015**; D.** High total cholesterol in 2015; **E.** Hypertension in 2015, **F.** Ischemic heart disease between 2003 and 2006, **G.** Ischemic stroke between 2003 and 2006. Calendar year for comparison was based on calendar time of the relevant study; where more than one study was combined in a meta-analysis, mean calendar year was calculated from included studies. Error bars represent the 95% confidence intervals.

**Figure S2.7. Comparison of age standardized prevalence of NCDs as generated by the model compared to the data collated by the systematic review and meta-analysis.** Error bars represent the 95% confidence intervals.

**A. B.**

**C. D.**

**E.**

**Figure S2.8. Comparison of HPV and related pre-cancerous stage epidemiology by HIV status as generated by the model compared to data collated for Kenya. A.** HPV prevalence in 2001 general population; **B.** HPV prevalence in HIV-positive women in 2006 ; **C.** CIN2/3 lesions prevalence in general population in 1997 ; **D.** CIN2/3 lesions prevalence in HIV-positive women in 2013; **E.** To the left, ICC incidence in the overall population and in HIV-positive women from 2008 to 2012, and on the far right, standardised incidence rate (mean and 95% CIs) of ICC between HIV-positive and negative women, compared to American multi-cohort data.^67,74^ Calendar year for comparison was based on calendar time of the relevant study; where more than one study was combined in a meta-analysis, mean calendar year was calculated from included studies. Error bars represent the 95% confidence intervals.

**A.****B.**

**C.****D.**

**E.****F.**

**G.****H.**

**I.**

**Figure S2.9. Comparison of age-specific incidence of cancers as generated by the model compared to IARC data. A.** Breast cancer; **B.** Cervical cancer; **C.** Colorectal cancer; **D.** Leukaemia; **E.** Liver cancer; **F.** Oesophageal cancer; **G.** Prostate cancer; **H.** Stomach cancer; **I.** Other cancers. IARC collated cancer incidence data in the overall population of Kenya from 2008 to 2012. ‘Other’ cancers refers to all cancers, except those included in the model (breast, cervical, colorectal, leukaemia, liver, oesophageal, prostate, and stomach).

Source: IARC.^6^

**D. Additional results**

**Table S2.4. Number of people aged 18 and older in Kenya from 2018 to 2035 by age and HIV status.**

*Abbreviation: PLHIV, people living with HIV.*

| Age Groups | 2018 |  | 2020 |  | 2025 |  | 2030 |  | 2035 |  |
| --- | --- | --- | --- | --- | --- | --- | --- | --- | --- | --- |
|  | **PLHIV** | **HIV-negative** | **PLHIV** | **HIV-negative** | **PLHIV** | **HIV-negative** | **PLHIV** | **HIV-negative** | **PLHIV** | **HIV-negative** |
| 18-29 | 435,244 | 10,565,626 | 452,055 | 11,111,776 | 504,234 | 12,857,461 | 520,478 | 14,247,764 | 518,797 | 15,331,701 |
| 30-39 | 437,197 | 6,526,369 | 449,483 | 6,905,818 | 500,060 | 7,547,335 | 578,927 | 8,460,430 | 672,313 | 9,875,424 |
| 40-49 | 322,792 | 3,609,837 | 364,169 | 4,085,268 | 457,664 | 5,385,157 | 526,254 | 6,464,900 | 588,750 | 7,103,322 |
| 50-59 | 133,702 | 1,837,009 | 158,478 | 2,018,127 | 251,149 | 2,686,186 | 368,859 | 3,687,593 | 471,002 | 4,895,221 |
| 60+ | 74,734 | 1,828,614 | 87,413 | 1,949,539 | 132,994 | 2,308,992 | 204,848 | 2,820,936 | 317,069 | 3,609,271 |
| Total | **1,403,669** | **24,367,455** | **1,511,598** | **26,070,528** | **1,846,101** | **30,785,131** | **2,199,366** | **35,681,623** | **2,567,931** | **40,814,939** |

**Table S2.5.** **Detailed estimates of prevalent NCDs by calendar time and HIV status, as generated by the model.**

**in the last 12 months.*

*Abbreviations: CKD, chronic kidney disease, n, number; PLHIV, people living with HIV.*

| Age Groups | 2018 |  | 2020 |  | 2025 |  | 2030 |  | 2035 |  |
| --- | --- | --- | --- | --- | --- | --- | --- | --- | --- | --- |
|  | **Cases *(n)*** | **Prevalence (%)** | **Cases *(n)*** | **Prevalence (%)** | **Cases *(n)*** | **Prevalence (%)** | **Cases *(n)*** | **Prevalence (%)** | **Cases *(n)*** | **Prevalence (%)** |
| CKD |  |  |  |  |  |  |  |  |  |  |
| PLHIV |  |  |  |  |  |  |  |  |  |  |
| 18-30 | 14,312 | 3·29% | 14,873 | 3·29% | 17,414 | 3·45% | 18,675 | 3·59% | 18,551 | 3·58% |
| 30-39 | 33,646 | 7·70% | 34,271 | 7·62% | 38,288 | 7·66% | 44,666 | 7·72% | 52,551 | 7·82% |
| 40-49 | 38,761 | 12·01% | 44,573 | 12·24% | 56,675 | 12·38% | 65,159 | 12·38% | 73,389 | 12·47% |
| 50-59 | 20,478 | 15·32% | 24,675 | 15·57% | 40,127 | 15·88% | 59,931 | 16·25% | 77,054 | 16·36% |
| 60+ | 11,481 | 15·36% | 13,481 | 15·42% | 20,886 | 15·70% | 33,049 | 16·13% | 52,194 | 16·46% |
| Total | **118,678** | **8·5%** | **131,873** | **8·7%** | **173,390** | **9·4%** | **221,480** | **10·1%** | **273,739** | **10·7%** |
| HIV-negatives |  |  |  |  |  |  |  |  |  |  |
| 18-30 | 186,380 | 1·76% | 195,000 | 1·75% | 227,640 | 1·77% | 259,415 | 1·82% | 280,816 | 1·83% |
| 30-39 | 329,873 | 5·05% | 350,967 | 5·08% | 386,019 | 5·11% | 430,838 | 5·09% | 504,411 | 5·11% |
| 40-49 | 314,525 | 8·71% | 358,405 | 8·77% | 478,222 | 8·88% | 582,219 | 9·01% | 645,008 | 9·08% |
| 50-59 | 235,279 | 12·81% | 259,616 | 12·86% | 348,637 | 12·98% | 482,594 | 13·09% | 646,945 | 13·22% |
| 60+ | 262,177 | 14·34% | 278,992 | 14·31% | 330,968 | 14·33% | 406,156 | 14·40% | 523,182 | 14·50% |
| Total | **1,328,234** | **5·5%** | **1,442,980** | **5·5%** | **1,771,486** | **5·8%** | **2,161,222** | **6·1%** | **2,600,362** | **6·4%** |
| Depression* |  |  |  |  |  |  |  |  |  |  |
| PLHIV |  |  |  |  |  |  |  |  |  |  |
| 18-30 | 12,108 | 2·78% | 12,592 | 2·79% | 14,299 | 2·84% | 14,788 | 2·84% | 14,975 | 2·89% |
| 30-39 | 20,640 | 4·72% | 21,056 | 4·68% | 23,295 | 4·66% | 26,919 | 4·65% | 31,511 | 4·69% |
| 40-49 | 16,327 | 5·06% | 18,504 | 5·08% | 22,271 | 4·87% | 24,711 | 4·70% | 27,380 | 4·65% |
| 50-59 | 2,590 | 1·94% | 2,958 | 1·87% | 4,697 | 1·87% | 6,801 | 1·84% | 9,082 | 1·93% |
| 60+ | 2,522 | 3·37% | 3,019 | 3·45% | 4,621 | 3·47% | 7,214 | 3·52% | 10,775 | 3·40% |
| Total | **54,187** | **3·9%** | **58,129** | **3·8%** | **69,183** | **3·7%** | **80,433** | **3·7%** | **93,723** | **3·6%** |
| HIV-negative |  |  |  |  |  |  |  |  |  |  |
| 18-30 | 242,934 | 2·30% | 254,682 | 2·29% | 295,075 | 2·29% | 328,982 | 2·31% | 354,843 | 2·31% |
| 30-39 | 292,325 | 4·48% | 310,949 | 4·50% | 341,848 | 4·53% | 378,932 | 4·48% | 443,937 | 4·50% |
| 40-49 | 182,087 | 5·04% | 205,607 | 5·03% | 267,015 | 4·96% | 311,160 | 4·81% | 333,836 | 4·70% |
| 50-59 | 34,653 | 1·89% | 37,953 | 1·88% | 50,323 | 1·87% | 69,448 | 1·88% | 92,548 | 1·89% |
| 60+ | 69,320 | 3·79% | 74,405 | 3·82% | 91,500 | 3·96% | 110,296 | 3·91% | 135,694 | 3·76% |
| Total | **821,319** | **3·4%** | **883,596** | **3·4%** | **1,045,761** | **3·4%** | **1,198,818** | **3·4%** | **1,360,858** | **3·4%** |
| Diabetes, Type 2 |  |  |  |  |  |  |  |  |  |  |
| PLHIV |  |  |  |  |  |  |  |  |  |  |
| 18-30 | 3,146 | 0·72% | 3,196 | 0·71% | 3,741 | 0·74% | 4,072 | 0·78% | 4,130 | 0·80% |
| 30-39 | 10,007 | 2·29% | 10,357 | 2·30% | 11,723 | 2·34% | 13,224 | 2·28% | 15,403 | 2·29% |
| 40-49 | 14,253 | 4·42% | 16,423 | 4·51% | 21,072 | 4·60% | 24,676 | 4·69% | 27,903 | 4·74% |
| 50-59 | 9,739 | 7·28% | 11,320 | 7·14% | 18,329 | 7·30% | 27,357 | 7·42% | 35,565 | 7·55% |
| 60+ | 6,234 | 8·34% | 7,413 | 8·48% | 11,328 | 8·52% | 17,550 | 8·57% | 27,242 | 8·59% |
| Total | **43,379** | **3·1%** | **48,709** | **3·2%** | **66,193** | **3·6%** | **86,879** | **4·0%** | **110,243** | **4·3%** |
| HIV-negative |  |  |  |  |  |  |  |  |  |  |
| 18-30 | 66,548 | 0·63% | 69,626 | 0·63% | 81,429 | 0·63% | 92,831 | 0·65% | 99,933 | 0·65% |
| 30-39 | 145,950 | 2·24% | 156,096 | 2·26% | 172,139 | 2·28% | 191,134 | 2·26% | 223,517 | 2·26% |
| 40-49 | 161,487 | 4·47% | 184,151 | 4·51% | 246,938 | 4·59% | 301,315 | 4·66% | 334,331 | 4·71% |
| 50-59 | 134,367 | 7·31% | 147,827 | 7·32% | 198,166 | 7·38% | 275,138 | 7·46% | 370,016 | 7·56% |
| 60+ | 152,029 | 8·31% | 162,403 | 8·33% | 193,360 | 8·37% | 237,442 | 8·42% | 306,273 | 8·49% |
| Total | **660,381** | **2·7%** | **720,103** | **2·8%** | **892,032** | **2·9%** | **1,097,860** | **3·1%** | **1,334,070** | **3·3%** |
| Hypertension |  |  |  |  |  |  |  |  |  |  |
| PLHIV |  |  |  |  |  |  |  |  |  |  |
| 18-30 | 46,279 | 10·63% | 48,308 | 10·69% | 56,658 | 11·24% | 60,125 | 11·55% | 59,667 | 11·50% |
| 30-39 | 118,986 | 27·22% | 122,274 | 27·20% | 136,643 | 27·33% | 158,684 | 27·41% | 185,590 | 27·60% |
| 40-49 | 138,115 | 42·79% | 156,808 | 43·06% | 198,464 | 43·36% | 229,180 | 43·55% | 257,103 | 43·67% |
| 50-59 | 72,869 | 54·50% | 87,033 | 54·92% | 140,641 | 56·00% | 208,362 | 56·49% | 267,284 | 56·75% |
| 60+ | 43,303 | 57·94% | 51,054 | 58·41% | 78,634 | 59·13% | 122,810 | 59·95% | 192,022 | 60·56% |
| Total | **419,552** | **29·9%** | **465,477** | **30·8%** | **611,040** | **33·1%** | **779,161** | **35·4%** | **961,666** | **37·4%** |
| HIV-negative |  |  |  |  |  |  |  |  |  |  |
| 18-30 | 615,835 | 5·83% | 642,810 | 5·78% | 747,638 | 5·81% | 849,417 | 5·96% | 920,246 | 6·00% |
| 30-39 | 1,192,466 | 18·27% | 1,270,016 | 18·39% | 1,397,597 | 18·52% | 1,550,956 | 18·33% | 1,811,425 | 18·34% |
| 40-49 | 1,151,235 | 31·89% | 1,304,389 | 31·93% | 1,730,277 | 32·13% | 2,100,338 | 32·49% | 2,317,788 | 32·63% |
| 50-59 | 869,600 | 47·34% | 954,810 | 47·31% | 1,270,343 | 47·29% | 1,744,212 | 47·30% | 2,325,881 | 47·51% |
| 60+ | 1,022,484 | 55·92% | 1,089,565 | 55·89% | 1,289,056 | 55·83% | 1,573,984 | 55·80% | 2,012,388 | 55·76% |
| Total | **4,851,620** | **19·9%** | **5,261,590** | **20·2%** | **6,434,911** | **20·9%** | **7,818,907** | **21·9%** | **9,387,728** | **23·0%** |
| High cholesterol |  |  |  |  |  |  |  |  |  |  |
| PLHIV |  |  |  |  |  |  |  |  |  |  |
| 18-30 | 14,138 | 3·25% | 14,692 | 3·25% | 16,947 | 3·36% | 18,525 | 3·56% | 18,798 | 3·62% |
| 30-39 | 40,463 | 9·26% | 41,847 | 9·31% | 46,789 | 9·36% | 53,499 | 9·24% | 62,693 | 9·32% |
| 40-49 | 45,067 | 13·96% | 50,814 | 13·95% | 64,052 | 14·00% | 74,350 | 14·13% | 83,511 | 14·18% |
| 50-59 | 24,976 | 18·68% | 29,707 | 18·75% | 47,215 | 18·80% | 69,620 | 18·87% | 89,644 | 19·03% |
| 60+ | 16,568 | 22·17% | 19,468 | 22·27% | 29,572 | 22·24% | 45,617 | 22·27% | 70,592 | 22·26% |
| Total | **141,212** | **10·1%** | **156,528** | **10·4%** | **204,575** | **11·1%** | **261,611** | **11·9%** | **325,238** | **12·7%** |
| HIV-negative |  |  |  |  |  |  |  |  |  |  |
| 18-30 | 306,327 | 2·90% | 320,134 | 2·88% | 371,940 | 2·89% | 422,996 | 2·97% | 458,518 | 2·99% |
| 30-39 | 596,255 | 9·14% | 635,244 | 9·20% | 698,827 | 9·26% | 775,925 | 9·17% | 904,228 | 9·16% |
| 40-49 | 503,881 | 13·96% | 571,075 | 13·98% | 755,832 | 14·04% | 912,785 | 14·12% | 1,007,090 | 14·18% |
| 50-59 | 347,754 | 18·93% | 381,896 | 18·92% | 507,820 | 18·90% | 698,245 | 18·93% | 930,865 | 19·02% |
| 60+ | 412,556 | 22·56% | 439,154 | 22·53% | 519,373 | 22·49% | 633,888 | 22·47% | 809,740 | 22·44% |
| Total | **2,166,773** | **8·9%** | **2,347,503** | **9·0%** | **2,853,792** | **9·3%** | **3,443,839** | **9·7%** | **4,110,441** | **10·1%** |

**Table S2.6.** **Cardiovascular disease incidence from 2020 to 2035 by HIV status.**

*Abbreviations: ASI, age-standardized incidence; PLHIV, People Living with HIV.*

| PLHIV | | | HIV-negatives | | |
| --- | --- | --- | --- | --- | --- |
|  | **2020-2025** |  |  | **2020-2025** |  |
| Cases | **Crude** | **ASI** | **Cases** | **Crude** | **ASI** |
| 5,966 | 584·0 | 822·7 | 98,612 | 771·0 | 754·2 |
|  | **2025-2030** |  |  | **2025-2030** |  |
| Cases | **Crude** | **ASI** | **Cases** | **Crude** | **ASI** |
| 8,846 | 664·3 | 826·8 | 122,490 | 752·0 | 757·0 |
|  | **2030-2035** |  |  | **2030-2035** |  |
| Cases | **Crude** | **ASI** | **Cases** | **Crude** | **ASI** |
| 12,586 | **734·1** | 814·9 | 153,691 | 772·1 | 759·7 |

**Table S2.7.** **Cancer incidence from 2020 to 2035 by HIV status and type of cancer.**

*Abbreviations: ASI, age-standardized incidence; PLHIV, People Living with HIV.*

| Cancer type | PLHIV | | | HIV-negatives | | |
| --- | --- | --- | --- | --- | --- | --- |
| 2020 to 2024 | | | | | | |
|  | **Cases** | **Crude** | **ASI** | **Cases** | **Crude** | **ASI** |
| Any cancer | 24,473 | 309·76 | 334·70 | 157,493 | 47·71 | 99·84 |
| Breast | 1,688 | 31·54 | 30·18 | 30,579 | 23·13 | 47·09 |
| Cervical | 15,669 | 295·97 | 241·58 | 21,454 | 16·22 | 37·48 |
| Colorectal | 612 | 7·04 | 6·52 | 13,398 | 5·13 | 10·92 |
| Leukaemia | 475 | 5·46 | 6·07 | 6,483 | 2·48 | 3·99 |
| Liver | 836 | 9·61 | 11·59 | 3,703 | 1·42 | 3·41 |
| Oesophagus | 1,121 | 12·89 | 17·83 | 16,832 | 6·45 | 17·06 |
| Prostate | 944 | 28·69 | 44·34 | 20,001 | 15·55 | 41·28 |
| Stomach | 311 | 3·58 | 5·73 | 8,107 | 3·10 | 8·64 |
| Other | 2,817 | 32·39 | 39·88 | 36,936 | 14·15 | 22·73 |
| 2025 to 2029 | | | | | | |
|  | **Cases** | **Crude** | **ASI** | **Cases** | **Crude** | **ASI** |
| Any cancer | 31,308 | 331·27 | 340·18 | 192,914 | 51·38 | 100·14 |
| Breast | 2,241 | 35·61 | 29·25 | 38,442 | 25·55 | 47·64 |
| Cervical | 19,331 | 312·45 | 239·05 | 26,671 | 17·73 | 37·52 |
| Colorectal | 810 | 7·93 | 6·63 | 16,536 | 5·57 | 10·90 |
| Leukaemia | 600 | 5·87 | 6·29 | 7,635 | 2·57 | 4·01 |
| Liver | 1,167 | 11·41 | 12·10 | 4,617 | 1·55 | 3·44 |
| Oesophagus | 1,648 | 16·12 | 18·52 | 21,000 | 7·07 | 17·22 |
| Prostate | 1,327 | 34·25 | 42·22 | 24,445 | 16·70 | 41·40 |
| Stomach | 484 | 4·73 | 6·28 | 9,990 | 3·36 | 8·67 |
| Other | 3,700 | 36·17 | 38·50 | 43,578 | 14·67 | 22·79 |
| 2030 to 2035 | | | | | | |
|  | **Cases** | **Crude** | **ASI** | **Cases** | **Crude** | **ASI** |
| Any cancer | 38,640 | 343·23 | 337·12 | 236,464 | 55·42 | 99·96 |
| Breast | 2,848 | 38·69 | 28·30 | 47,414 | 27·76 | 47·63 |
| Cervical | 22,747 | 316·03 | 231·55 | 33,190 | 19·43 | 37·51 |
| Colorectal | 1,054 | 8·80 | 6·50 | 20,121 | 5·96 | 10·77 |
| Leukaemia | 749 | 6·25 | 5·76 | 9,098 | 2·70 | 4·01 |
| Liver | 1,613 | 13·45 | 12·47 | 5,684 | 1·68 | 3·45 |
| Oesophagus | 2,301 | 19·20 | 17·77 | 26,494 | 7·85 | 17·30 |
| Prostate | 1,834 | 40·18 | 42·46 | 30,392 | 18·28 | 41·11 |
| Stomach | 655 | 5·46 | 5·75 | 12,219 | 3·62 | 8·48 |
| Other | 4,839 | 40·35 | 38·32 | 51,852 | 15·37 | 22·83 |

**E. Sensitivity analysis**

To assess uncertainty around our pooled estimates of the burden of included NCDs, we varied the age-specific incidence of each NCD by +/- 10%. Overall, there was little variation in both the projected prevalence of people living with 1+ NCDs and of the prevalence of each NCDs separately (Table S2.8).

**Table S2.8. Predicted number* (prevalence**) of people living with NCDs with baseline and +/-10% calculated incidence from pooled data.**

*Numbers are expressed in millions

**Reported proportion are crude

|  | Baseline | -10% | +10% |
| --- | --- | --- | --- |
| People with 1+ NCDs | | | |
| HIV-negative people in 2018 | 11·8 (50·6%) | 11·6 (49·5%) | 12·0 (51·6%) |
| HIV-negative people in 2035 | 22·0 (55·7%) | 21·6 (54·6%) | 22·3 (56·7%) |
| PLHIV in 2018 | 0·9 (62·2%) | 0·8 (59·6%) | 0·9 (62·8%) |
| PLHIV in 2035 | 1·8 (71·4%) | 1·8 (69·0%) | 1·8 (72·2%) |
| Cancer (all) | | | |
| HIV-negative people in 2018 | 0·1  (0·4%) | 0·1  (0·4%) | 0·1  (0·4%) |
| HIV-negative people in 2035 | 0·2  (0·5%) | 0·2  (0·4%) | 0·2  (0·5%) |
| PLHIV in 2018 | 0·02  (1·3%) | 0·01  (1·2%) | 0·02  (1·3%) |
| PLHIV in 2035 | 0·1  (2·4%) | 0·1  (2·4%) | 0·1  (2·4%) |
| Cardiovascular disease (ischaemic stroke + myocardial infarction) | | | |
| HIV-negative people in 2018 | 0·2  (0·6%) | 0·1  (0·5%) | 0·2  (0·7%) |
| HIV-negative people in 2035 | 0·3  (0·7%) | 0·3  (0·6%) | 0·3  (0·8%) |
| PLHIV in 2018 | 0.01  (0·7%) | 0·01  (0·6%) | 0·01  (0·7%) |
| PLHIV in 2035 | 0.03  (1·0%) | 0·02  (0·9%) | 0·03  (1·2%) |
| Chronic kidney disease | | | |
| HIV-negative people in 2018 | 1·3  (5·5%) | 1·2  (4·9%) | 1·5  (6·0%) |
| HIV-negative people in 2035 | 2·6  (6·4%) | 2·3  (5·7%) | 2·9  (7·0%) |
| PLHIV in 2018 | 0·1  (8·5%) | 0·1  (7·5%) | 0·1  (9·2%) |
| PLHIV in 2035 | 0·3  (10·7%) | 0·2  (9·5%) | 0·3  (11·8%) |
| Depression | | | |
| HIV-negative people in 2018 | 0·8  (3·4%) | 0·8  (3·4%) | 0·8  (3·4%) |
| HIV-negative people in 2035 | 1·4  (3·3%) | 1·4  (3·3%) | 1·4  (3·3%) |
| PLHIV in 2018 | 0·1  (3·7%) | 0·1  (3·7%) | 0·1  (3·7%) |
| PLHIV in 2035 | 0·1  (3·5%) | 0·1  (3·4%) | 0·1  (3·5%) |
| Diabetes (type 2) | | | |
| HIV-negative people in 2018 | 0·7  (2·7%) | 0·6  (2·4%) | 0·7  (3·0%) |
| HIV-negative people in 2035 | 1·3  (3·3%) | 1·2  (3·0%) | 1·5  (3·6%) |
| PLHIV in 2018 | 0·04  (3·1%) | 0·04  (2·8%) | 0·05  (3·4%) |
| PLHIV in 2035 | 0·1  (4·3%) | 0·1  (3·9%) | 0·1  (4·8%) |
| High total cholesterol | | | |
| HIV-negative people in 2018 | 2·2  (8·9%) | 2·0  (8·0%) | 2·4  (9·7%) |
| HIV-negative people in 2035 | 4·1  (10·1%) | 3·7  (9·1%) | 4·5  (11·0%) |
| PLHIV in 2018 | 0·1  (10·1%) | 0·1  (9·2%) | 0·2  (11·1%) |
| PLHIV in 2035 | 0·4  (12·7%) | 0·3  (11·4%) | 0·4  (14·0%) |
| Hypertension | | | |
| HIV-negative people in 2018 | 4·9  (19·9%) | 4·4  (17·9%) | 5·3  (22·0%) |
| HIV-negative people in 2035 | 9·4  (23·0%) | 8·4  (20·6%) | 10·3  (25·4%) |
| PLHIV in 2018 | 0·4  (29·9%) | 0·4  (27·0%) | 0·5  (33·8%) |
| PLHIV in 2035 | 1·0  (37·4%) | 0·9  (33·6%) | 1·1  (42·2%) |

**References**

1. Smit, M. *et al.* The growing burden of noncommunicable disease among persons living with HIV in Zimbabwe. *AIDS* **32**, 773–782 (2018).

2. United Nations, Department of Economic and Social Affairs, P. D. World Population Prospects - Population Division - United Nations. (2017).

3. Institute for Health Metrics and Evaluation. Global Burden of Disease - Kenya (2017). (2017).

4. UNAID. UNAIDS - Epidemic Projection Package. (2016).

5. Mangal, T. D. Joint estimation of CD4+ cell progression and survival in untreated individuals with HIV-1 infection. *Aids* **31**, 1073–1082 (2017).

6. IARC. *Cancer Incidence in Five Continents*. **XI**, (2012).

7. Walker, R. *et al.* Stroke incidence in rural and urban Tanzania: A prospective, community-based study. *The Lancet Neurology* **9**, 786–792 (2010).

8. Wilkins, E. *et al.* European Cardiovascular Disease Statistics 2017 edition. 192 (2017).

9. Worm, S. *et al.* Presence of the Metabolic Syndrome Is Not a Better Predictor of Cardiovascular. *Diabetes Care* **32**, (2009).

10. Smit, M. *et al.* Future challenges for clinical care of an ageing population infected with HIV: A modelling study. *The Lancet Infectious Diseases* **15**, 810–818 (2015).

11. Mocroft, A. *et al.* Estimated glomerular filtration rate, chronic kidney disease and antiretroviral drug use in HIV-positive patients. *Aids* **24**, 1667–1678 (2010).

12. Schouten, J. *et al.* Cross-sectional comparison of the prevalence of age-associated comorbidities and their risk factors between hiv-infected and uninfected individuals: The age H IV cohort study. *Clinical Infectious Diseases* **59**, 1787–1797 (2014).

13. Do, A. N. *et al.* Excess burden of depression among HIV-infected persons receiving medical care in the United States: data from the medical monitoring project and the behavioral risk factor surveillance system. *PLoS ONE* **9**, e92842 (2014).

14. Looker, K. J. *et al.* Evidence of synergistic relationships between HIV and Human Papillomavirus (HPV): systematic reviews and meta‐analyses of longitudinal studies of HPV acquisition and clearance by HIV status, and of HIV acquisition by HPV status. *Journal of the International AIDS Society* **21**, (2018).

15. Liu, G., Sharma, M., Tan, N. & Barnabas, R. HIV-positive women have higher risk of HPV infection, precancerous lesions, and cervical cancer. *AIDS* 1 (2018). doi:10.1097/QAD.0000000000001765

16. Hernández-Ramírez, R. U., Shiels, M. S., Dubrow, R. & Engels, E. A. Cancer risk in HIV-infected people in the USA from 1996 to 2012: a population-based, registry-linkage study. *The Lancet HIV* **4**, e495–e504 (2017).

17. Edwards, J. K. *et al.* HIV with non-communicable diseases in primary care in Kibera, Nairobi, Kenya: Characteristics and outcomes 2010-2013. *Transactions of the Royal Society of Tropical Medicine and Hygiene* **109**, 440–446 (2015).

18. Peck, R. *et al.* Decreased renal function and associated factors in cities, towns and rural areas of Tanzania: a community-based population survey. *Trop. Med. Int. Health* **21**, 393–404 (2016).

19. Stanifer, J. W. *et al.* The Epidemiology of Chronic Kidney Disease in Northern Tanzania: A Population-Based Survey. *PLoS ONE* **10**, e0124506 (2015).

20. Aillon, J.-L. *et al.* Prevalence, types and comorbidity of mental disorders in a Kenyan primary health centre. *Soc Psychiatry Psychiatr Epidemiol* **49**, 1257–1268 (2014).

21. Ambugo, E. A. Cross-country variation in the sociodemographic factors associated with major depressive episode in Norway, the United Kingdom, Ghana, and Kenya. *Social science & medicine (1982)* **113**, 154–160 (2014).

22. Jenkins, R. *et al.* Prevalence of common mental disorders in a rural district of Kenya, and socio-demographic risk factors. *International Journal of Environmental Research and Public Health* **9**, 1810–1819 (2012).

23. Jenkins, R. *et al.* Common mental disorder in Nyanza province, Kenya in 2013 and its associated risk factors -an assessment of change since 2004, using a repeat household survey in a demographic surveillance site. *BMC Psychiatry* **15**, 309 (2015).

24. Kwobah, E., Epstein, S., Mwangi, A., Litzelman, D. & Atwoli, L. PREVALENCE of psychiatric morbidity in a community sample in Western Kenya. *BMC Psychiatry* **17**, 30 (2017).

25. Maj, M. *et al.* WHO Neuropsychiatric AIDS study, cross-sectional phase I. Study design and psychiatric findings. *Arch. Gen. Psychiatry* **51**, 39–49 (1994).

26. Ndetei, D. M. *et al.* The prevalence of mental disorders in adults in different level general medical facilities in Kenya: a cross-sectional study. *Ann Gen Psychiatry* **8**, 1 (2009).

27. Nyongesa, M. K. *et al.* Neurocognitive and mental health outcomes and association with quality of life among adults living with HIV: a cross-sectional focus on a low-literacy population from coastal Kenya. *BMJ Open* **8**, e023914 (2018).

28. Ongeri, L. *et al.* Suicidality and associated risk factors in outpatients attending a general medical facility in rural Kenya. *J Affect Disord* **225**, 413–421 (2018).

29. Ayah, R. *et al.* A population-based survey of prevalence of diabetes and correlates in an urban slum community in Nairobi, Kenya. *BMC public health* **13**, 371 (2013).

30. Christensen, D. L. *et al.* Prevalence of glucose intolerance and associated risk factors in rural and urban populations of different ethnic groups in Kenya. *Diabetes research and clinical practice* **84**, 303–310 (2009).

31. Mathenge, W., Foster, A. & Kuper, H. Urbanization, ethnicity and cardiovascular risk in a population in transition in Nakuru, Kenya: a population-based survey. *BMC public health* **10**, 569 (2010).

32. Oti, S. O., van de Vijver, S. J. M., Agyemang, C. & Kyobutungi, C. The magnitude of diabetes and its association with obesity in the slums of Nairobi, Kenya: results from a cross-sectional survey. *Tropical medicine & international health : TM & IH* **18**, 1520–1530 (2013).

33. Ministry of Health. *Kenya STEPwise Survey for Non-Communicable Diseases and Risk Factors 2015 Report*. (2015).

34. Chege, P. Multiple cardiovascular disease risk factors in rural Kenya: Evidence from a health and demographic surveillance system using the WHO STEP-wise approach to chronic disease risk factor surveillance. *South African Family Practice* **58**, 54–61 (2016).

35. Haregu, T. N. *et al.* Interlinkage among cardio-metabolic disease markers in an urban poor setting in Nairobi, Kenya. *Global Health Action* **9**, 30626 (2016).

36. Carvalho, J. J. M. *et al.* Blood pressure in four remote populations in the INTERSALT Study. *Hypertension* **14**, 238–246 (1989).

37. Christensen, D. L. *et al.* Cardiovascular risk factors in rural Kenyans are associated with differential age gradients, but not modified by sex or ethnicity. *Annals of Human Biology* **43**, 42–49 (2016).

38. Etyang, A. O. *et al.* Clinical and epidemiological implications of 24-hour ambulatory blood pressure monitoring for the diagnosis of hypertension in kenyan adults: A population-based study. *Journal of the American Heart Association* **5**, (2016).

39. Gómez-Olivé, F. X. *et al.* Regional and Sex Differences in the Prevalence and Awareness of Hypertension: An H3Africa AWI-Gen Study Across 6 Sites in Sub-Saharan Africa. *Global Heart* **12**, 81–90 (2017).

40. Hendriks, M. E. *et al.* Hypertension in sub-Saharan Africa: cross-sectional surveys in four rural and urban communities. *PLoS ONE* **7**, e32638 (2012).

41. Irazola, V. E. *et al.* Hypertension Prevalence, Awareness, Treatment, and Control in Selected LMIC Communities. *Global Heart* **11**, 47–59 (2016).

42. Jenson, A., Omar, A. L., Omar, M. A., Rishad, A. S. & Khoshnood, K. Assessment of hypertension control in a district of Mombasa, Kenya. *Global Public Health* **6**, 293–306 (2011).

43. Joshi, M. D. *et al.* Prevalence of hypertension and associated cardiovascular risk factors in an urban slum in Nairobi, Kenya: A population-based survey. *BMC Public Health* **14**, 1177 (2014).

44. Kaduka, L. U. *et al.* Prevalence of Metabolic Syndrome among an Urban Population in Kenya. *Diabetes Care* **35**, 887–893 (2012).

45. Olack, B. *et al.* Risk factors of hypertension among adults aged 35–64 years living in an urban slum Nairobi, Kenya. *BMC Public Health* **15**, 1251 (2015).

46. Onyango, M. J., Kombe, I., Nyamongo, D. S. & Mwangi, M. A study to determine the prevalence and factors associated with hypertension among employees working at a call centre Nairobi Kenya. *Pan African Medical Journal* **27**, (2017).

47. Oti, S. O. *et al.* Outcomes and costs of implementing a community-based intervention for hypertension in an urban slum in Kenya. *Bulletin of the World Health Organization* **94**, 501–509 (2016).

48. Pastakia, S. D. *et al.* Screening for diabetes and hypertension in a rural low income setting in western Kenya utilizing home-based and community-based strategies. *Globalization and Health* **9**, 21 (2013).

49. Pastakia, S. D. *et al.* Impact of Bridging Income Generation with Group Integrated Care (BIGPIC) on Hypertension and Diabetes in Rural Western Kenya. *Journal of general internal medicine* **32**, 540–548 (2017).

50. Rasmussen, J. B. *et al.* Hemoglobin levels and blood pressure are associated in rural black africans. *American journal of human biology : the official journal of the Human Biology Council* **28**, 145–148 (2016).

51. Smith, M. T. *et al.* Elevated blood pressure in the developing world: a role for clinical pharmacists. *International Journal of Pharmacy Practice* **26**, 334–340 (2018).

52. van de Vijver, S. J. M., Oti, S. O., Agyemang, C., Gomez, G. B. & Kyobutungi, C. Prevalence, awareness, treatment and control of hypertension among slum dwellers in Nairobi, Kenya. *Journal of hypertension* **31**, 1018–1024 (2013).

53. van de Vijver, S. *et al.* Impact evaluation of a community-based intervention for prevention of cardiovascular diseases in the slums of Nairobi: the SCALE-UP study. *Global Health Action* **9**, 30922 (2016).

54. Temmerman, M. *et al.* Risk factors for human papillomavirus and cervical precancerous lesions, and the role of concurrent HIV-1 infection. *International journal of gynaecology and obstetrics: the official organ of the International Federation of Gynaecology and Obstetrics* **65**, 171–181 (1999).

55. de Vuyst, H. *et al.* Distribution of human papillomavirus in a family planning population in Nairobi , Kenya. *Sexually Transmitted Diseases* **30**, 137–142 (2003).

56. Yamada, R. *et al.* Human papillomavirus infection and cervical abnormalities in Nairobi, Kenya, an area with a high prevalence of human immunodeficiency virus infection. *J. Med. Virol.* **80**, 847–855 (2008).

57. De Vuyst, H. *et al.* The prevalence of human papillomavirus infection in Mombasa, Kenya. *Cancer Causes Control* **21**, 2309–2313 (2010).

58. Maranga, I. O. HIV Infection Alters the Spectrum of HPV Subtypes Found in Cervical Smears and Carcinomas from Kenyan Women. *The Open Virology Journal* **7**, 19–27 (2013).

59. Temmerman, M. *et al.* Risk factors for human papillomavirus and cervical precancerous lesions, and the role of concurrent HIV-1 infection. *International journal of gynaecology and obstetrics: the official organ of the International Federation of Gynaecology and Obstetrics* **65**, 171–181 (1999).

60. De Vuyst, H. *et al.* Prevalence and determinants of human papillomavirus infection and cervical lesions in HIV-positive women in Kenya. *Br. J. Cancer* **107**, 1624–1630 (2012).

61. de Vuyst, H. *et al.* Distribution of human papillomavirus in a family planning population in Nairobi , Kenya. *Sexually Transmitted Diseases* **30**, 137–142 (2003).

62. Luque, A. E. *et al.* Prevalence of human papillomavirus genotypes in HIV-1-infected women in Seattle, USA and Nairobi, Kenya: Results from the Women’s HIV Interdisciplinary Network (WHIN). *International Journal of Infectious Diseases* **14**, e810–e814 (2010).

63. Greene, S. A. *et al.* Effect of cryotherapy vs loop electrosurgical excision procedure (LEEP) on cervical disease recurrence among women with HIV and high-grade cervical lesions in Kenya: a randomized clinical trial. *JAMA* (2019 - under review).

64. Globocan. Cancer Today. (2018). Available at: http://gco.iarc.fr/today/home. (Accessed: 7th December 2018)

65. Rodriguez, A. C. *et al.* The natural history of human papillomavirus infection and cervical intraepithelial neoplasia among young women in the Guanacaste cohort shortly after initiation of sexual life. *Sexually Transmitted Diseases* **34**, 494–502 (2007).

66. Kelly, H. *et al.* Association of antiretroviral therapy with high-risk human papillomavirus, cervical intraepithelial neoplasia, and invasive cervical cancer in women living with HIV: a systematic review and meta-analysis. *The Lancet HIV* **5**, e45–e58 (2018).

67. Abraham, A. G. *et al.* Invasive Cervical Cancer Risk Among HIV-Infected Women. *JAIDS Journal of Acquired Immune Deficiency Syndromes* **62**, 405–413 (2013).

68. Campos, N. G. *et al.* *Cost-effectiveness of cervical cancer screening in women living with HIV in South Africa*. (2018). doi:10.1097/QAI.0000000000001778

69. Chesson, H. W., Dunne, E. F., Hariri, S. & Markowitz, L. E. The estimated lifetime probability of acquiring human papillomavirus in the United States. *Sexually Transmitted Diseases* **41**, 660–664 (2014).

70. Goldie, S. J. *et al.* Policy Analysis of Cervical Cancer Screening Strategies in Low-Resource Settings. **285**, 3107–3116 (2001).

71. Schlecht, N. F. *et al.* Human Papillomavirus Infection and Time to Progression and Regression of Cervical Intraepithelial Neoplasia. *Journal of the National Cancer Institute* **95**, 1336–1343 (2003).

72. Castle, P. E. *et al.* Short term persistence of human papillomavirus and risk of cervical precancer and cancer: population based cohort study. *Bmj* **339**, b2569–b2569 (2009).

73. Kenya National Bureau of Statistics. (2018). Available at: https://www.knbs.or.ke/.

74. Hernández-Ramírez, R. U., Shiels, M. S., Dubrow, R. & Engels, E. A. Cancer risk in HIV-infected people in the USA from 1996 to 2012: a population-based, registry-linkage study. *Lancet HIV* **4**, e495–e504 (2017).
